# The origin of early Acheulean expansion in Europe 700 ka ago: new findings at Notarchirico (Italy)

**DOI:** 10.1038/s41598-020-68617-8

**Published:** 2020-08-14

**Authors:** Marie-Hélène Moncel, Carmen Santagata, Alison Pereira, Sébastien Nomade, Pierre Voinchet, Jean-Jacques Bahain, Camille Daujeard, Antonio Curci, Cristina Lemorini, Bruce Hardy, Giacomo Eramo, Claudio Berto, Jean-Paul Raynal, Marta Arzarello, Beniamino Mecozzi, Alessio Iannucci, Raffaele Sardella, Ignazio Allegretta, Emanuela Delluniversità, Roberto Terzano, Pauline Dugas, Gwenolé Jouanic, Alain Queffelec, Andrea d’Andrea, Rosario Valentini, Eleonora Minucci, Laura Carpentiero, Marcello Piperno

**Affiliations:** 1 UMR 7194 HNHP (MNHN-CNRS-UPVD), Département Homme et Environnement, Muséum National d’Histoire Naturelle, 1 rue René Panhard, 75013 Paris, France; 2 PACEA, UMR CNRS 5199, Université de Bordeaux, Bât B2 Allée Geoffroy St Hilaire, 33615 Pessac Cedex, France; 3 École Française de Rome, Piazza Farnese, T00186 Rome, Italy; 4 CEA Saclay, UMR 8212, UVSQ et Université Paris-Saclay, Orme des Merisiers, Gif-sur-Yvette, France; 5 Dipartimento di Storia Culture Civiltà, Università di Bologna, Bologna, Italy; 6 Dipartimento di Scienze Della Terra, Sapienza Università di Roma, Rome, Italy; 7 LTFAPA Laboratory, Department of Classics, Sapienza University of Rome, P.le A.Moro 5, 00185 Rome, Italy; 8 Kenyon College, Gambier, OH USA; 9 Dipartimento di Scienze Della Terra e Geoambientali, Università Degli Studi di Bari “Aldo Moro”, 70125 Bari, Italy; 10 Institute of Archaeology, University of Warsaw, Warsaw, Poland; 11 Department of Human Evolution, Max Planck Institute for Evolutionary Anthropology, Leipzig, Germany; 12 Dipartimento di Studi Umanistici, Università Degli Studi di Ferrara, 44121 Ferrara, Italy; 13 Dipartimento di Scienze del Suolo, Della Pianta e Degli Alimenti, Università Degli Studi di Bari “Aldo Moro”, 70126 Bari, Italy; 14 PACEA-Transfert Sédimentologie & Matériaux, 162 Avenue du Dr. Schweitzer, 33600 AderaPessac, France; 15 Laboratoire Chrono Environnement, UMR CNRS 6249, Université de Bourgogne Franche Comté, 16 route de Gray, 25030 Besançon Cedex, France; 16 Università l’Orientale de Naples, CISA, Napoli, Italy; 17 Museo Archeologico “Biagio Greco”, Mondragone, Italy

**Keywords:** Archaeology, Environmental sciences, Evolution, Palaeontology

## Abstract

Notarchirico (Southern Italy) has yielded the earliest evidence of Acheulean settlement in Italy and four older occupation levels have recently been unearthed, including one with bifaces, extending the roots of the Acheulean in Italy even further back in time. New ^40^Ar/^39^Ar on tephras and ESR dates on bleached quartz securely and accurately place these occupations between 695 and 670 ka (MIS 17), penecontemporaneous with the Moulin-Quignon and la Noira sites (France). These new data demonstrate a very rapid expansion of shared traditions over Western Europe during a period of highly variable climatic conditions, including interglacial and glacial episodes, between 670 and 650 (i.e., MIS17/MIS16 transition). The diversity of tools and activities observed in these three sites shows that Western Europe was populated by adaptable hominins during this time. These conclusions question the existence of refuge areas during intense glacial stages and raise questions concerning understudied migration pathways, such as the Sicilian route.

## Introduction

Recent data in prehistory tend to break down the previously supposed clear boundary between anatomically "modern" humans (*Homo sapiens*), associated with modern cognitive abilities, and earlier hominin species, such as Neanderthals and their ancestors, *Homo heidelbergensis.* The earliest Acheulean *Homo heidelbergensis* groups arrived in Europe ca. 1.0–0.7 Ma ago, and were exposed to challenging environmental conditions, which may have stimulated new cultural responses. These included behavioral innovations that could have potentially derived from African or Levantine origins during these early time ranges. During this period, named the Mid-Pleistocene revolution, Northern Europe was characterized by constraining climatic conditions, especially during glacial stages (1.25–0.7 Ma). Major topographic and sea-crossing barriers constituted considerable challenges to the adaptability and migration of early human populations. These biological and environmental conditions need to be considered further in the light of much older than previously recognized cultural innovations in the European human evolution scheme.

The earliest Western European settlement, related to *Homo* sp., is now thought to be older than 1.2 Ma^1^. *Homo antecessor*, the earliest remains of *Homo* sp. in Western Europe, discovered in Spain at Atapuerca (1.2–0.8 Ma), could be a side branch of the *Homo* sp. clade located at the westernmost area of the Eurasian continent^2^, as recently confirmed by paleoproteomics^3^. This hominin would have no link with the subsequent hominin species. *Homo heidelbergensis *(defined from the Mauer mandible)^4^ is considered to be at the origin of significant behavioral changes, such as the onset of biface production (technological Mode 2, Acheulean culture)^5–7^. The emergence of the Acheulean in Western Europe is very late compared to initial occurrences in Africa (1.75 Ma)^8–10^ and the Levant (1.4–1.2 Ma). Rare manifestations indicate the emergence of such technologies earlier than 700 ka ago in the Mediterranean Basin (e.g., Barranc de la Boella, Spain)^11^. Over the past decade, fieldwork has shown that elaborate biface production appeared suddenly around 700 ka ago in the Northwest of Europe^5,12,13^. Despite this new information, the timing and characteristics of the earliest evidence of Acheulean groups in Western Europe are still poorly known.

Various scenarios have been proposed to explain the onset of Acheulean diffusion in Western Europe without accounting for this late emergence: (1) a local origin stemming from previous core-and-flake traditions, or (2) the introduction of new populations or the diffusion of new ideas^5,14^. These new populations would have adapted to new environments and biomasses during the Mid-Pleistocene revolution.

**Figure 1:**
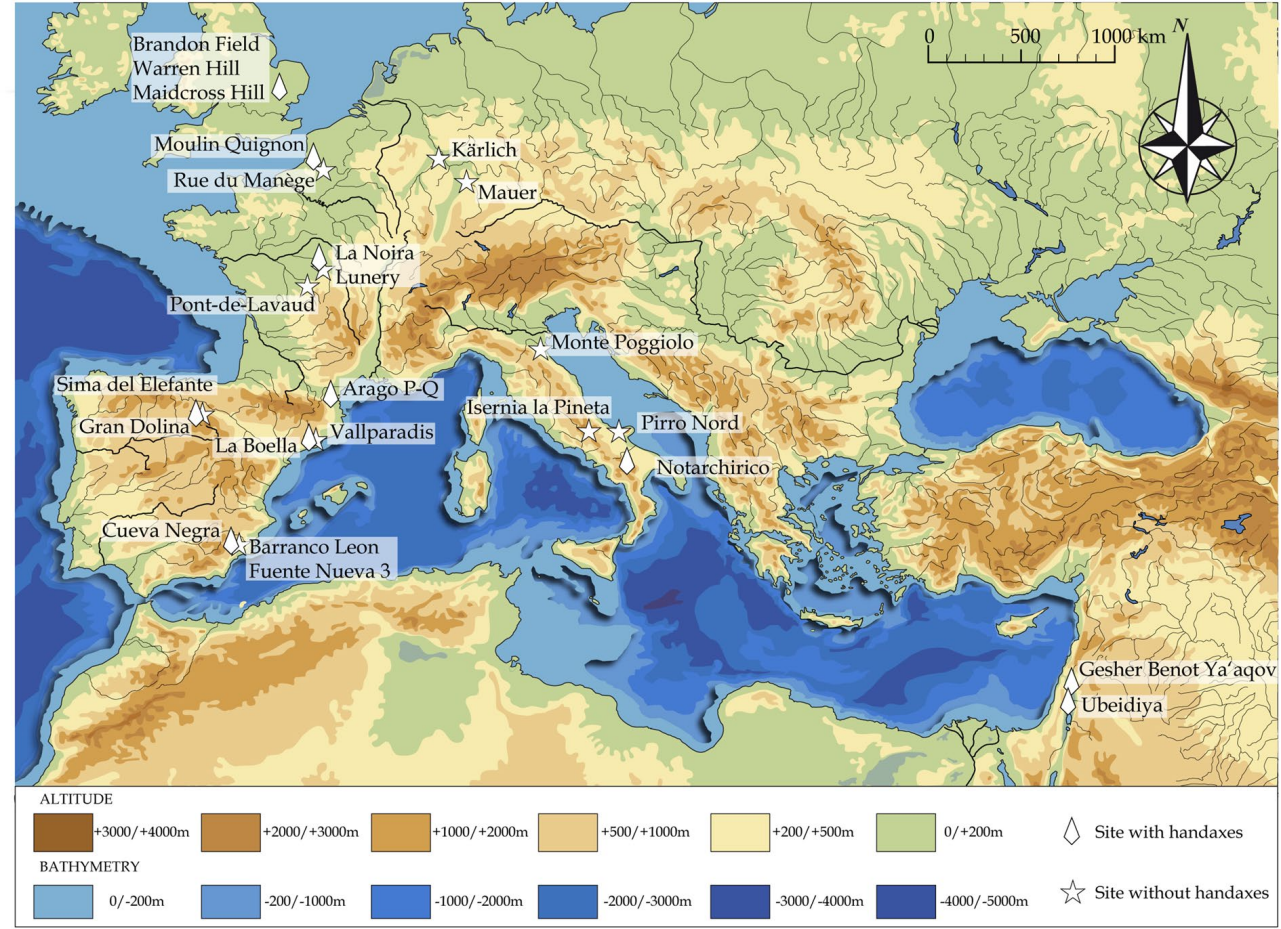
Notarchirico, the penecontemporaneous Acheulean sites of la Noira and Moulin Quignon, and other Western European sites dated between 1.4 Ma and 590 ka with or without bifaces. The sites are attributed to cores-and-flakes type sites (Mode 1), Acheulean sites or Lower Paleolithic sites (Mode 2). (Map drawn and created by P. Voinchet and M-H. Moncel).

The Basilicata region in Italy is characterized by the preservation of long archaeological sequences in volcano-sedimentary complexes linked to the eruptive activity of the Vulture stratovolcano^15,16^. Among these sites, the Notarchirico site (Venosa Basin), discovered in 1979, has yielded a 7 m-thick sequence of fluvial sediments including eleven archaeological levels, five of which contain bifaces (Fig. 1). A hominin femur fragment was found in the upper part of the sequence (level α)^17^. ^40^Ar/^39^Ar ages and ESR dates have revised the chronology of the sedimentary sequence excavated by Piperno and constrained the archaeological levels alpha to F between ca. 610 and 675 ka^18^, i.e., coeval with a glacial stage. The faunal assemblages from the upper levels A and α, described by Cassoli et al. can be attributed to Isernia faunal unit^19,20^. The archaeological material is found on pebbles andcobbles lags that represent shallow paleochannels. We present here new archaeological, chronological and environmental results for Notarchirico, which greatly enhance our understanding of the cognition of these hominins in Southern Italy earlier than 675 ka. These results fuel discussions concerning the timing of the earliest European Acheulean settlements in Western Europe, a behavioral shift by hominins in the region, and the possible routes used by hominins to access Southern Italy.

## Results

A 30-m-long trench was opened on the side of Notarchirico hill, at the base of the previously studied sequence. Excavations were conducted over a surface of 8 to 26 m^2^ depending on the stratigraphical unit investigated **(**Tables 1 and S1). Since 2016, excavation campaigns focusing on the base of the sequence have identified four units, including four archeosurfaces (I and J in addition to G and H).

**Table 1:** Synthesis of the sequence with location of archaeological layers, human behaviors and sampling.

Lithostratigraphic unit	Archeo unit	Sub-unit	Human behaviors	Characteristics	Samples (sedimentology NOT and geochronology Ar, ESR)
3	F (8 m^2^)		Bifaces, cores and flakes	Bed of cobbles-pebbles Cross-bedded volcano-derived and non-volcanic sands 20 cm Archaeological layer	
3		F1		Black volcanic sands 20 cm	NOT 17 01 Sample Ar
4.1				Brown with small gravel 1 m	NOT 17 02 NOT 17 03
4.2	G (10 m^2^)	G1	Bifaces, cores and flakes	Dark-grey volcanic sands 30 cm Archaeological layer	ESR sample
4.3				Coarse sandy sub-unit with cobbles and sub-angulous gravels 30 cm	NOT 17 04 NOT 17 05
5.1		H1a	Dispersed flakes	Silty-sandy deposit 10 cm Dispersed archeological material	NOT 17 06
5.2		H1b		Silty-sandy deposit 10 cm Dispersed archeological material	NOT 17 08 ESR sample
5.3	H (6 m^2^)	H1c	Dispersed flakes	Silty-sandy deposit. Sandier and oxidized with a few micro-beds of dark minerals 30 cm Dispersed archeological material	NOT 17 07 NOT 17 09 NOT 17 22 ESR sample
6.1	I (26 m^2^)	I1a-b-c	Cores and flakes	Local lenses of small pebbles 15–30 cm Coarse sands and beds of more or less dense gravels with pluri-millimetric anastomosed crusts 40–45 cm Dispersed archeological material	NOT 17 10 NOT 17 11 NOT 17 12 NOT 17 13 Sample Ar
6.2	I (26 m^2^)	I2	Cores and flakes	Dense accumulation of cobbles and smaller elements with limestones pebbles and a few fine-grained sandstone cobbles and flint nodules 10–15 cm Archaeological layer	NOT 17 14
7.1				Tuffaceous sub-unit 3 cm	NOT 17 15
7.2				Coarse yellow sands with a few cobbles 15 cm	NOT 17 16 Sample Ar
7.3	J (4 m^2^)	J1	Cores and flakes	Tephra-derived coarse sands with some cobbles 10 cm	NOT 17 17 Sample Ar
7.4	J (4 m^2^)	J2		Cobbles in a clayish volcano-derived matrix 30 cm	NOT 17 18 NOT 17 19
8.1				Light-grey sand and micro-breccia	NOT 17 20
8.2				Coarse yellow sands	NOT 17 21

### Lithostratigraphy and dynamics

**Figure 2:**
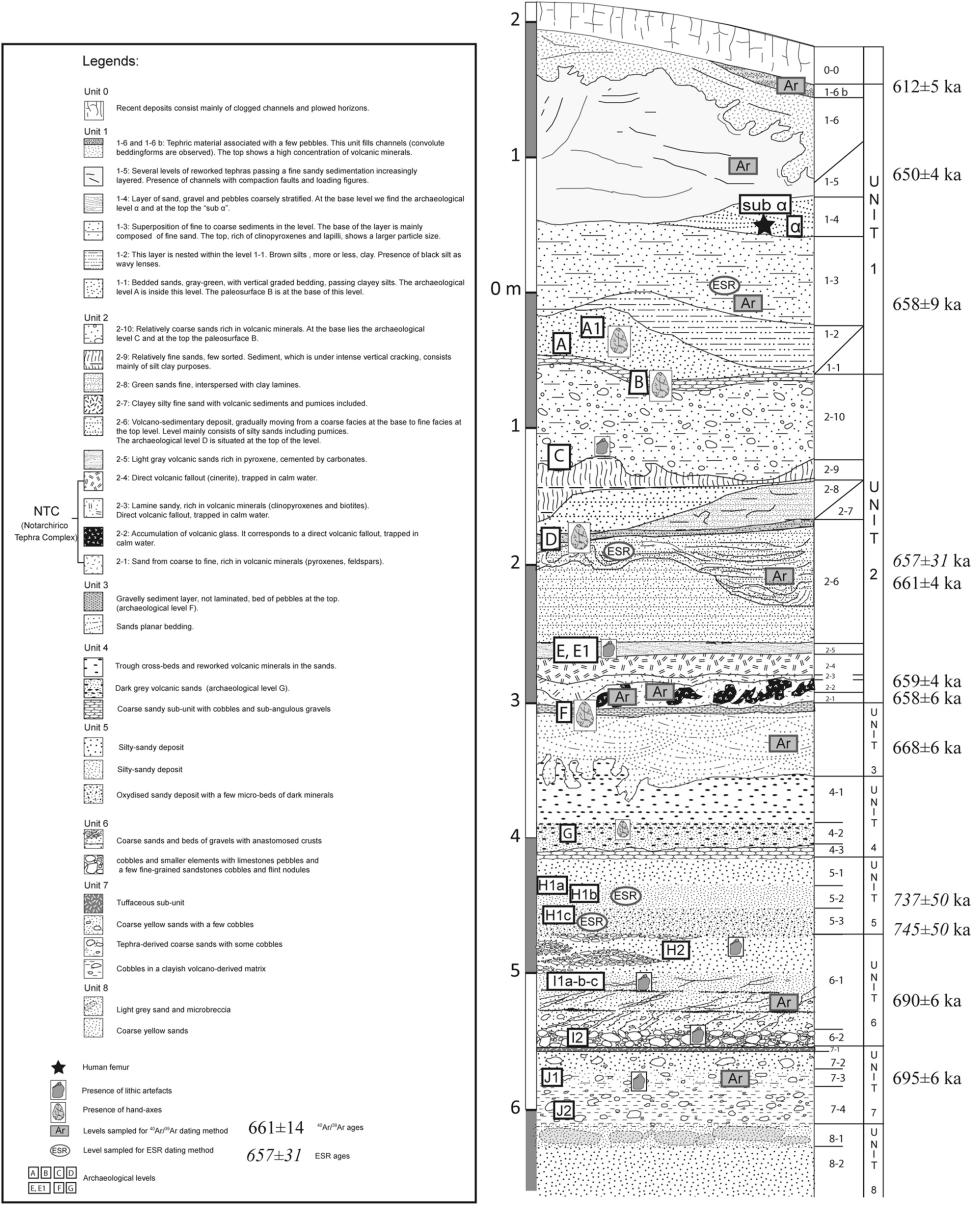
Stratigraphic log of the Notarchirico sequence (Modified from Raynal et al., 1999) with the 40Ar/39Ar and ESR dates on bleached quartz and the location of the phases of human occupation (with or without bifaces). The new fieldwork concerns the base of the sequence from layers F to J2. (Modified after J-P. Raynal and A. Pereira, by P. Voinchet and M-H. Moncel).

The studied deposits lie on top of the Piano Region sedimentary formation of the Venosa Basin^21^ (see Supplementary Tables S2, S3 and Figs. S1–S6). The sedimentary succession is described in detail in Supplementary Table S1 and Fig. 2 from the base upwards. The basal units (units 8 to 6) are characterized by low energy fluvial sedimentation and regular inputs of volcanic material. In the upper units (5 to 3), sedimentation progressively indicates somewhat higher energy currents and mainly volcano-derived remains. The main archaeological horizons are layers G (bottom of sub-unit 4.2), H (bottom of sub-unit 5.3), I1 (sub-unit 6.1), I2 (sub-unit 6.2) and J (sub-unit 7.4). The different facies correspond mainly to fills of meandering paleo-channels, crossed in places by the action of low energy currents. The fine components of these deposits derive from the alteration of volcanic fallout. The layers incorporating cobbles and gravels correspond to slope destabilization processes, which intervened after the arrival of masses of tephra and the release of lateral contributions from older conglomeratic deposits.

### Geochronology

Four sedimentary layers, some of which contain archaeological horizons, were dated by ^40^Ar/^39^Ar on single grains and ESR on bleached quartz. The stratigraphic positions of the geochronological samples are displayed in Fig. 2. Horizons H1b and H1c from sedimentary sub-unit 5.2 and 5.3 were dated by the ESR palaeodosimetric method, while the archaeological horizons I1 (sub-unit 6.1) and J (sub-unit 7.3) were analyzed using the^40^Ar/^39^Ar technique (Supplementary Table S4). ^40^Ar/^39^Ar and ESR ages are reported at 2σ analytical uncertainties hereafter. Fifteen sanidine crystals were hence individually dated by ^40^Ar/^39^Ar for sub-unit 6.1 and thirteen for sub-unit 7.3. In both cases, the probability diagrams are multimodal (Supplementary Fig. S7), characterized by populations of several crystals. At least six populations are evidenced for the youngest sub-unit 6.1, and 6/15 dated crystals indicate a weighted mean age of 690.3 ± 5.8 ka (MSWD = 1.8, P = 0.01). The ^40^Ar/^36^Ar ratio is not valid due to low spreading. The probability diagram associated with sub-unit 7.3 only shows two main crystal populations, as well as two older crystals. A weighted mean age of 695.2 ± 6.2 ka (MSWD = 2.8, P = 0.02) was calculated for the youngest population. As in the previous experiment, the inverse isochrone is not useable.

The ESR study was conducted with the multi-center approach advocated by Toyoda et al.^22^. Aluminum (Al) and Titanium (Ti–Li and Ti–H) ESR centers of quartz were measured in the present study. The ages derived from Ti–Li and Al centers are consistent with each other (Supplementary Table S4), whereas Ti–H ages are significantly younger. Mean ESR Al/Ti-Li ages of 743 ± 67 ka (2σ, MSWD = 0.024 and P = 0.88) and 736 ± 70 ka (2σ, MSWD = 0.013 and P = 0.91) were obtained for Notarchirico H1b (sub-unit 5.2) and Notarchirico H1c (sub-unit 5.3) samples respectively. We also note that the results of the H1 levels are much older than the ^40^Ar/^39^Ar ages of the over- and under-lying levels. However, these ages are still consistent, considering the associated error range.

### Palaeontology and taphonomy

**Figure 3:**
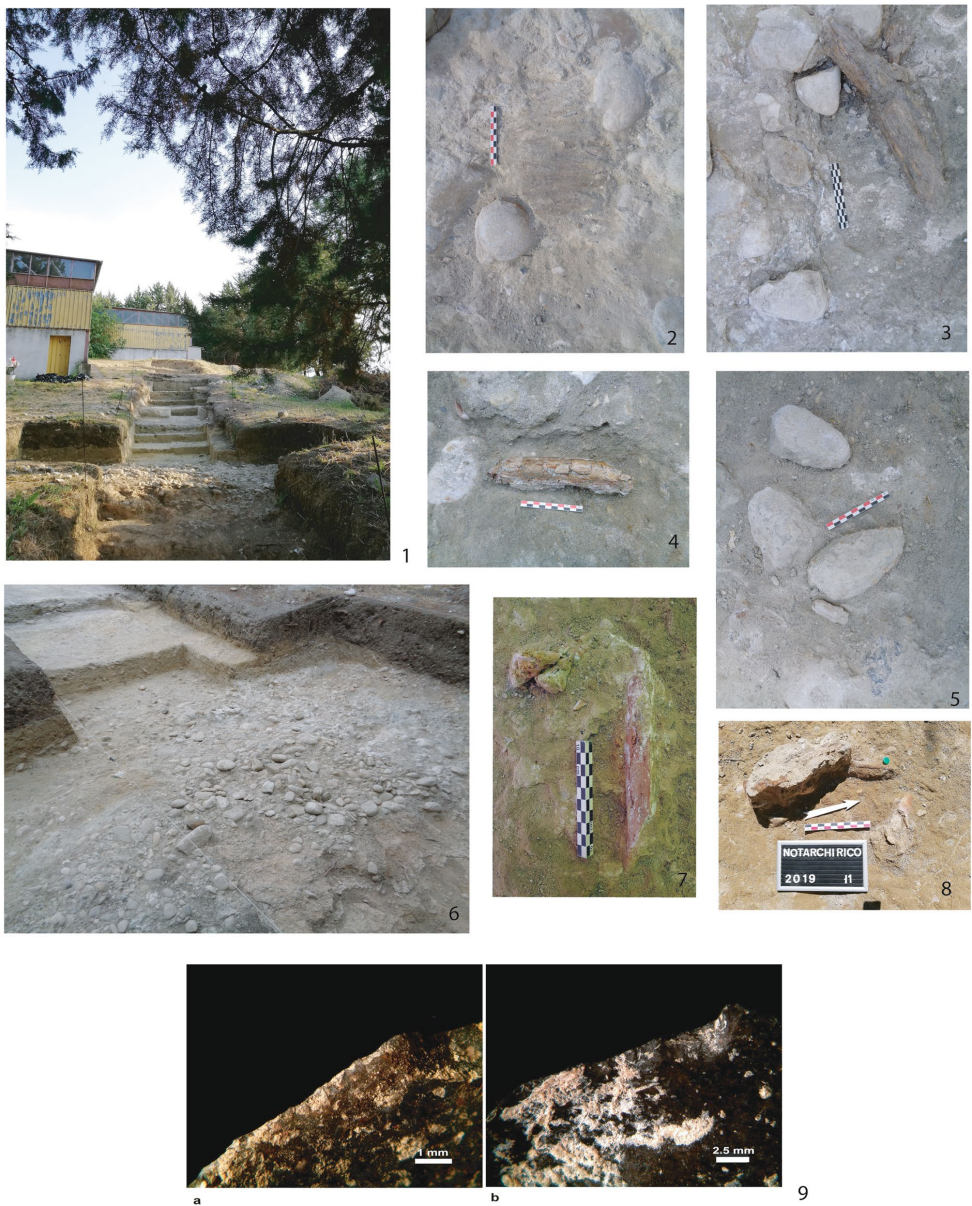
Illustrations of the new fieldwork at Notarchirico. (**1**) The 30 m-trench opened outside the building with the excavations of M. Piperno. (**2**) Layer F. Elephant tooth among the bed of pebbles. (**3**) Layer F. Fragment of elephant tusk and limestone pebble tools. (**4**) Layer G. Fragment of ungulate bone and pebble. (**5**) Layer F. Biface on a limestone pebble. (**6**) Aspect of layer I2, bed of pebbles. (**7**) Layer I2. Fragment of ungulate bone. (**8**) Layer I1. Fragment of bone. (**9**) (a) flake 2017 A18 F2: edge removals with feather/step terminations, diagonal unidirectional traces interpreted as resulting from cutting soft materials; (b) flake 2017 B19 F9: edge-removals with feather/step terminations, perpendicular direction interpreted as scraping soft/medium material. (Photos M-H. Moncel, C. Santagata n°1–8, n°9 C. Lemorini).

The lithic and bone material is dispersed on and included into the 10–30 cm-thick beds of pebbles-cobbles, remains of lakeshores or water channels (Fig. 3). There are no specific orientation patterns of the pieces and the pebbles. Fossil remains (NRT = 4,081 and NISPa = 289) are fragmented but can be attributed to several mammalian taxa (Supplementary Table S5). The straight-tusked elephant, *Palaeoloxodon antiquus,* was found in all stratigraphic levels, and is prevalent in levels F, G and I2. Cervids dominate the spectrum in levels H and I1, while large bovids are frequent in levels F and I2. Two species are newly reported: *Hippopotamus antiquus* in levels G and I1, and *Macaca sylvanus* spp. in level G. This occurrence of *Macaca sylvanus* spp. is documented by the proximal half of a right ulna (Supplementary Fig. S9). The fragmentary nature of the fossils complicates the detailed taxonomic attribution of cervids and bovids. Most of the remains consist of limb bone shafts, possibly related to slender bovids or megacerine deer. The occurrence of bison and/or aurochs is documented whereas *Bos primigenius* remains have not been confirmed^20,23^. *Praemegaceros sp.* is relatively abundant among cervids, alongside middle-sized cervids including *Dama* cf. *clactoniana* and *Cervus elaphus*. No carnivores have been found. This faunal list is almost similar to that of the youngest levels α and A of Notarchirico^15^.

Scarce small mammal remains were recovered in layer I and identified as *Arvicola mosbachensis, Microtus *(*Terricola*) cf. *M.* (T.) *arvalidens*, and *Microtus* cf. *M. nivaloides*. *Arvicola mosbachensis* is the most represented species and should be one of its earliest occurrences. The small micromammal assemblage is closely related to the previously published material, attributed to the beginning of the Early Toringian (*Arvicola-Microtus* zone, *Arvicola mosbachensis* subzone 3) (Supplementary Fig. S9).

Cervids are represented by all skeletal parts, but isolated teeth, ribs and small articular bones dominate. Cervids are mostly juveniles and subadults (Supplementary Tables S6–S9). Large-sized bovid remains consist mainly of teeth and metapodials. Prime-aged and old adults predominate. Elephants are mostly represented by tusk fragments, teeth and indeterminate bone fragments and the hippopotamus by four fragments of teeth, one of which belongs to a young individual from layer I1. Concerning the Voorhies groups, the relative scarcity of long bones and cranial remains and the abundance of short elements for all species and levels is consistent with a shore deposit or a low-energy deltaic and lacustrine environment, with some possible more violent hydraulic transport episodes (Supplementary Tables S10 and S11). Post-depositional dry bone fractures are the most recurrent type of fragmentation, but some green bone fractures associated with notches have been recorded. The anthropic or natural origin of the latter is difficult to determine, and they may possibly have been caused by natural violent impacts (trampling or strong water flow). Bone surfaces are badly preserved and practically no elements present carnivore marks (layer I1). No cut marks could be identified (Supplementary Tables S12–S14, Figs. S10–S12). The bone assemblages from the outside trench represent a mixture of multiple deposits of animal carcasses, the majority of which may have died naturally in the vicinity of this water channel/lacustrine context, and then secondarily transported, sorted and modified. As of yet, there is no evidence to support any clear human or carnivore contribution to the accumulation of the bone assemblage, but we cannot rule out the possibility that some animals may have been accumulated and/or consumed in situ by both types of predators and scavengers.

### Lithic assemblages

Artefacts are mainly on nodules of chert and various types of silicified limestone pebbles and cobbles, which are abundantly available along the paleochannels (Supplementary Fig. S13). Most of the nodules are cubic or slightly rounded, 10 mm to 35 mm long, with some larger exceptions (70–90 mm). Four main lithotypes were identified (Supplementary Figs. S14–S16): silicified litharenites (flysch chert), nodular chert, vitreous chert and radiolarite. The partial presence of a neocortex demonstrates the secondary origin of the raw materials, associated with the Flysch Rosso Fm primary source (Synthem of Palazzo San Gervasio). Silicified litharenites constitute 80% of the samples from layer F, and the rest consist of nodular chert. This distribution is different in layer G, where 50% of the samples are silicified litharenites, 25% nodular cherts and 25% radiolarites. In layers I1 and I2, silicified litharenites and nodular cherts are represented in similar proportions and some radiolarites and vitreous cherts were also used.

**Figure 4:**
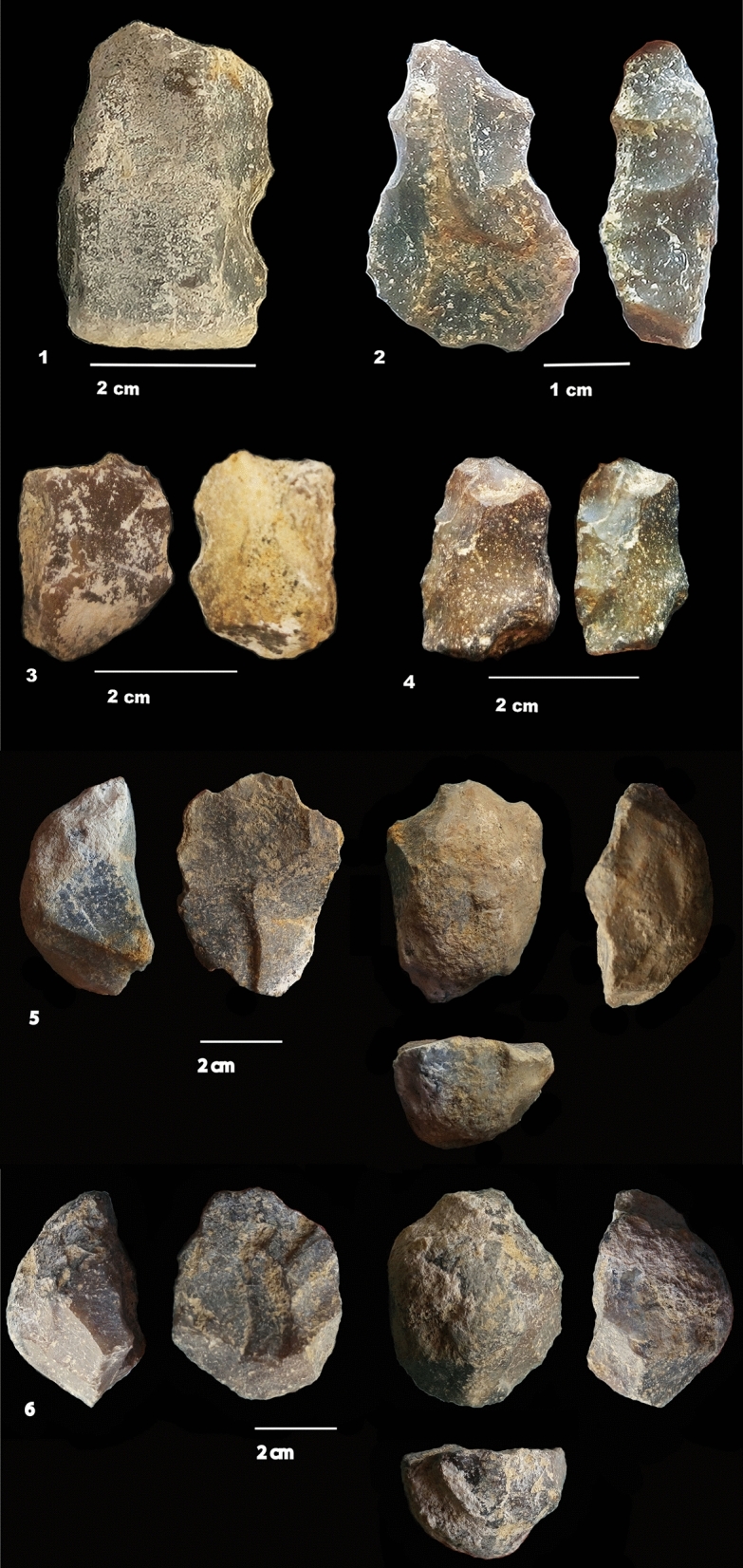
Artefacts on chert nodules. (**1**–**4**) small retouched nodules from layers G and I.Hominins selected small quadrangular nodules for a direct, abrupt and denticulate retouch. (**5**–**6**) unifacial discoid-type cores (layer I2). Removals are centripetal. The preparation of the striking platform is limited or absent. (Photos M-H. Moncel, C. Santagata).

**Figure 5:**
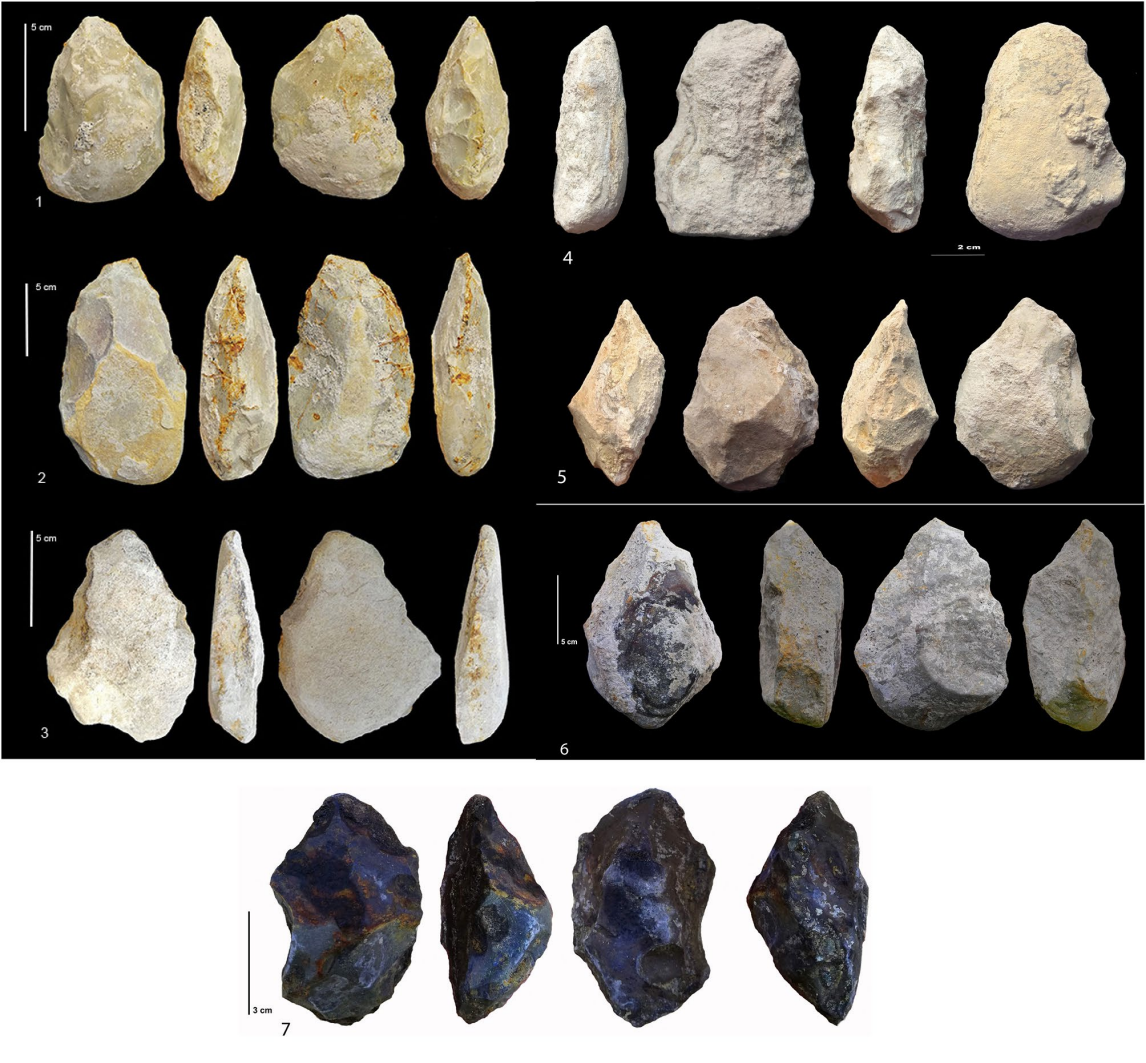
Layer F. Bifaces and bifacial tools on limestone pebbles (**1**–**6**). Bifacial tool on a large chert nodule. Four bifaces on limestone and large chert nodules were found among the ten Large Cutting Tools (LCTs). The shaping of the bifaces covers a large part of the periphery and surface of the tool by one or several series of removals with retouches sometimes regularizing the cutting edges. The cross-sections are symmetrical or plano-convex. The base is frequently cortical. The support is a cobble or a large flake in limestone (n°**1**,** 2**,** 5**). The only exception is a tool on a large chert nodule available in situ (n°**7**). Some evidence of recycling is observed on the cutting edges (second series of small removals or retouches). Crushing marks on edges, due perhaps to percussion, are sometimes visible. Detailed descriptions are given in the Supplementary data. (Photos M-H. Moncel, C. Santagata).

More than 900 lithic objects were recorded, always associated with faunal material (Supplementary Tables S15–S20, Figs. 3, 4, 5). The richest layers are F, G, I2 and I1 (Tables 1, 2). Layer H is poor, only composed of small flakes and flake-tools in chert. The new excavations have enriched the lithic series of layer F, previously comprised of relatively few artifacts, nine bifaces, and LCTs. Layer J has only yielded five flakes (including four retouched) and one chert core fragment. The material can be divided into two groups: flakes and cores on small chert nodules and a heavy-duty component mainly on limestone cobbles. Core technology consists of unifacial, unifacial discoid-type, multifacial and semi-rotating cores for layers F, G and I1. Less diversity is recorded in layer I2, which is only characterized by unifacial cores. Debitage utilizes the natural shape of nodules (except two flakes in layer G), with limited preparation of the striking platform, knapped by freehand percussion. The cores (typically 20–50 mm long) in chert produced low quantities of small 10–20 mm-long backed flakes (Supplementary Tables S15–S19 and Figs. S17–S25, Fig. 4). Cores sizes are similar between layers, but include some larger cores (Supplementary Figs. S26, S27). Flake platforms are cortical or flat, sometimes dihedral, punctiform or facetted (one in layer I2 to four in layer G), with an angle varying from 47 to 89°. Flake fragmentation is high. Flakes in chert range between 10 and 30 mm long (Supplementary Figs. S17, S21, S22, S23, Table S20). In layers F and G, there are some larger flakes (40–120 mm) coming from an in situ debitage (outside the excavated area) or brought to the site. Flakes, flake fragments and small nodules are retouched (between 5 and 20% of the corpus) on one or several edges by marginal, abrupt or denticulate retouch (Table 3). Some convergent tools exist (Supplementary Fig. S18). Flake-tools of layer G show the highest intensity of retouch on a large part of the periphery of flakes or nodules. The angle of the cutting edge is higher (75°–90°) for retouched nodules than for retouched flakes (25°–80°). The retouch on nodules is often abrupt and denticulate. We note that retouch substantially modifies initial blank shape in many cases. Retouched nodules in chert measure between 20 and 40 mm. In layers I2 and I1, most of the flakes are very small (10–20 mm) and retouched nodules are longer and wider (20–40 mm). Nodules in layer I2 are smaller than in layer I1. Nodules are generally thin (around 10 mm thick), but thicker than the flakes (around 2–5 mm for the most). Flakes and quadrangular nodules in chert could be considered as complementary tool kit in size.

**Table 2:** Heavy-duty component on limestone pebbles per archaeological layer (number) and flakes in other stones.

Layer	J	I2	I1	H	G	F
Unifacial convergent LCT tools		2			6	5
Bifaces					2	4
Unifacial pebble tools	2	5	9	1	15	34
Bifacial pebble tools		1	2		2	6
Pointed unifacial pebble tools			2		6	10
Pointed bifacial pebble tools/LCTs			1			4
Trifacial pebble tools			1		1	
Rabots on pebbles		1			2	5
Quadrangular unifacial tools						2
Broken pebbles with impacts + isolated removals			1		31	52
Flakes		2	2		7	46
Other stone products		1	5	1		4

**Table 3:** The tool kit on chert: number of flakes, fragments of flakes, retouched nodules and heavy-duty tools per archaeological layer.

Layer	J	I2	I1	H	G	F
Unretouched flake	5 (4 retouched)	40 (18 retouched)	98 (9 retouched)	21 (7 retouched)	78 (33 retouched)	177 (29 retouched)
Broken flakes-debris		9 (3 retouched)	95 (10 retouched)	19 (6 retouched)	78 (23 retouched)	66 (7 retouched)
Retouched nodules		4	21	1	50	12
Cores	1	6	10		25	10
Bifacial tools					1	1
Bifaces						1

Evidence of debitage on limestone pebbles is rare. The flakes in limestone (50–100 mm) are cortical and could come from the shaping process. The heavy-duty component is characterized by diversified and poorly-standardized artefacts. It includes unifacial round and thin pebble tools with limited shaping, some of which are pointed. Some tools are trifacial or quadrangular and extend over a large portion of the pebble. For layer I1, the rare bifacial pebble tools are small, and seldom pointed, while in layer I2, pebble tools are mostly unifacial.

**Figure 6:**
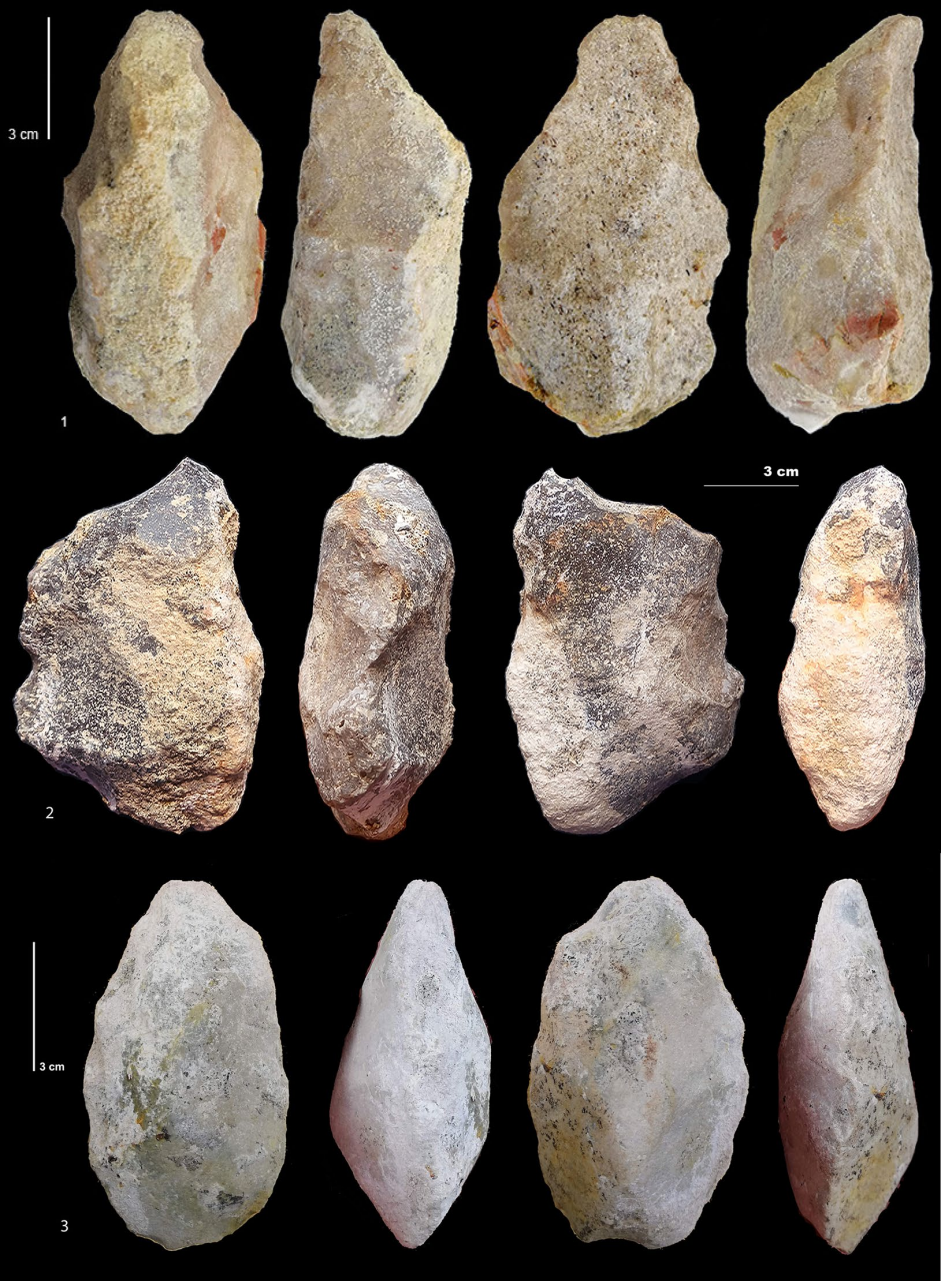
Layer G. (**1**) Recycled bifacial or trifacial tool on a limestone pebble. (**2**) Bifacial tool on a large chert nodule. (**3**) Biface on limestone pebble. Two limestone bifaces were also discovered in layer G, including one recycled specimen. As with the bifaces of layer F, they demonstrate a bifacial volume management and equilibrium of the two faces of the tool, as for the tools of layer F. The n°**1** shows a triface/biface on a pebble. It is a recycled tool with centripetal smooth removals on one flat face (lower face). The second phase of shaping used the piece as a preform to shape the opposite face by abrupt and more or less invasive removals on both lateral edges and the proximal extremity. There are crushing marks on the round tip, which is slightly shaped. The second biface is also on a pebble (n°** 3**). Shaping is alternate managing the whole volume by invasive and small removals to create a symmetrical tool. The shape is symmetrical. There is also one bifacial tool on a large chert nodule (n°** 2**). Using the natural shape of the nodule, the shaping is made by bifacial invasive convergent removals on one side and the proximal part form a tool that is asymmetrical in shape and cross-section, preserving a back on the opposite side. A back is preserved on the opposite side. There is with a notch related to a bec at the tool extremity. Detailed descriptions are given in the Supplementary data. (Photos M-H. Moncel, C. Santagata).

The lack of bifaces in some layers of the upper part of the sequence and in layers I1 and I2 at Notarchirico, as the highest number of bifaces in layer F, could be due to the activities or the size of the excavation. It cannot be ruled out that future excavations will provide bifaces in layers I1 and I2 at Notarchirico, further postponing the age of this technology already elaborated. Bifaces demonstrating overall volume management were observed in layer F, confirming discoveries made by M. Piperno’s excavations (Figs. 5, 6, Supplementary Table S21, Fig. 4, 5)^24^. In layer F, four bifaces on limestone and large chert nodules were found among the ten Large Cutting Tools (LCTs). The shaping of the bifaces covers a large part of the periphery and surface of the tool by one or several series of removals, with retouch sometimes regularizing the cutting edges. The cross-sections are symmetrical or plano-convex with the base frequently cortical. The support is a cobble or a large flake in limestone (Fig. 5, n°1, 2, 5). The only exception is a tool on a large chert nodule available in situ (Fig. 5, n°7). Some evidence of recycling is observed on the cutting edges (second series of small removals or retouches). Crushing marks on edges, due perhaps to percussion, are sometimes visible.

Two limestone bifaces were also discovered in layer G, including one recycled specimen. As with the bifaces of layer F, they demonstrate bifacial volume management and equilibrium of the two faces of the tool (Fig. 6). Figure 6, n°1 shows a triface/biface on a pebble. This is a recycled tool with centripetal smooth removals on one flat face (lower face) The second phase of shaping used the piece as a preform to shape the opposite face by abrupt and invasive removals on both lateral edges and the proximal extremity. There are crushing marks on the round tip, which is slightly shaped. The second biface is also on a pebble (Fig. 6, n°3). Shaping is alternate, managing the whole volume by invasive and small removals, to create a symmetrical tool. . There is also one bifacial tool on a large chert nodule (Fig. 6, n°2). Using the natural shape of the nodule, bifacial invasive convergent removals on one side and the proximal part form a tool that is asymmetrical in shape and cross-section, preserving a back on the opposite side, with a notch related to a bec at the tool extremity. Additional detailed descriptions of the LCTs and bifaces of Figs. 5 and 6 are given in the Supplementary data. Layers I1 and I2 did not yield bifaces, despite the fact that they were excavated over large surfaces (26 m^2^).

The chipped stone tools are affected by post-depositional processes (Supplementary Figs. S28, S29). However, use-related edge removals and edges are well preserved and clearly visible. A total of 42 active areas were detected on the lithic industry analyzed so far, including 32 from layer F (Fig. 3). Various soft to hard consistency materials were worked, but the best represented are soft and soft-medium materials, worked by cutting and scraping. In addition to these two actions, artefacts were also used for mixed actions, with a combination of transversal and longitudinal motions on the same tool. One example of engraving and one example of thrusting percussion were also recorded. The rare preserved micro-wear (only three cases) attests to processing fleshy tissue with mixed actions, scraping wood and cutting soft abrasive matter. Preliminary results of microscopic residue analysis support these conclusions with plant tissue and possible feather barbules co-occurring with wear patterns. Further analysis is underway to confirm these results (Supplementary Figs. S30 to S33). These data are consistent with the overall usewear data, which suggest that the chipped stone tools were used for different purposes, probably not only related to food processing. In fact, the scraping actions, mixed actions and engraving related to soft to hard materials may also represent activities to produce objects. Marked variations in the thickness of the active edges of these chipped stone tools (30°–80°) suggest that they were used for different activities requiring different active edge strengths.

## Discussion

New fieldwork at Notarchirico has yielded four archaeological layers in unit 3, dated to 668 ± 6 ka^18,24^. The ^40^Ar/^39^Ar ages calculated for sub-unit 6.1 and 7.3 are indistinguishable and point to the rapid deposition of the mainly fluvial sediment complex during the MIS 17 to the MIS 16 transition. The youngest eruptions identified in these levels correspond chronologically to the Toppo San Paolo sub-synthem of the Vulture stratovolcano (Barile synthem) dated in the literature to 679.6 ± 19 ka (Piano Regio Formation in the Venosa Basin), or to the Spinoritola sub-synthem (Foggianello synthem), dated to 693.8 ± 19 ka (Fonte del Comune Formation in the Venosa Basin) (ages recalculated from Villa and Buettner, 2009 with FCs standard at 28.294 Ma^25,26^. The chronological data are in keeping with lithostratigraphic and sedimentological analyses suggesting that unit 7 in particular is very similar to other localities in the Venosa Basin belonging to the Piano Regio sedimentary formation^16^.

Hominins took advantage of these paleo-channels to avail of local small nodules of chert and limestone cobbles, as well as large mammal carcasses. The latter appear to have died of natural causes as no clear cut marks or anthropic bone breakages were identified. Usewear on small flakes and nodules indicate (mixed actions (scraping wood and cutting soft material). Based on data from the usewear analysis, the chipped stone tools do not seem to have been “cutting oriented”, in contrast to other later small-sized Acheulean lithic industries^27^. Bifaces and bifacial tools present macro-traces on the edges resulting from working undetermined hard materials. Evidence of varied activities attests to diversified behavior and spatial exploitation. Some evidence of recycling of lithic materials is observed on one biface and some cores, suggesting recurrent hominin presence on the site. The gathering of small chert nodules and the production of very small flakes indicate adaptation to local raw materials or cultural traditions aiming to voluntary produce small end-products^27^.

Bifaces were also found lower in layer G, dated between 675 and 695 ka. Notarchirico thus yields the earliest bifaces in Italy and pushes back the age of the emergence of the Acheulean in Western Europe. Our study shows that hominins managed bifacial volumes, at least at the transition between MIS 17 and 16 in Italy, and that repeated human occupations occurred in both glacial and interglacial climatic conditions. The technological shift evidenced between layers G and I is not reflected by core technology, which is similar to core-and-flake assemblages (Mode 1) and some early Acheulean records (TD6 Atapuerca in Spain, prior to 780 ka, or Moulin-Quignon at 650 ka in the North of France)^13,28–31^, unlike other penecontemporaneous and younger sites, which show evidence of knapping innovation (e.g., la Noira 700 ka)^5,32–34^. The core technology on the whole sequence at Notarchirico, from level alpha to level F (excavations of M. Piperno)^13^, does not indicate differences over time and is similar to the technology applied in layers G and I at the bottom of the sequence. Small nodules of chert are used as cores which show mainly unifacial flaking and no preparation of the striking platform. Rare quartzite and quartz flakes and punctual knapping on limestone cobbles are present and may have been used for producing large flakes for shaping bifaces. Layers A, A1, B, D, F and G in the upper part of the sequence of the site yielded LCTs including some bifaces^24^. Layer F is the richest layer in quantity of bifaces and the new excavations confirm this richness. The bifaces found in the lowest level (layer G) are similar to those found in the layers of the upper part of the sequence. The management and reduction of the bifaces indicates a shaping process far from the simple and opportunistic process of the pebble-tools.

This ability to manage bifacial and bilateral symmetry, as well as the diversity of the morphological results, is also observed in penecontemporaneous Acheulean sites in North-western Europe between 700 and 600 ka at la Noira and Moulin-Quignon^5,13,35–41^ (Fig. 1). These sites yield bifaces and a mixture of diversified crudely-made bifacial tools, suggesting flexibility in the knapping processes of Middle Pleistocene populations. The combined use of small natural fragments of chert at Notarchirico suggests similar opportunism and adaptability to raw material constraints. Unlike Notrachirico, however, both soft and hard hammers were used for shaping siliceous rocks at la Noira and Moulin Quignon.

The new finds at Notarchirico contribute to the debate on the two current hypothesis for the origins of the Acheulean in Europe: (1) local evolution and innovation/invention^42^ or (2) arrivals of new populations/traditions (Acheulean) on the western side of Europe and limited to the area west of the Rhine River. For the first hypothesis, a local evolution, the site of Barranc de la Boella in Spain could represent an early attempt of bifacial shaping and local onset of crudely made bifacial tools (as in East Africa for the Early Acheulean)^36,37^. Partial bifacial shaping and the production of large flakes is rare in Western Europe and do not appear to represent a “transition” between simple pebble tools and Acheulean bifaces^40,41^. In contrast, Barranc de la Boella could also represent the arrival of a non-local hominin group and technology.

At la Noira, Moulin Quignon and Isernia-la-Pineta, we observe some innovations and complexity with some cores exhibiting a debitage independent of the stone shape. This behavior is not observed in Mode 1 series older than 700 ka. Raw material is mostly local but with little evidence of fragmentation of the reduction processes as in Acheulean sites in East Africa^10^.

Some Pebble-tools at Notarchirico, both in layers with and without bifaces, are pointed and bifacial. However, the shaping of these pebble-tools is always limited to a small part of the pebble and conforms to the stone shape. At la Noira, Moulin Quignon and Notarchirico, there are crudely-made bifacial tools with limited shaping, diversified and numerous heavy-duty components and a low ratio of bifaces. At Notachirico, as at Moulin Quignon and la Noira, the mode of shaping of the bifaces and LCTs indicates a technological shift and turning point at 700 ka, suggesting populations with different cognition and skills. Thus, a biface production appears by its complexity and specificity as an abrupt “invention” among the Western European series.

Notarchirico is located in the South of Italy and no early Acheulean sites are currently known in Northern/central Italy, except perhaps Isernia-la-Pineta that did not deliver bifaces^7^. Moreover, there is a chronological and behavioral gap between the “cores-and-flakes” series of Pirro Nord and Ca’Belvedere di Monte-Poggiolo, dated to around 1.4–1.0 Ma, and the bifacial technology at Notachircio^29,43,44^. The sites of la Noira, Moulin-Quignon and Notarchirico are key sites for attempting to resolve the timing of the onset of the European Acheulean. All three sites show evidence of elaborate early biface production in a short window of time, between 700 and 650 ka. They demonstrate shared technological traditions over a wide area of Western Europe, from southern Italy to northwestern France during both interglacial and glacial conditions^45–51^. Geochronological data available for Notarchirico indicate that the site was occupied by hominins between 690 and 600 ka, over a period covering the end of MIS 17, the entire MIS 16 and the beginning of MIS 15. No major changes were observed in the large mammals palaeontological record, except the presence of the hippopotamus, indicating a temperate humid climate, in levels G and I1, both chronologically attributed to the late MIS 17. On the other hand, the microfauna assemblage of layers or archaeosurfaces E and F, geochronogically coeval of the glacial MIS 16, indicates cold environments, in agreement with the few available palynological data dominated by grasses and *Pinus*^19^. It should be underlined that the available regional palaeoenvironmental data (as the Ohrid record in Albania)^50^ show in Mediterranean area the permanent presence of deciduous trees even during the glacial stages at the beginning of the Middle Pleistocene, which corresponded more to arid than very cold periods.

At Notarchirico, Acheulean assemblages are associated both with interglacial (MIS17) and glacial (MIS16) contextes, while at La Noira and Moulin Quignon, the human occupations seems both contemporaneous to early glacial stages^5,12,13,30,31^. These sites also show the adaptability of hominins not only to climatic variability, but to widely varying geography and types of raw materials^33,34,39,40,52,53^. New cognitive skills, along with a new and diversified tool kit, could have helped hominins to extend their territories when the climatic conditions were more favorable. Furthermore, hominins were able to occupy northern territories despite the lack of the fire use, even as conditions became colder^39,45–51,54^. The presence of hominins with similar bifacial technology over such a wide area of Western Europe could signal an increase in hominin populations or a high degree of mobility of hominin groups^55–60^.

At this point, the known Acheulean sites just after 700 ka in Western Europe suggests that southern areas did not just serve as refugia during glacial times even if milder than in North Western areas^39,41^.

In contrast to the penecontemporaneous Acheulean sites of La Noira and Moulin Quignon, Notarchirico is located far to the south. If we consider the possible pathways for the arrival of the Acheulean in this region, the location of Notarchirico in southern Italy suggests two possible paleogeography-dependent routes: (1) following the northern coasts of the Mediterranean Sea from the Levant during low sea levels in glacial phases, or (2) crossing the Sicily channel between Tunisia and Sicily, also during glacial periods with low sea levels. Given the evidence of probable hominin occupation of insular land masses in the Mediterranean during the Lower Paleolithic (Crete, Gavdos, and Naxos)^61^, along withrecent discoveries of evidence of settlement on Indonesian and Philippine Islands east of the Wallace line in Southeast Asia as early as 700 ka^62–66^, the Sicilian route deserves consideration, in spite of the absence of solid evidence of a land bridge.

## Methods

### Lithostratigraphy

Classical field and laboratory methods were used for this study. The names of formations and the definition of units follow international stratigraphic rules (Supplementary Raynal et al.). The sedimentological samples were mostly loose samples for grain-size analysis and some blocks for thin section investigation (J.-P. R.). They were processed for particle size analysis, geochemical analysis and thin section preparation at the PACEA laboratory (University of Bordeaux) (P.D. and A.Q.). A Horiba LA-950 laser particle size analyzer was used for the twenty samples for grain size analysis. Sample pretreatment included suspension in sodium hexametaphosphate (5 g/l) and hydrogen peroxide (35%), at room temperature for 12 h. The suspension was then subjected to 60 s ultrasounds to achieve optimal dispersion. The Mie solution to Maxwell's equation provided the basis for calculating particle size using a refractive index of 1.333 for water and 1.55i–0.01i for the particles. Grain size distribution expressed in ɸ units was decomposed into different Gaussian populations (parametric curve fitting method) to identify and quantify the main modes. Results are presented in Supplementary Tables S2 and S3 and in several diagrams). X-Ray fluorescence was used to characterize one sample after grinding and transformation into 300 mg pellets. X-ray fluorescence (ED-XRF) analysis was carried out using a portable SPECTRO X-SORT (40 kV, 50 μA). Measurements were recorded in an air path with an acquisition time of 300 s. The device was calibrated beforehand using ICP-AES/ICP-MS compositions from 26 samples of Neogene and Quaternary sediments obtained by the SARM-CRPG laboratory in Nancy. Only elements for which a correlation coefficient (R2) greater than 0.9 between ED-XRF and ICP-AES/ICP-MS values were taken into account. Elements lighter than Si are not detected with this device. Thin sections from three undisturbed sediment blocks were vacuum impregnated with polyester resin. The mineralogical content of thin sections was studied under the microscope in natural and polarized-analyzed light and minerals were identified by their optical characteristics (G. J.).

### Geochronology

Methodological protocols including preparation and analytical processes can be found in Supplementary Tables S4 and S5 for both methods. The ^40^Ar/^39^Ar weighted mean ages include all sources of uncertainties (neutron fluence gradient (J), as well as the total K decay constant^26^. Samples were co-irradiated with the monitor standard ACs-2 associated with an age of 1.1891 Ma^67^. Corrections for initial argon are based on an atmospheric composition of 298.56^68^.

### Faunal and lithic remains

The early Middle Pleistocene is characterized by major reorganizations of terrestrial ecosystems, occurring in successive phases during paleoenvironmental changes, and strongly influenced by the onset of 100 ka climate cyclicity. Dispersal phases, mainly related to the progressive diffusion in Italy of taxa from Eastern and Central Europe, led to mammal faunal renewal. The biochronology of the early Middle Pleistocene vertebrate faunas are related to the Galerian Mammal Age, which includes Ponte Galeria, Isernia and Fontana Ranuccio Faunal Units (Mecozzi et al., Supplementary).

Methods for taphonomic analysis are explained in detail in Supplementary Daujeard and Curci. We report the total number of skeletal remains (NRT), the number of identified specimens (NISP), the minimum number of elements (MNE) and the minimum number of individuals present (MNI). We recorded the dimensions (length, breadth and thickness) and anatomical, taxonomic, and modification data for all the recorded and identified specimens. Non-coordinated indeterminate fragments were only used for fragmentation studies (tissue types and size classes). Ontogenic age-at-death of prey specimens was based on dental eruption/replacement patterns and wear. We observed bone surfaces with the naked eye and we examined and photographed some elements to distinguish the various surface alterations using two Dino-Lite Digital Microscopes (AD7013MZT and AM7915MZT, magnification 20–220 ×).

Lithic analysis aimed to identify technological behaviors and reduction processes i.e.^69,70^ according to raw materials. Petrographic descriptions were carried out using a non-destructive multi-parametric protocol for chert investigations (NM-PCI), with a formalized mixed-data matrix (Supplementary Eramo et al.). The NM-PCI uses a formalized description of macroscopic and mesoscopic characteristics (e.g., structure, texture, fracture), expressed in binary or ordinal variables, as well as colorimetric (i.e., CIEL*a*b*) and geochemical (i.e., K, Ca, Ti, Mn, Fe, Ni, As, Sr, Ba) continuous variables of the chert matrix, acquired with portable devices (Konica-Minolta CM-2600d spectrophotocolorimeter and a Thermo-NITON XL3t ED-XRF spectrometer). A selection of the transformed mixed data variables^71^ was statistically processed and visualized (i.e., PAM, t-SNE) in R software environment^72^ with the following packages: cluster^73^ for data transformation, PAM algorithm and silhouette width; ggplot2^74^ and Rtsne^75^ for output visualization; and fpc^76^ for internal and external clustering validation.

Use-wear analysis^27^ included the Low-Power (LPA) and the High-Power Approaches (HPA) in order to obtain a detailed interpretation of the function of chipped stone tools (Supplementary Figs. S26, S27). The LPA analyses traces of use observable at low magnification (generally up to 70–80 ×), defined in the literature as macro-traces, which for flint artefacts consist of: edge removals (scars resulting from use) and edge rounding. The HPA analyses traces of use observable at high magnifications (at least 100 ×), defined in the literature as micro-traces, which for flint artefacts consist of micro edge rounding, polish and striations. The LPA was applied to the chipped stone tools of Notarchirico using a Nikon SMZ stereomicroscope with a magnification range of 0.75 to 7.5 × equipped with a 10 × ocular, a 1 × objective, a reflected light system and a ToupView CMOS. The LPA enabled us to obtain a first evaluation of the degree of preservation of the lithic industry, to sample the tools with macro-wear and to infer the activities carried out and the hardness of the processed materials. The HPA was applied with a Nikon Eclipse metallographic microscope, with 10 ×, 20 ×, 50 × oculars equipped with a ToupView CMOS Camera. Helicon Focus software was used for picture focus stacking. A digital microscope Hirox RH-2000 was also used to observe highly reflective surfaces and to obtain images with ultra-fine detail. Micro-wear observation was carried out at the LTFAPA laboratory on lithic surface molds made with an ultra-fine dual-component silicone Provil Novo Light Fast, Heraeus (licence paid by C. Lemorini). Use-wear was interpreted with reference to the comparative reference collection of flint replicas of the LTFAPA laboratory of the Sapienza University (Supplementary Lemorini).

## Supplementary information

Supplementary Information.
