# Supplementary material for: The origin of early Acheulean expansion in Europe 700 ka ago: new findings at Notarchirico (Italy)
Source: Sci Rep. 2020 Aug 14;10:13802. doi: 10.1038/s41598-020-68617-8 (PMC7429832; doi:10.1038/s41598-020-68617-8)
Supplement: Supplementary file 1 — Supplementary Information. [file 41598_2020_68617_MOESM1_ESM.pdf]

Supplementary information

The origin of early Acheulean expansion in Europe 700 ka ago: new findings at Notarchirico (Italy)

Marie-Hélène Moncel(1)*, Carmen Santagata(1, 2), Alison Pereira (1, 3), Sébastien Nomade(4), Pierre Voinchet(1), Jean-Jacques Bahain(1), Camille Daujeard(1), Antonio Curci(5), Cristina Lemorini(7), Bruce Hardy(8), Giacomo Eramo(9), Claudio Berto(10), Jean-Paul Raynal(2, 11), Marta Arzarello(12), Beniamino Mecozzi(6), Alessio Iannucci(6), Raffaele Sardella(6), Ignazio Allegretta (13), Emanuela Delluniversità (9), Roberto Terzano (13), Pauline Dugas(2), Gwenolé Jouanic(15), Alain Queffelec(14), Andrea d'Andrea(16), Rosario Valentini(16), Eleonora Minucci(16), Laura Carpentiero(16), Marcello Piperno(17)

1) UMR 7194 HNHP (MNHN-CNRS-UPVD), Département Homme et Environnement, Muséum National d'Histoire Naturelle, 1 rue René Panhard, F-75013 Paris, France.
2) PACEA, UMR CNRS 5199, Université de Bordeaux, Bât B2 Allée Geoffroy St Hilaire, 33615 Pessac cedex (France)
3) École Française de Rome, Piazza Farnese, IT-00186, Rome, Italy
4) CEA Saclay, UMR 8212, UVSQ et Université Paris-Saclay, Orme des Merisiers Gif-sur-Yvette, France.
5) Dipartimento di Storia Culture Civiltà, Università di Bologna, Italy
6) Dipartimento di Scienze della Terra, Sapienza Università di Roma, Italy
7) LTFAPA Laboratory, Department of Classics, Sapienza University of Rome, P.le A.Moro 5 00185 Rome, Italy
8) Kenyon College, Ohio, USA
9) Dipartimento di Scienze della Terra e Geoambientali, Università degli Studi di Bari "Aldo Moro", Bari, 70125, Italy
10) Institute of Archaeology, University of Warsaw, Poland
11) Department of Human Evolution, Max Planck Institute for Evolutionary Anthropology, Leipzig, Germany
12) Dipartimento di Studi Umanistici, Università degli Studi di Ferrara, 44121 Ferrara, Italy
13) Dipartimento di Scienze del Suolo, della Pianta e degli Alimenti, Università degli Studi di Bari "Aldo Moro", Bari, 70126, Italy
14) PACEA-Transfert Sédimentologie & Matériaux, Adera, 162 Avenue du Dr. Schweitzer, 33600 Pessac (France)
15) Laboratoire Chrono Environnement, UMR CNRS 6249, Université de Bourgogne Franche Comté, 16 route de Gray, F 25030 Besançon cedex (France)
16) Università L'Orientale de Naples, CISA, Italia
17) Museo archeologico "Biagio Greco", Mondragone, Italia

*corresponding author : marie-helene.moncel@mnhn.fr

Table S1. Detailed synthesis of the sequence with location of archaeological layers and sampling.

Lithostratigraphic Unit	Archeo unit	Sub-unit	Characteristics	Samples (sedimentology NOT and geochronology Ar, ESR)
3	F<br>(8 m²)		Cross-bedded volcano-derived and non-volcanic sands 20 cm<br>Archaeological layer	
3		F1	Black volcanic sands 20 cm	NOT 17 01<br>Sample Ar
4.1			Brown with small gravel 1m	NOT 17 02<br>NOT 17 03
4.2	G<br>(10 m²)	G1	Dark-grey volcanic sands 30 cm<br>Archaeological layer	ESR sample
4.3			Coarse sandy sub-unit with cobbles and sub-angulous gravels 30 cm	NOT 17 04<br>NOT 17 05
5.1		H1a	Silty-sandy deposit 10 cm<br>Dispersed archeological material	NOT 17 06
5.2		H1b	Silty-sandy deposit 10 cm<br>Dispersed archeological material	NOT 17 08<br>ESR sample
5.3	H<br>(6 m²)	H1c	Silty-sandy deposit. Sandier and oxidized with a few micro-beds of dark minerals 30 cm<br>Dispersed archeological material	NOT 17 07<br>NOT 17 09<br>NOT 17 22<br>ESR sample
6.1	I<br>(26 m²)	I1a-b-c	Local lenses of small pebbles 15-30 cm<br>Coarse sands and beds of more or less dense gravels with pluri-millimetric anastomosed crusts 40-45 cm<br>Dispersed archeological material	NOT 17 10<br>NOT 17 11<br>NOT 17 12<br>NOT 17 13<br>Sample Ar
6.2	I<br>(26 m²)	I2	Dense accumulation of cobbles and smaller elements with limestones pebbles and a few fine-grained sandstone cobbles and flint nodules 10-15 cm<br>Dispersed archeological material	NOT 17 14
7.1			Tuffaceous sub-unit 3 cm	NOT 17 15
7.2			Coarse yellow sands with a few cobbles 15 cm	NOT 17 16<br>Sample Ar
7.3		J1	Tephra-derived coarse sands with some cobbles 10 cm	NOT 17 17<br>Sample Ar
7.4	J<br>(4 m²)	J2	Cobbles in a clayish volcano-derived matrix 30 cm	NOT 17 18<br>NOT 17 19
8.1			Light-grey sand and micro-breccia	NOT 17 20
8.2			Coarse yellow sands	NOT 17 21

Lithostratigraphy and sediment analysis

Jean-Paul Raynal (1, 2), Pauline Dugas (1), Gwenolé Jouanic (3), Alain Queffelec (1)

1) PACEA, UMR CNRS 5199, Université de Bordeaux, Bât B2 Allée Geoffroy St Hilaire, 33615 Pessac cedex (France)
2) Department of Human Evolution, Max Planck Institute for Evolutionary Anthropology, Leipzig (Germany)
3) Laboratoire Chrono Environnement, UMR CNRS 6249, Université de Bourgogne Franche Comté, 16 route de Gray, F 25030 Besançon cedex (France)

1 - Methods

We used classical field and laboratory methods. Names of formations and the definition of units follow international stratigraphic rules (Salvador, 1994). The description of sedimentary units, when possible, uses Miall's terminology for architecture and facies (1978, 1992, 2006), keeping in mind that the scale of the outcrops considerably limits interpretations. Volcanic and volcano-derived units were described based on Cas and Wright (1992).

Sedimentological samples were mostly taken loose for grain-size analysis and some in blocks for thin section investigation (J.-P. R.). They were processed for particle size analysis, geochemical analysis and thin section preparation at PACEA laboratory (University of Bordeaux) (P.D. and A.Q.).

A Horiba LA-950 laser particle size analyzer was used for grain size analysis of the twenty samples . Sample pretreatment included suspension in sodium hexametaphosphate (5 g/l) and hydrogen peroxide (35%) at room temperature for 12 h. Then, the suspension was subjected to 60 s ultrasounds to achieve optimal dispersion. The Mie solution to Maxwell's equation provided the basis for calculating particle size using a refractive index of 1.333 for water and 1.55i – 0.01i for particles (Sitzia et al. 2017). Grain size distribution expressed in ϕ units was broken down into different Gaussian populations (parametric curve fitting method) to identify and quantify the main modes, using the method proposed by Bosq et al. (2018). Results are presented in tables 1 and 2 and in several diagrams (**Fig. S1, S2 and S3**).

X-Ray fluorescence was used to characterize one sample after grinding and transformation into 300 mg pellets. X-ray fluorescence (ED-XRF) analysis was carried out using a portable SPECTRO X-SORT (40 kV, 50µA). Measurements were recorded in an air path with an acquisition time of 300 s. The device was calibrated beforehand using ICP-AES/ICP-MS compositions from 26 samples of Neogene and Quaternary sediments obtained by the SARM-CRPG laboratory in Nancy (Sitzia et al. 2018). Only elements for which a correlation coefficient (R2) greater than 0.9 between ED-XRF and ICP-AES/ICP-MS values were taken into account. Elements lighter than Si are not detected with this device.

Thin sections from three undisturbed sediment blocks were vacuum-impregnated with polyester resin and processed using the method described by Guilloré (1980).
The mineralogical content of thin sections was studied under the microscope in natural and polarized-analyzed light and minerals were identified by their optical characteristics (G. J.).

2 - Lithostratigraphy, sediments and dynamics

2.1 – General setting

The deposits excavated since 2016 belong to the *Notarchirico Complex* and are in geometrical continuity with the sequence established by former excavations: they consist of units 5 to 8 and archaeological layers H to J (with their subdivisions), identified below units 1 to 4, and archaeological layers alpha to G (Raynal *et al.,* 1999). In this complex, unit 3 is capped by the *Notarchirico Tephra complex* (Vernet *et al.,*1999; Lefèvre *et al.,* 2010*),* which was partly dated to 640 ± 70 Ma by TL (Pilleyre et al., 1999). More recently, units 3 to 1 have been dated from 670 ± 14 ka to 614 ± 12 ka by $^{40}Ar/^{39}Ar$ (Pereira *et al.,* 2015).

They lie above the *Piano Reggio Formation* identified in the Venosa Basin and found in 1988 in a core (Vn 88-2) extracted in the deposits underlying the earliest archaeological layer (H) (**Fig. S4**), about 50 m away from the main excavation in the northwest part of the site, at 354.5 m above sea level. A detailed lithostratigraphy of these deposits has been already published (Lefevre *et al.,* 1993, 1994, 1999, 2001, 2002, 2010; Raynal *et al*., 1998; Vernet *et al.,* 1999).

2.2 – Lithostratigraphy

The units were labelled in accordance with the previously established lithostratigraphy (Raynal *et al., op. cit*.), with numbers for the lithostratigraphic units and capital letters for the archaeological layers. They are described from the top to the bottom of the new excavations (unit 3 to unit 8).

Unit 3

Cross-bedded volcano-derived and non-volcanic sands, with huge variations in grain size distribution but generally coarse to very coarse: this is a clear equivalent of lithostratigraphic unit 3 identified in the site-museum. These deposits lay on an erosional undulated surface. Observed thickness is 20 cm. Sample 17-01 (taken 5 cm above the limit with Unit 4, 10 cm below the ESR sample) is representative (**Fig. S1**). Kurtosis and skewness (**table S1**) indicate a well-sorted sediment with a type 1 modal distribution (**Table 2, Fig. 2**), with a typical sigmoidal granulometric curve expressing the dominance of sand (about 84 %; Md = 674 microns) composition (**Fig. S3).** This is the coarsest sediment in the analyzed sequence. Fluvial processes (or at least heavy runoff) governed this sedimentation and volcaniclastics do not represent direct inputs.

Unit 4

Sub-unit 4.1

Sandy sub-unit, with a compact base. Observed thickness is 1 m. More or less coarse iron-coated sands (sample 17-03) characterize a volcano-derived sedimentary unit (**Fig. S1).** Kurtosis and skewness (**Table 1**) indicate a relatively well-sorted sediment with type 1 modal distribution (**Tables S2, S3, Fig. S2**), with a granulometric curve expressing dominant sand (about 78 %; Md = 535 microns) composition. The matrix is made of weathered volcanic glass, coarse melanocratic lavas with smoothed grains, millimetric to centimetric weathered pumice. This point towards a degraded distal pyroclastic flow. A few cobbles (up to 5 cm) are present. The bottom part (sample 17-02) is finer and richer in brownish-greenish silts and clays with encrusted pebbles. Kurtosis and skewness (**table S1)** indicate a poorly-sorted sediment with a slight predominance of silts (about 45 %; Md = 42 microns)

with type 3 modal distribution (**Fig. S2**), with five modes and a two-part granulometric curve (sands and silts), each of which expresses relatively good sorting **(Fig. S3**).

Sub-unit 4.2

Dark-grey volcano-derived sands, containing rounded quartz grains (5-6 mm). Observed thickness is 30 cm. At the base, cobbles (5-10 cm in diameter) form the archaeological layer G, which is not a single stone-line but a thick lag deposit covered by fluvial sands.

Sub-unit 4.3

Coarse sands with cobbles and sub-angulous gravels and microbedding underlined by heavy mineral laminas. Overall stratification is planar and sub-beds are barely graded, suggesting fluvial dynamics. The contact with the underlying deposit is undulated.
Most of this sub-unit (sample 17-04) (**Fig. S1**) displays kurtosis and skewness parameters, **table S2**), indicating a poorly-sorted sediment dominated by sand (about 79 %; Md = 278 microns), with type 2 modal distribution (**Fig. S2**), with four modes and a regular granulometric curve (Fig. 4). Coarse sands (sample 17-05) with smaller elements form the base of this sub-unit (Fig. 1). Kurtosis and skewness (**Table S2**) indicate a better sorted sediment dominated by sands (about 75 %; Md = 254 microns), with type 2 modal distribution (**Fig. S2**), with four well-expressed modes and a regular granulometric curve (**Fig. S3**).

Unit 5

On the basis of previous observations, this yellowish unit looks like a degraded distal pyroclastic flow. It has been sub-divided into three sub-units: 5.1, 5.2 and 5.3. ESR samples were taken in 5.2 and 5.3. Observed thickness is about 80 cm.
Sub-unit 5.1 (sample 17-06) is a silty-sandy deposit (**Fig. S1**). Kurtosis and skewness (**Table S2**) indicate a very poorly-sorted sediment dominated by silts (about 50%; Md = 38 microns) with type 3 modal distribution (**Fig. S2**), with three well expressed modes and an irregular granulometric curve (**Fig. S3).**
Sub-unit 5.2 is a silty-sandy deposit from which an ESR sample was taken. In the same position, sample 17-07 (**Fig. S1**) contains about 60 % of silts (Md = 29 microns) and kurtosis and skewness indicate very poor sorting (T**able S2**). It shows type 4 modal distribution (**Fig. S2**) with two dominant modes on fine sands and coarse silts and a mainly straight and moderately steep granulometric curve (**Fig. 3**). Towards the base of 5.2, sample 17-08 (**Fig. S1**) is slightly sandier (Md = 34 microns). Kurtosis and skewness indicate slightly better, although still poor sorting (table 1). It displays type 4 modal distribution (**Fig. S2**) with two well expressed modes and a straight granulometric curve. The bottom of 5.2 is laterally sandier and more oxidized with a few micro-beds of dark minerals and an undulated base. It contains a *Palaeoloxodon* humerus. This is archaeological layer H. A thin section (sample 17-22) was cut in the matrix underlying the humerus (**Fig. S5, n°1**). The sediment (**Fig. S5, n°2**) is mainly formed of pyroxenes with dominant clinopyroxenes. A few alkaline feldspars (sometimes large) are present along with a few amphiboles. Minerals are xenomorphic and heavily weathered, particularly the pyroxenes (**Fig. S5, n°3**), but they preserve their optical properties and can be easily identified. The minerals are embedded in a microlithic matrix of alkaline feldspars and plagioclases, quartz(?) and abundant pyroxene fragments (**Fig. S5, n°4**).
Sub-unit 5.3 (sample 17-09) is a silty-sandy deposit (Md = 50 microns) from which another ESR sample was taken. Kurtosis and skewness (table 1) indicate very poor sorting. It belongs to type 3 modal distribution (Fig. 2), with dominant populations of fine sands and coarse silts and a sub-straight granulometric curve (**Fig. S3**).

Unit 6

Sub-unit 6.1

Coarse greyish to greenish sands and beds of more or less dense gravels, sometimes concentrated in lenses or metric shallow pits; coarse elements sometimes dip strongly. At its base, cobbles appear in a sandy matrix. Observed thickness is 30 cm.
Sub-unit 6.1 is crossed by pluri-millimetric anastomosed crusts. In an E-W perspective, these crusts are sub-horizontal and generally respect the sand beds; but from band 37, they plunge to the south and cut into the sand beds. Crusts get thicker and form ripples in sands, which disappear at the point of contact with unit 6.2. These ripples and banded carbonaceous accumulations evoke post-depositional events related to vadose waters running SW in conformity with the present-day slope and following the location of present-day drainage.
Sub-unit 6.1 contains dispersed archaeological material and was sampled for 40Ar/39Ar dating.
At the top, (sample 17-10) the sediment is sandy-silty (**Fig. S1**), with a median of 121 microns. Kurtosis and skewness indicate moderately good sorting (**Table S1**). It belongs to type 5 modal distribution (**Fig. S2**), with a main mode on sands and a secondary one on silts outlining an irregular granulometric curve (**Fig. S3**).
Among the carbonated bands, the sands (samples 17-11 and 17-12) are fairly identical (**Fig. S1**), with medians of 112 and 132 microns respectively. Kurtosis and skewness indicate poor to moderate sorting respectively (**Table S1**). They also present a type 5 modal distribution **(Fig. S2),** with a similar granulometric curve (**Fig. S3)**.
At the bottom, the sandy matrix (Md = 99 microns) of a cobbled layer (sample 17-13) is slightly richer in silts (**Fig. S1**) but kurtosis and skewness indicate poorer sorting (T**able S1**). It still presents a type 5 modal distribution (**Fig. S2**), with three well expressed modes forming an irregular granulometric curve (**Fig. S3**).

Sub-unit 6.2

This is a lag deposit, 10 to 15 cm thick, consisting of a dense accumulation of cobbles and pebbles (1 to 10 cm). It contains archaeological material inserted in the "pavement": this is archaeological layer I. It mainly contains limestone pebbles and a few fine-grained sandstone cobbles and flint nodules. Among the coarse elements, the median diameter of the fine sandy-silty beige matrix (sample 17-14) is 63 microns (**Fig. S1**). Kurtosis and skewness indicate poor sorting (table 1), with a type 5 modal distribution (**Fig. S2**), with no coarse sands, an increase in well-sorted fine sands (about 50 %) and coarse silts (about 30 %). The granulometric curve is still irregular (**Fig. S3**). This lag deposit and the characters of the matrix could indicate a dynamic re-equilibrium following a volcanic input.

Unit 7

The general aspect of unit 7 is similar to that of the distal pyroclastic flows identified in other localities of the basin and which form the *Piano Reggio Formation*.

Sub-unit 7.1

The top of 7 is a tuffaceous sub-unit 7.1, pink in color, 2-3 cm thick, with some dark grains and crustified at the top. Under the microscope (**Fig. S6**), the sediment of sample 17-15 looks very well sorted and is mainly composed of pyroxenes (54%), feldspars (32%) and quartz (14%), in a microlithic matrix of amphiboles and clinopyroxenes; some green clinopyroxene grains are bigger. This sub-unit is probably a direct tephra fall (co-ignimbrite?) trapped in stagnant water and marks a discontinuity in the sedimentary process.

Sub-unit 7.2

Coarse yellow sands with rare cobbles, 15 cm thick, siltier towards the base. Sample 17-16 was taken above a sample for 40Ar/39Ar dating. Its composition is sandy-silty with a median of 75 microns (**Fig. S1**), with kurtosis and skewness indicating very poor sorting (**Table S2**). It belongs to modal distribution type 6 (**Fig. S2**), with better expressed modes on fine sands and coarse silts which both form about 75 % of the sediment. The granulometric curve shows better silt sorting (Fig. 3), which could represent a tephra input.

Sub-unit 7.3

Tephra-derived coarse sands with some cobbles, 10 cm thick. This sub-unit was sampled for 40Ar/39Ar dating. Sands (Md = 254 microns) form more than 76 % of sample 17-17 (**Fig. S1**), which was taken at base of the 40A/39Ar sample. Kurtosis and skewness indicate relatively poor sorting for this sediment (T**able S2**) which shows type 2 modal distribution (**Fig. S2**), with a regular parabolic granulometric curve (**Fig. S3).**

Sub-unit 7.4

Cobbles in a clayish volcano-derived matrix form archaeological layer J and overlie the crustified top of unit 8. Observed thickness is about 30 cm. The matrix (sample 17-19) is sandy (about 66 %; Md = 120 microns) (**Fig. S1**); kurtosis and skewness indicate relatively poor sorting (**Table S1**); it belongs to type 5 modal distribution (**Fig. S2**), with three well-expressed modes and a regular parabolic granulometric curve (**Fig. S3**). The bottom of sub-unit 7.4 (sample 17-18), in contact with the top of unit 8, is much siltier with a median of 53 microns; kurtosis and skewness indicate very poor sorting (**table S1**). Type 6 modal distribution with better sorting on very fine sands and coarse silts (**Fig. S2**). The granulometric curve is regular (**Fig. S3**).

Unit 8

The surface of this unit is a carbonated crust (39.5 % of carbonates) which overlies the grey sands of sub-unit 8.1 and changes into yellow coarse sands in sub-unit 8.2. The aspect and mineralogy of this unit clearly derive from a volcanic input.

Sub-unit 8.1

Sample 17-20 (**Fig. S1**) is a silty-sandy sediment (Md = 129 microns), with kurtosis and skewness indicating poor sorting (table 1). It belongs to type 5 modal distribution, with better sorting on sandy modes (**Fig. S2**). The granulometric curve is irregular with two better expressed populations of grains (**Fig. S3**).
Under the microscope, sub-unit 8.1 (sample 17-20a) shows rare amphibole phenocrystals (**Fig. S6, 1**) with ortho and clinopyroxenes (**Fig. S6, 2**) embedded in a microlithic matrix of quartz, feldspars and glass.

Sub-unit 8.2

Coarse yellow sands (sample 17-21) form this unit (**Fig. S1**). Sands represent nearly 65 % of the sediment (Md = 361 microns), kurtosis and skewness indicate moderately good sorting (**Table S2).** It belongs to modal distribution type 2 (**Fig. S2**), with relatively good sorting of the sandy populations and a partly sigmoidal granulometric curve **(Fig. S3).**

2.3 – Sedimentary dynamics

These new observations and analyses define the sedimentary context of units 8 to 3, which can be summarized as follows:

Unit 8: volcano-derived to volcanic sedimentation filling of a shallow channel.
Sub-unit 7.4: lag deposit resulting from slope destabilization after tephra input.
Sub-units 7.3 to 7.1: tephra input with direct fall at top.
Sub-unit 6.2: lag deposit resulting from slope destabilization after tephra input.
Sub-unit 6.1: volcano-derived spasmodic sedimentation in shallow channels.
Unit 5: redeposited volcanic weathered input by spasmodic sedimentation in shallow channels.
Sub-unit 4.3: fluvial sands.
Sub-unit 4.2: lag deposit.
Sub-unit 4.1: redeposited volcanic input in fluvial context.
Unit 3: fluvial sands.

From an environment submitted to regular tephra inputs (units 8 to 6), sedimentation progressively passes from low to somewhat higher energy currents (units 5 to 3) but remains largely volcano-derived. The periods of spasmodic sedimentation and those following lag deposits and their elutriation are conducive to hominin and provide direct access to cobble materials.

This nevertheless confirms most of the conclusions previously established concerning the *Notarchirico Complex* (Raynal *et al.,* 1999). This set of deposits is not very thick and without doubt represents a relatively short period of accumulation. The different identified facies correspond mainly to fills of meandering paleo-channels, sometimes crossed as a result of the action of low-energy currents. Flow directions are difficult to establish from the rather small surfaces exposed in the new excavations. Moreover, the original slopes of the deposits have undoubtedly been modified by tectonic activity since the Early Pleistocene. The phasing of accumulation was largely dependent on the periodic emission of tephra originating mainly from the Monte Vulture Massif. These have usually been reworked over the site. The fine fractions are derived from the alteration facies of the tephra materials and participate in the multi-modal characters of the sediments as expressed by our analysis. The layers incorporating cobbles and gravels are the result of slope destabilization processes which intervened systematically after the arrival of the tephra masses and the release of lateral contributions from older conglomeratic deposits by dynamic events which remain to be determined (debris flows, mud flows…). These deposits have been saturated by water at maximum flow rates resulting in scouring, elutriation and the consequent concentration of coarse materials which form polygenic units and sometimes surfaces representing diastems. The duration of the latter have yet to be ascertained.

References

Bosq, M., Bertran, P., Degeai, J.-P., Kreutzer, S., Queffelec, A., Moine, O., Morin, E., 2018. Last Glacial aeolian landforms and deposits in the Rhône Valley (SE France): Spatial distribution and grain-size characterization. Geomorphology. https://doi.org/10.1016/j.geomorph.2018.06.010

Cas, R.A.F. and Wright, J. V., 1992. Volcanic successions. Modern and ancient. Chapman & Hall, London, 528 p.

Guilloré, P., 1980. Méthode de fabrication mécanique et en série des lames minces. Institut National d'Agronomie, Paris-Grignon (22 pp.).

Lefèvre, D., Raynal, J.P., Pilleyre, T., Vernet, G., 1993. Contribution à la chronostratigraphie de la série de Venosa-Notarchirico (Basilicata). In: Atti Della XXX Riunione Scientifica, Paleosuperfici del Pleistocene e del Primo Olocene in Italia. Processi fi Formazione e Interpretazione. Istituto Italiano di Preistoria e Protostoria, Firenze, pp. 117-128.
Lefèvre, D., Raynal, J.P., Vernet, G., Pilleyre, Th, Piperno, M., Sanzelle, S., Fain, S., Miallier, D., Montret, M., 1994. Sédimentation, volcanisme et présence humaine dans le bassin de Venosa (Basilicata, Italie) an Pléistocéne moyen: exemple du site di Notarchirico. Bulletin de la Société Préhistorique française 91 (2), 103-112.

Lefèvre, D., Raynal, J.-P. and Vernet, G., 1999. Enregistrements pléistocènes dans la bassin de Venosa. In M. Piperno (dir.) Notarchirico. Un sito des Pleistocene medio iniziale nel bacino di Venosa, Osanna Ed., Venosa, Museo « L. Pigorini », Roma, 139-173.

Lefèvre, D., Raynal, J.P., Vernet, G., Kieffer, G., Piperno, M., Tagliacozzo, A., 2001. Hommes et volcans au Pléistocène moyen dans le bassin de Venosa (Basilicata, Italie). In: Juvigné, E., Raynal, J.P. (Eds.), Tephras, chronology, archaeology, Les dossiers de l'Archéo-Logis no 1, CDERAD éd, pp. 175-182.

Lefèvre, D., Raynal, J.P., Vernet, G., Piperno, M., 2002. Volcanisme et premiers peuplements en Italie méridionale: l'exemple de Notarchirico (Venosa, Italie). In: Raynal, J.P., Albore-Livadie, C., Piperno, M. (Eds.), Hommes et volcans, de l'éruption à l'objet, Les dossiers de l'Archéo-Logis no 2, CDERAD éd, pp. 67-76.

Lefèvre, D., Raynal, J.-P., Vernet, G., Kieffer, G. and Piperno, M., 2010. Tephro-stratigraphy and the age of ancient Southern Italian Acheulean settlements: The sites of Loreto and Notarchirico (Venosa, Basilicata, Italy). Quaternary International, 223-224, 360-368.

Macdonald, P., Du, J., 2012. Mixdist: finite mixture distribution models. R package version 0.5-4. https://CRAN.R-project.org/package=mixdist.

Miall, A.D. (Ed.), 1978. Fluvial sedimentology. Canadian Society of Petroleum Geologists, 5, Calgary.

Miall, A.D. (Ed.), 1992. Alluvial deposits. In Walker R.G., James N.P. (eds), Facies Models: response to sea level change, Geological Association of Canada, St John's, New-foundland, 119-142.

Miall, A.D., 2006. The Geology of Fluvial Deposits. Sedimentary Facies, Basin Analysis, and Petroleum Geology. Springer, 582 p.

Pereira, A., Nomade, S., Voinchet, P., Bahain, J.-J., Falguères, C., Garon, H., Lefèvre, D., Raynal, J.-P., Scao, V. and Piperno, M., 2015. The earliest securely dated hominin fossil in Italy and evidence of Acheulian occupation during glacial MIS 16 at Notarchirico (Venosa, Basilicata, Italy). Journal of Quaternary Science, 30 (7), 639-650.

Pilleyre, T., Sanzelle, S., Fain, J., Miallier, D., Montret, M., 1999. Essai de datation par thermoluminescence des dépôts du site acheuléen de Notarchirico. In M. Piperno (dir.) Notarchirico. Un sito des Pleistocene medio iniziale nel bacino di Venosa, Osanna Ed., Venosa, Museo « L. Pigorini », Roma, 235-243.

Raynal, J.P., Lefevre, D., Vernet, G., Pilleyre, T., Sanzelle, S., Fain, J., Miallier, D., Montret, M., 1998. Sedimentary dynamics and tecto-volcanism in the Venosa Basin (Basilicata, Italia). Quaternary International 47/48, 97-105.

Raynal, J.-P., Lefèvre, D., Vernet, G. and Papy, G., 1999. Lithostratigraphie du site acheuléen de Notarchirico. In M. Piperno (dir.) Notarchirico. Un sito des Pleistocene medio iniziale nel bacino di Venosa, Osanna Ed., Venosa, Museo « L. Pigorini », Roma, 175-205.

R Core Team, 2018. R: A Language and Environment for Statistical Computing. Vienna,Austria. URL: https://r-project.org.

Salvador, A., 1994. International Stratigraphic Guide. A Guide to Stratigraphic Classification, Terminology and Procedure. Geological Society of America, 214 p.

Schulte, P., Lehmkuhl, F., Steininger, F., Loibl, D., Lockot, G., Protze, J., Fischer, P., Stauch, G., 2016. Influence of HCl pretreatment and organo-mineral complexes on laser diffraction measurement of loess–paleosol-sequences. Catena 137, 392–405.

Sitzia, L., Bertran, P., Sima, A., Chery, P., Queffelec, A., Rousseau, D.-D., 2017. Dynamics and sources of last glacial aeolian deposition in southwest France derived from dune patterns, grain-size gradients and geochemistry, and reconstruction of efficient wind directions. Quaternary Science Reviews 170, 250–268. https://doi.org/10.1016/j.quascirev.2017.06.029

Sitzia, L., Gayo, E.M., Sepulveda, M., González, J.S., Ibañez, L., Queffelec, A., Pol-Holz, R.D., 2019. A perched, high-elevation wetland complex in the Atacama Desert (northern Chile) and its implications for past human settlement. Quaternary Research 1–20. https://doi.org/10.1017/qua.2018.144

Vernet, G., Raynal, J.P., Lefevre, D., Kieffer, G., 1999. Un bassin, un volcan: téphras distales dans les dépôts du Pléistocène moyen de Venosa (Basilicata, Italia). In M. Piperno (dir.) Notarchirico. Un sito des Pleistocene medio iniziale nel bacino di Venosa, Osanna Ed., Venosa, Museo « L. Pigorini », Roma, 207-233.

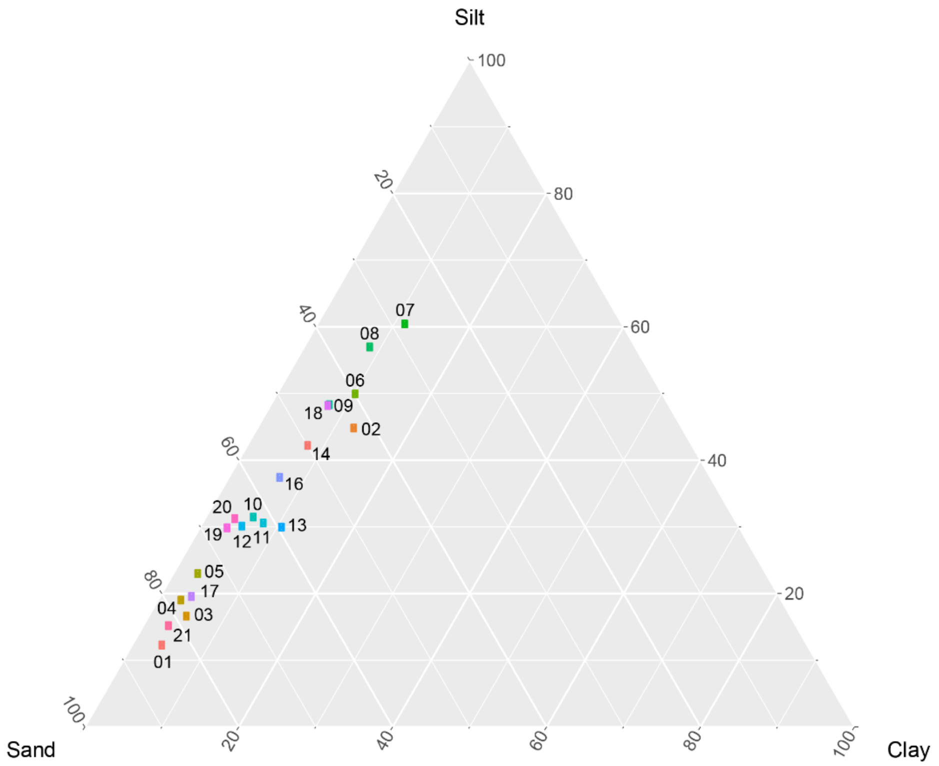

Fig. S1. Triangular textural diagram.

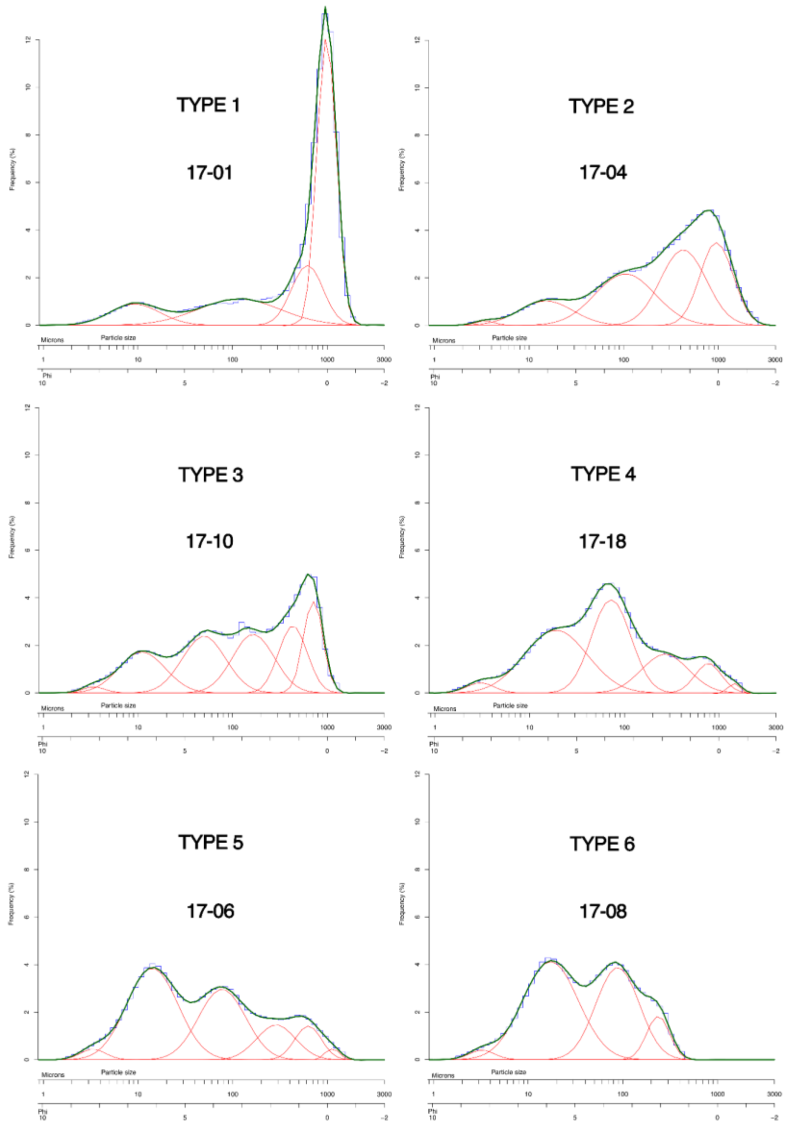

Fig. S2. The six different types of modal distribution representative of the sedimentary units.

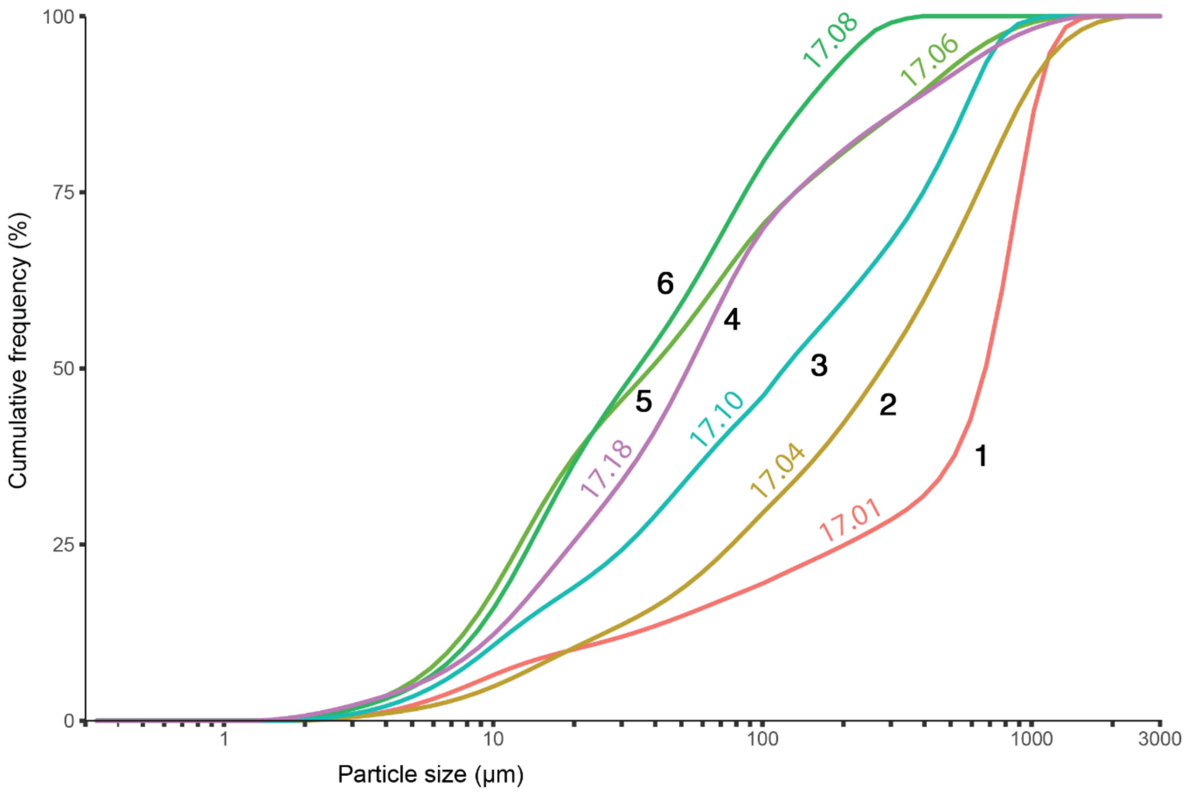

Fig. S3. The six different types of granulometric curves representative of the sedimentary units.

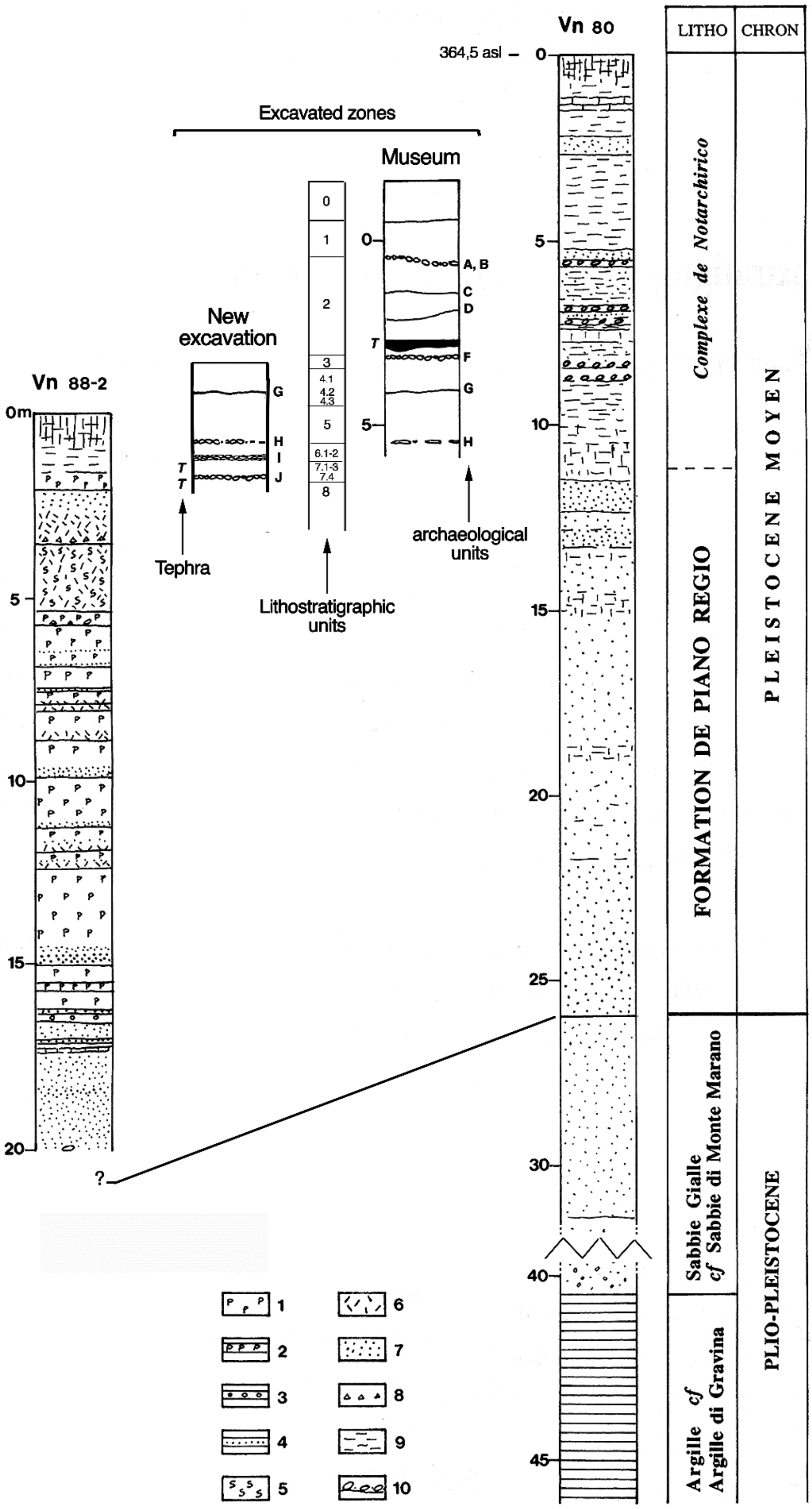

Fig. S4. Global stratigraphy at the archaeological site of Notarchirico: the Notarchirico Complex excavated in two zones and lying in stratigraphic continuity above the Piano Reggio Formation. Legend for the cores: 1; ignimbrite with pumice; 2, pumice Plinian fall; 3, vesiculated tuff; 4, cinerite; 5, scoria fall; 6, ignimbrite base rich in free crystals; 7, sands; 8, angulous macro-clasts; 9, silts; 10, bed of cobbles and pebbles (modified from Raynal et al.,1999).

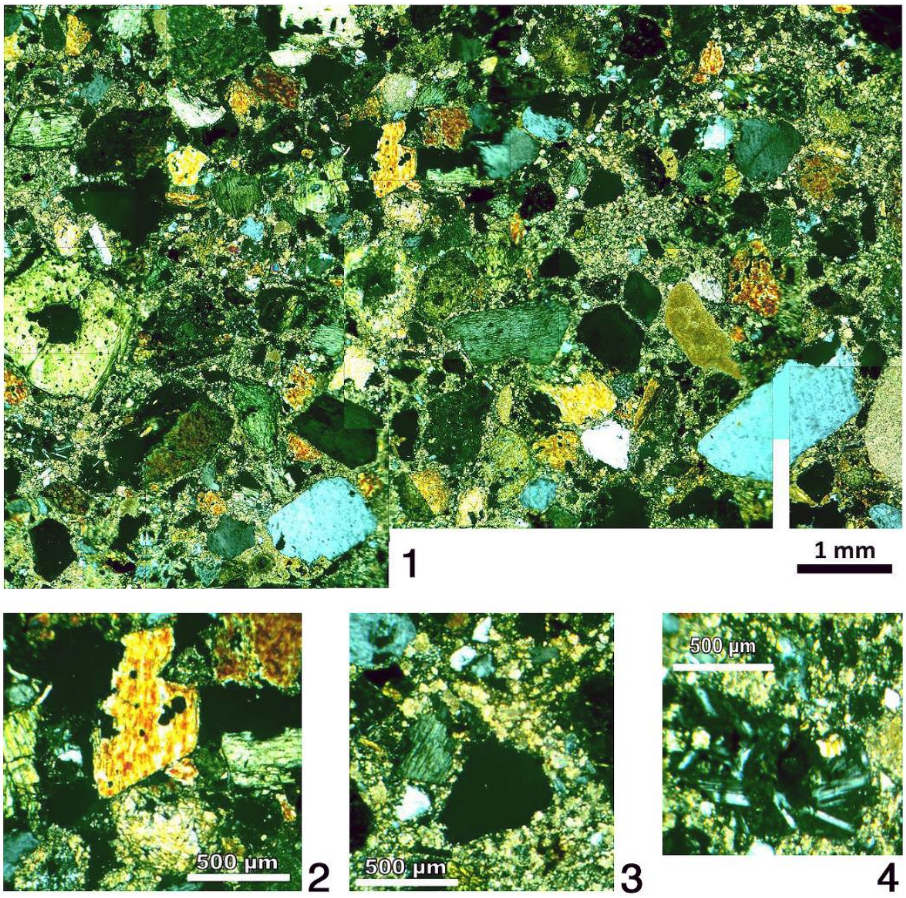

Fig. S5. Sub-unit 5.2, sample 17-22, thin section, polarized and analyzed light. 1, general view of the matrix. 2, alteration of clinopyroxene. 3, detail of matrix. 4, plagioclase microliths.

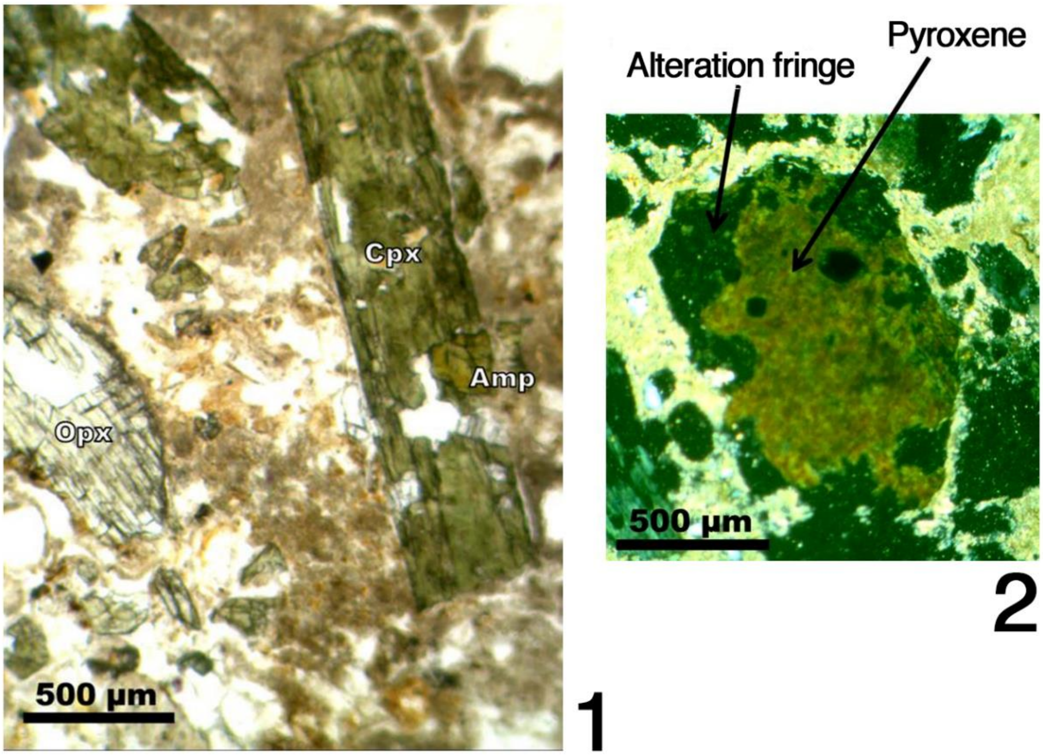

Fig. S6. Sub-unit 8.1, thin section in natural light (1) and polarized and analyzed light (2).

Table S2. Main granulometric characteristics of the analyzed samples presented in stratigraphic order.

Stratigraphic units		Sample	P10 microns	P25 microns	Md microns	P75 microns	P90 microns	% coarse sands (2000-500µ)	% fine sands (500-63µ)	% coarse silts (63-16µ)	% fine silts (16-7µ)	% clays (<7µ)	Kurtosis	Skewness
3	3	Not 17 01	19.37	202.61	673.81	897.30	1078.79	63.24	20.43	7.17	5.11	4.05	1.99	0,10
4	4.1 top	Not 17 03	12.88	91.45	534.66	871.02	1176.61	51.68	26.64	9.23	7.39	5.07	2.91	0.65
	4.1 base	Not 17 02	6.01	11.65	42.11	183.11	519.25	10.66	31.81	24.30	20.52	12.72	5.92	1.90
	4.3	Not 17 04	19.11	77.06	277.93	630.96	984.83	32.65	45.17	13.30	5.72	3.16	4.63	1.32
	4.3 base	Not 17 05	14.92	57.66	254.28	621.28	959.57	32.28	41.37	15.59	7.40	3.36	3.92	1.18
5	5.1	Not 17 06	6.87	12.56	38.49	132.74	414.64	7.43	32.30	28.33	21.60	10.34	9.90	2.55
	5.2	Not 17 07	6.34	12.31	28.93	70.45	184.54	0.72	27.31	38.85	21.57	11.55	9.75	2.62
	5.2 base	Not 17 08	7.58	13.70	34.31	86.33	163.35	0.00	34.31	35.80	21.17	8.72	5.86	1.73
	5.3	Not 17 09	8.16	16.28	50.17	129.51	306.64	4.35	39.53	31.54	16.77	7.80	10.14	2.55
6	6.1	Not 17 10	9.51	31.49	120.75	395.74	616.79	17.56	44.65	21.32	10.14	6.34	3.22	1.08
	6.1	Not 17 11	8.24	29.79	112.16	342.15	550.61	13.00	48.35	20.68	9.88	8.09	3.91	1.25
	6.1	Not 17 12	11.00	37.11	132.00	440.66	651.24	20.22	44.13	21.78	8.32	5.54	3.33	1.06
	6.1	Not 17 13	6.60	23.42	98.93	358.07	559.04	13.71	45.57	19.16	10.80	10.77	3.65	1.18
	6.2	Not 17 14	8.42	20.64	62.92	120.42	207.80	0.00	49.95	29.22	13.14	7.69	4.79	1.41
7	7.2	Not 17 16	9.29	26.08	74.54	181.27	408.34	7.20	48.59	26.14	11.27	6.80	9.96	2.48
	7.3	Not 17 17	15.27	69.85	253.85	632.90	1031.11	31.72	44.46	13.01	6.54	4.26	4.53	1.36
	7.4	Not 17 19	13.64	43.22	119.91	262.33	419.81	5.79	60.61	22.07	7.78	3.75	4.41	1.31
	7.4	Not 17 18	8.57	19.56	53.02	132.38	436.17	8.48	35.67	34.92	13.28	7.65	11.49	2.82
8	8.1	Not 17 20	13.95	39.98	129.47	419.81	658.54	19.40	45.32	23.97	7.26	4.06	3.67	1.19
	8.2	Not 17 21	24.53	102.00	361.50	824.66	1204.76	40.93	40.43	10.29	4.90	3.46	3.82	1.09

Table S3. Grain-size distribution of the analyzed samples broken down into Gaussian populations presented in stratigraphic order.

Sample	Match	Proportion	Mode microns	Mode phi	Sigma
Not.17.01	0.78	52.3	989.61	0.02	0.33
		17.7	616.18	0.7	0.54
		20.1	117.31	3.09	1.42
		9.9	9.29	6.75	0.87
Not.17.03	0.88	51.4	971.59	0.04	0.53
		17.2	420.88	1.25	0.77
		14.6	96.62	3.37	0.83
		16.8	12.36	6.34	1.05
Not.17.02	0.61	10.6	729.72	0.45	0.37
		13.8	358.97	1.48	0.63
		32	79.94	3.64	0.93
		39.7	12.71	6.3	0.86
		3.9	3.29	8.25	0.5
Not.17.04	0.62	25.7	959.15	0.06	0.57
		32.3	426.68	1.23	0.78
		29	103.59	3.27	1.04
		12.3	15.08	6.05	0.93
		0.7	3.42	8.19	0.33
Not.17.05	1.33	12.9	1062.67	-0.09	0.45
		40.3	545.72	0.87	0.79
		23.1	121.98	3.04	0.83
		0	54.86	4.19	0.99
		23.1	21.82	5.52	1.21
		0.6	4.15	7.91	0.36
Not.17.06	0.44	1.4	1126.69	-0.17	0.23
		8.4	624.82	0.68	0.46
		12.8	287.36	1.8	0.67
		31	77.2	3.7	0.8
		43.9	14.13	6.15	0.88
		2.5	3.33	8.23	0.44
Not.17.07	0.53	4.7	463.63	1.11	0.25
		6	267.81	1.9	0.47

Stratigraphic unit	Sample	Match	Proportion	Mode microns	Mode phi	Sigma
6.1	Not.17.11	0.49	20.8	595.51	0.75	0.44
			23.5	290.21	1.78	0.65
			3.7	133.99	2.9	0.26
			32.7	62.06	4.01	0.95
			17.3	9.98	6.65	0.81
			2	3.15	8.31	0.42
6.1	Not.17.12	0.42	24.4	674.52	0.57	0.43
			18.8	388.01	1.37	0.57
			13.2	147.82	2.76	0.54
			27.3	54.9	4.19	0.79
			15.2	11.31	6.47	0.86
			1.3	3.17	8.3	0.43
6.1	Not.17.13	0.53	22.4	593.21	0.75	0.44
			19.6	312.73	1.68	0.6
			34.2	72.41	3.79	0.92
			20.4	9.79	6.68	0.82
			3.1	2.98	8.39	0.53
			0.3	0.32	11.62	0.14
6.2	Not.17.14	0.65	14.3	256.61	1.96	0.42
			45.7	96.65	3.37	0.7
			7.8	55.66	4.17	0.63
			29.9	14.94	6.06	0.97
			2.3	3.14	8.31	0.42
7.2	Not.17.16	0.49	1	1208.42	-0.27	0.18
			6.3	695.87	0.52	0.43
			22.8	275.64	1.86	0.71
			40.7	83.94	3.57	0.71
			27.2	15.58	6	1.02
			2	3.1	8.33	0.42
7.3	Not.17.17	1	22.8	1050.74	-0.07	0.54
			35.8	414.74	1.27	0.83
			0	232.39	2.11	1.08

		35.9	69.14	3.85	0.8
		49.3	16.02	5.96	0.95
		4.2	3.2	8.29	0.49
Not.17.08	0.56	9.3	235.34	2.09	0.39
		37.4	88.13	3.5	0.74
		51.1	17.2	5.86	0.96
		2.2	3.24	8.27	0.44
Not.17.09	0.65	4	704.03	0.51	0.36
		14.3	310.84	1.69	0.65
		40.5	86.75	3.53	0.85
		39	16.39	5.93	0.95
		2.3	3.25	8.27	0.45
Not.17.10	0.48	16.9	707.3	0.5	0.34
		17.9	433.55	1.21	0.49
		23	165.69	2.59	0.72
		23.1	49.89	4.33	0.75
		17.8	11.04	6.5	0.8
		1.3	3.4	8.2	0.38

			25	103.21	3.28	0.9
			7.1	21.34	5.55	0.9
			9.3	10.03	6.64	1.04
7.4	Not.17.19	0.7	13	501.75	0.99	0.44
			30.6	257.48	1.96	0.65
			44.4	74.23	3.75	1.07
			11.2	11.06	6.5	0.72
			0.7	3.89	8.01	0.3
7.4	Not.17.18	0.57	1	1431.1	-0.52	0.16
			7.4	771.2	0.37	0.47
			17.1	269.22	1.89	0.81
			34.4	72.6	3.78	0.67
			37.5	18.82	5.73	1.1
			2.7	3	8.38	0.48
8.1	Not.17.20	0.47	14.6	771.69	0.37	0.39
			19.5	488.21	1.03	0.51
			26.3	170.58	2.55	0.82
			25.7	50.28	4.31	0.73
			13	12.22	6.35	0.85
			0.9	3.32	8.23	0.38

40Ar/39Ar analytical data

Alison Pereira (1, 2), Sébastien Nomade (3)
1) Département Homme et Environnement, UMR 7194, HNHP, Muséum National d'Histoire Naturelle, Paris, France.
2) Ecole Française de Rome, Italia
3) CEA Saclay, LSCE, Gif-sur-Yvette, France.

Table. S4. NOT I1 (sub-unit 6.1) 40Ar/39Ar analytical data

Sample ID: NOT I1 sub-unit 6.1 — Lab#N1571-01/N1571-15 — Sanidines

Irradiation # CO-001 — $J = 0.00052230 \pm 0.00000209$ — reactor: CICLIT, Oregon University, Triga reactor

Flux standard monitor: ACs-2, 1.1891 Ma — Single crystal total fusion — duration: 2 hours

N	^{40}Ar (moles)	^{36}Ar V	$\pm\sigma_{36}$ V	^{37}Ar V	$\pm\sigma_{37}$ V	^{38}Ar V	$\pm\sigma_{38}$ V	^{39}Ar V	$\pm\sigma_{39}$ V	^{40}Ar V	$\pm\sigma_{40}$ V	$D^{(1)}$	$\pm\%\sigma_D$	$\%^{40}Ar^*$	Age (ka)	±σ (ka)	K/Ca	±σ
N1571-01	5.145E-15	2.15E-07	7.453	6.03E-05	0.701	6.16E-05	0.264	5.02E-03	0.116	0.004	0.101	1.006	0.10	97.91	691.1	± 1.4	35.8	± 0.3
N1571-02	7.163E-15	3.35E-07	5.317	1.21E-04	0.717	8.52E-05	0.378	7.01E-03	0.116	0.005	0.060	1.007	0.10	97.76	688.2	± 1.2	24.9	± 0.2
N1571-03	5.069E-15	6.50E-07	3.993	5.99E-05	0.826	5.68E-05	0.405	4.76E-03	0.117	0.004	0.097	1.006	0.10	94.39	693.3	± 1.9	34.2	± 0.3
N1571-04	6.115E-15	2.61E-07	4.610	1.01E-04	0.681	6.99E-05	0.439	5.86E-03	0.116	0.004	0.101	1.006	0.10	97.94	704.5	± 1.2	24.9	± 0.2
N1571-05	3.286E-15	1.49E-07	12.055	8.40E-05	1.659	3.62E-05	0.451	3.04E-03	0.122	0.002	0.093	1.006	0.10	97.94	729.8	± 2.0	15.6	± 0.3
N1571-06	6.761E-15	3.02E-07	5.138	8.72E-05	1.095	7.52E-05	0.362	6.19E-03	0.134	0.005	0.091	1.007	0.10	97.84	736.7	± 1.4	30.5	± 0.3
N1571-07	4.014E-15	4.52E-08	32.920	4.00E-05	1.739	4.82E-05	0.513	3.97E-03	0.122	0.003	0.111	1.006	0.10	99.13	690.6	± 1.6	42.7	± 0.7
N1571-08	6.801E-15	3.55E-07	5.007	1.66E-04	0.620	7.35E-05	0.362	6.08E-03	0.116	0.005	0.101	1.007	0.10	97.66	752.5	± 1.4	15.8	± 0.1
N1571-09	3.781E-15	1.99E-07	7.780	4.27E-05	1.530	4.23E-05	0.308	3.51E-03	0.116	0.003	0.062	1.006	0.10	97.48	723.7	± 1.6	35.4	± 0.5
N1571-10	2.617E-15	7.28E-07	2.499	5.00E-05	1.651	2.64E-05	0.665	2.21E-03	0.128	0.002	0.126	1.006	0.10	88.38	721.9	± 2.7	19.0	± 0.3
N1571-11	4.884E-15	4.46E-07	4.411	6.14E-05	1.418	5.39E-05	0.558	4.48E-03	0.197	0.004	0.212	1.006	0.10	95.92	720.8	± 2.5	31.4	± 0.4
N1571-12	2.689E-15	1.49E-07	13.111	5.95E-05	1.424	3.14E-05	0.450	2.61E-03	0.122	0.002	0.185	1.006	0.10	97.47	692.4	± 2.6	18.9	± 0.3
N1571-13	4.009E-15	4.38E-07	5.447	5.30E-05	1.523	4.59E-05	0.539	3.80E-03	0.122	0.003	0.097	1.006	0.10	95.18	692.8	± 2.1	30.8	± 0.5
N1571-14	3.223E-15	1.89E-08	109.981	5.07E-05	0.657	3.87E-05	0.283	3.22E-03	0.122	0.002	0.110	1.006	0.10	99.41	686.8	± 2.1	27.3	± 0.2
N1571-15	6.528E-15	8.94E-08	25.011	1.13E-04	1.129	7.59E-05	0.305	6.18E-03	0.116	0.005	0.071	1.007	0.10	99.13	721.3	± 1.4	23.6	± 0.3

Results	$^{40}Ar^*/^{39}Ark$	±σ	Age (Ka)	±σ	MSWD	$^{39}Ar(k)$ (%, n)	K/Ca	±σ
Weighted mean	0.7311	± 0.0009	690.3	± 2.9	1.83	44.73	27.9	± 2.2
		± 0.12%		± 0.42%		7		
		Full External Error		± 3.2	1.01	Statistical T ratio		
		Analytical Error		± 0.9	1.3538			

Results	40(a)/36(a)	±σ	40(r)/39(k)	±σ	Age (Ka)	±σ	MSWD
Inverse Isochron	337.82	± 18.4	0.7291	± 0.0012	688.4	± 3.0	1.05
		± 5.44%		± 0.16%		± 0.43%	
Full External Error						± 3.2	
Analytical Error						± 1.1	

Statistics				
	Statistical F ratio	1.11	Convergence:	0.0001800283
	Error Magnification	1.0264	Number of Iterations	3
	Number of Data Points	7	Calculated Line ted York-2	

Background corrections NOTI1/sub-unit 6.1

N	^{36}Ar V	±σ V	^{37}Ar V	±σ V	^{38}Ar V	±σ V	^{39}Ar V	±σ V	^{40}Ar V	±σ V
N1571-01	2.078E-07	1.455E-08	2.790E-07	2.343E-08	4.778E-08	1.949E-08	1.690E-07	1.572E-07	1.733E-05	2.773E-07
N1571-02	2.078E-07	1.455E-08	2.790E-07	2.343E-08	4.778E-08	1.949E-08	1.690E-07	1.572E-07	1.733E-05	2.773E-07
N1571-03	2.078E-07	1.455E-08	2.790E-07	2.343E-08	4.778E-08	1.949E-08	1.690E-07	1.572E-07	1.733E-05	2.773E-07
N1571-04	2.117E-07	1.059E-08	3.016E-07	1.659E-08	5.006E-08	1.299E-08	4.451E-07	1.335E-07	1.866E-05	4.478E-07
N1571-05	2.213E-07	1.306E-08	2.551E-07	2.398E-08	7.106E-08	1.926E-08	1.251E-07	1.021E-07	1.866E-05	4.478E-07
N1571-06	2.213E-07	1.306E-08	2.551E-07	2.398E-08	7.106E-08	1.926E-08	1.251E-07	1.021E-07	1.597E-05	3.993E-07
N1571-07	2.213E-07	1.306E-08	2.551E-07	2.398E-08	7.106E-08	1.926E-08	1.251E-07	1.021E-07	1.597E-05	3.993E-07
N1571-08	2.213E-07	1.306E-08	2.551E-07	2.398E-08	7.106E-08	1.926E-08	1.251E-07	1.021E-07	1.597E-05	3.993E-07
N1571-09	2.213E-07	1.306E-08	2.551E-07	2.398E-08	7.106E-08	1.926E-08	1.251E-07	1.021E-07	1.597E-05	3.993E-07
N1571-10	1.993E-07	1.515E-08	2.118E-07	1.821E-08	9.074E-08	1.279E-08	2.906E-07	1.258E-07	3.040E-05	5.989E-07
N1571-11	1.993E-07	1.515E-08	2.118E-07	1.821E-08	9.074E-08	1.279E-08	2.906E-07	1.258E-07	3.040E-05	5.989E-07
N1571-12	1.993E-07	1.515E-08	2.118E-07	1.821E-08	9.074E-08	1.279E-08	2.906E-07	1.258E-07	3.040E-05	5.989E-07
N1571-13	1.952E-07	1.952E-08	2.667E-07	1.334E-08	1.143E-08	2.195E-09	1.895E-07	1.622E-07	2.600E-05	5.200E-07
N1571-14	1.952E-07	1.952E-08	2.667E-07	1.334E-08	1.143E-08	2.195E-09	1.895E-07	1.622E-07	2.600E-05	5.200E-07
N1571-15	1.952E-07	1.952E-08	2.667E-07	1.334E-08	1.143E-08	2.195E-09	1.895E-07	1.622E-07	2.600E-05	5.200E-07

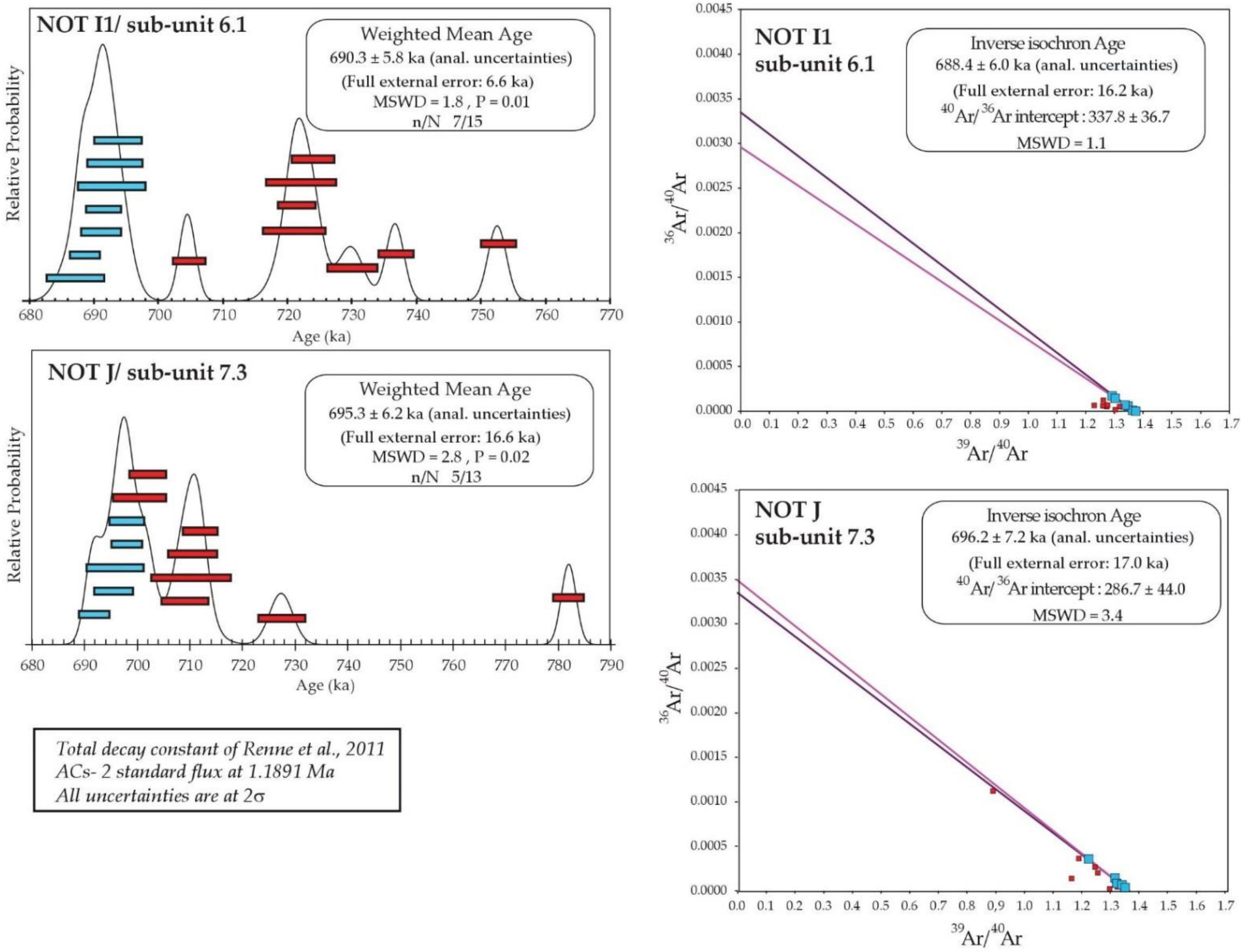

Figure S7. Probability diagrams and inverse isochrons showing the 40Ar/39Ar results for sedimentary sub-units 6.1 and 7.3.

Vertebrate Paleontology

Beniamino Mecozzi, Alessio Iannucci, Raffaele Sardella

Dipartimento di Scienze della Terra, Sapienza Università di Roma, Italia

The early Middle Pleistocene is characterized by major reorganizations of terrestrial ecosystems, occurring in successive phases during paleoenvironmental changes, strongly influenced by the onset of 100 ka climate cyclicity. Dispersal phases, mainly related to the progressive diffusion in Italy of taxa from Eastern and Central Europe, led to mammal faunal renewal. The biochronology of the early Middle Pleistocene vertebrate faunas is related to the Galerian Mammal Age, which includes Ponte Galeria, Isernia and Fontana Ranuccio Faunal Units (Gliozzi et al., 1997; Sardella et al. 2006; Bellucci et al 2015).
The faunal assemblage from Venosa has been studied and published by different authors who focused their analyses on fossils from the Loreto site (Caloi and Palombo 1979a, b, Alberdi et al 1988). The vertebrate remains from Notarchirico (upper levels A and Alfa) were studied by Cassoli et al. (1999) (large mammals), and Sala (1999) (micromammals). Together with some turtles and birds, the following mammals have been identified: *Lepus* cf. *L. europaeus*, Elephantidae, *Palaeoloxodon antiquus*, *Sus scrofa*, Cervidae indet., *Dama clactoniana*,? *Axis* sp., *Cervus elaphus*, *Praemegaceros* sp., Bovidae indet., *Bos primigenius*, *Bison* sp. Cassoli et al. (1999) underlined the possible occurrence of *Axis* and *C. elaphus eostephanoceros* in the Cervidae family, and the contemporaneous presence of *Bos* and *Bison*. *Bison schoetensacki* was recorded in level D, underlying levels A and Alfa. The Notarchirico Local Fauna has been ascribed to the Isernia FU.

Materials and discussion

Fossils come from layers F, G, H, J, I1 and I2. Out of a NR of 4081, 750 remains have been studied.

The fossil remains are fragmented. Nevertheless, some specimens have been attributed to several mammal species (**Table S5).** The straight-tusked elephant (*P. antiquus*) was recovered from all stratigraphic levels. The study of the ungulate found in 2017 is in progress and confirms the occurrence of cervids and bovids. A more detailed taxonomic attribution is complicated by the fragmentary nature of the fossils, generally limb bone shaft fragments, possibly related to megacerine deer or slender bovids. Among bovids, the occurrence of bison and aurochs is documented, although the remains referred to *Bos primigenius* are not fully diagnostic, as they are mainly based on metapodials. The occurrence of a bubaline bovid, *Hemibos galerianus* (recorded at Casal Selce, Roma, in a stratigraphic level estimated at 0.7 ky) (Sardella 2007) cannot be ruled out.
Among the cervids, *Praemegaceros solilhacus* is relatively abundant, while red deer is rare. No carnivore fossils or small mammals have been found. The occurrence of *Macaca sylvanus* spp. is documented by a proximal half of a right ulna **(Fig. S8).** The presence of the Barbary macaque is of particular interest, as the specimen from Venosa Notarchirico is the southernmost evidence of this species in Italy.
Since the Middle Pleistocene, the faunal composition of the sites is strongly and increasingly affected by human activity. Faunal lists are therefore greatly influenced by the activity of humans selecting prey and “keeping” predators away. Therefore, information from Middle Pleistocene terrestrial ecosystems has to be “filtered” by an accurate evaluation of human impact.

Table S5. Mammal species from the layer F-I2

Layer / Species	F	G	I1	I2
Palaeoloxodon antiquus	X	X	X	X
Hippopothamus antiquus		X	X	
Bison schoetensacki	X	X	X	X
Bovidae indet.	X		X	X
Praemegaceros solilhacus			X	X
Cervus elaphus			X	X
Macaca Sylvanus spp.		X		

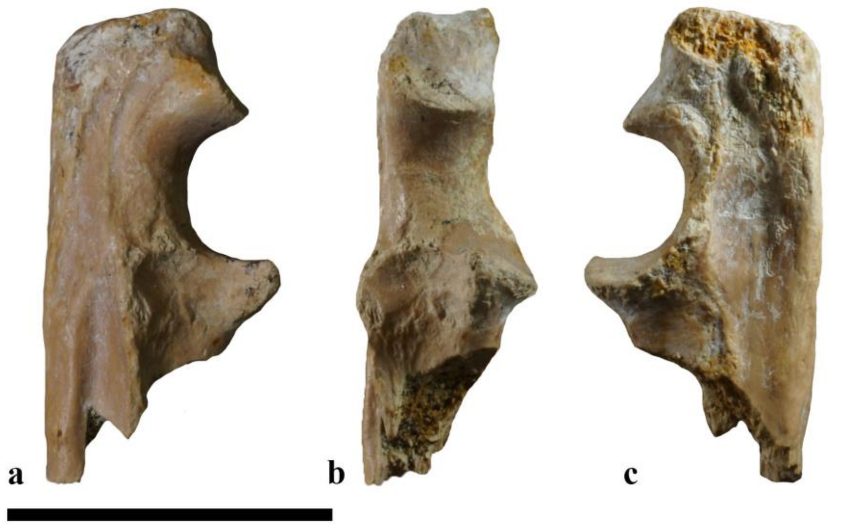

Figure S8. B24GNC, fragmented right ulna in medial, anterior and lateral views of *Macaca Sylvanus* spp. (photos B.Mecozzi, A. Iannucci, R. Sardella)

References

Alberdi, M.T., Caloi, L., Palombo, M.R. 1988. The Quaternary fauna of Venosa: equids. Bulletin du Musée d'Anthropologie préhistorique de Monaco, 31, 5-39.

Bellucci, L., Sardella, R., Rook, L. 2015. Large mammal biochronology framework in Europe at Jaramillo: The Epivillafranchian as a formal biochron. Quaternary International, 389, 84-89.

Caloi, L., Palombo, M.R. 1979. La fauna quaternaria di Venosa: Bovidi. Bollettino Servizio Geologico d'Italia, 100, 101-140.

Caloi, L., Palombo, M.R. 1979. La fauna quaternaria di Venosa: Canis sp. Quaternaria. Storia Naturale e Culturale del Quaternario Roma, 21, 115-128.

Cassoli, P.F., Di Stefano, G., Tagliacozzo, A. 1999. I vertebrati dei livelli superiori (A ed ALFA) della serie stratigrafica di Notarchirico. In: Piperno, M. (Ed.), Notarchirico. Un sito del Pleistocene medio iniziale nel bacino di Venosa. Osanna, Venosa, pp. 361-438.

Gliozzi, E., Abbazzi, L., Argenti, P., Azzaroli, A., Caloi L., Capasso Barbato, L., Di Stefano, G., Esu, D., Ficcarelli, G., Girotti, O., Kotsakis, T., Masini, F., Mazza, P., Mezzabotta, C., Palombo, M. R., Petronio, C., Rook, L., Sala, B., Sardella, R., Zanalda, E., Torre D. 1997. Biochronology of selected Mammals, Molluscs and Ostracods from the Middle Pliocene to the Late Pleistocene in Italy. The state of art. Rivista Italiana di Paleontologia e Stratigrafia 103, 369–388.

Sala, B. 1999. Nuovi dati sulla microteriofauna di Notarchirico. In: Piperno, M. (Ed.), Notarchirico un sito del Pleistocene medio iniziale nel bacino di Venosa. Ediz. Osanna per Soprint. speciale al Museo Nazionale Preistorico Etnografico "L. Pigorini", Roma, pp. 439–441.

Sardella, R., Palombo, M.R., Petronio, C., Bedetti, C., Pavia, M. 2006. The early Middle Pleistocene large mammal faunas of Italy: an overview. Quaternary International, 149 (2006), 104-109.

Sardella, R. 2007. Mid-Pleistocene vertebrate records from Europe. In AA.VV. Encyclopedia of Quartenary Sciences, 3224-3232.

Small mammals

Claudio Berto

University of Warsaw, Institute of Archaeology, Poland

Only 14 small mammal remains from layers H2, I1, I1c, I2a and I2b were recovered during the 2016 field campaign. Eight of these remains were identified as *Arvicola mosbachensis, Microtus (Terricola)* cf. *M. (T.) arvalidens*, and *Microtus* cf. *M. nivaloides* in layers I1, I1c and I2b (**Fig. S9**). The sample presents a high level of breakage and signs of corrosion, probably caused by the sediment 1. Insectivores and bats are absent from the observed record.
Arvicola mosbachensis is the most represented species. In all the analyzed specimens, the mimomyan enamel is recognizable while no rooted or incipient rooted teeth are present.
Microtus cf. *M. nivaloides* is only represented by a broken m1 with closed T4-T5 and confluent T6-T7 related to a well-developed anterior cap. The tooth is unrooted.
A m1 with a broken anterior cap is present in layer I2b. Even if the anterior cap is absent, the T4-T5 confluence is visible, allowing us to assign this tooth to *Microtus (Terricola)* cf. *M. (T.) arvalidens*.
Considering the species present in layers I1, I1c, and I2b, the small micromammal assemblage of Notarchirico is closely related to the material previously published by Sala2, allowing us to attribute this locality to the beginning of the Early Toringian (*Arvicola-Microtus* zone, Arvicola *mosbachensis* subzone) 3.

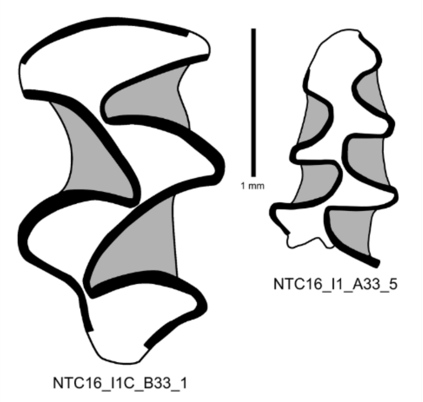

Figure S9. Two determined small mammals from Notarchirico: NTC16_I1C_B33_1: *Arvicola mosbachensis*, left M2; NTC16_I1_A33_5: *Microtus* cf. *M. nivaloides*, left m1.
(drawings C. Berto)

References

Fernandez-Jalvo, Y., Andrews, P. 2016. Atlas of taphonomic identifications : 1001+ images of fossil and recent mammal bone modification. Springer Science.

Sala, B. 1999. Nuovi dati sulla microteriofauna di Notarchirico. In: Notarchirico. Un sito del Pleistocene medio iniziale nel bacino di Venosa. Piperno, ed., pp. 439–441.

Sala, B., Masini, F. 2007. Late Pliocene and Pleistocene small mammal chronology in the Italian peninsula. Quaternary International 160, 4–16.

Taphonomic analysis

Camille Daujeard, Antonio Curci

Département Homme et Environnement, UMR 7194, HNHP, Muséum National d'Histoire Naturelle, Paris, France.
Dipartimento di Storia Culture Civiltà, Università di Bologna, Italy

Material

The studied material includes all the faunal elements from the 2016 to 2018 excavations. These remains concern layers F, G, H, I1, I2 and J, with a majority from layers F and I1 (Table S6). A total of 4,081 remains (NRT) have been studied, including 289 anatomically identified elements (NISPa), which only represent 7.1% of the NRT. Few elements are complete or almost complete (almost 14% of the NISPa). Among them, we observe a complete elephant humerus from layer H.

Methods

Taphonomic analysis was conducted on all the fossil remains. We report the total number of skeletal remains (NRT), the number of identified specimens (NISP), the minimum number of elements (MNE) and the minimum number of individuals present (MNI) (Binford, 1984; Lyman, 1994). We recorded the dimensions (length, breadth and thickness) and anatomical, taxonomic, and modification data for all the spatially recorded and identified specimens. Non-spatially recorded indeterminate fragments were only used for fragmentation studies (tissue types and size classes). The type of breakage (ancient green or dry bone fractures or recent fractures) was identified based on fracture color, shape, features and angle and associated marks (Villa and Mahieu, 1991; Blumenshine & Selvaggio, 1988, 1991). Shaft fragments were differentiated by size and circumference classes (Bunn, 1983; Villa and Mahieu, 1991).
For indeterminate fragments, we established four main size categories adapted to the ungulates present in our sample: 1) small sized-ungulates, including species with a weight between 10 and 100 kg (fallow deer); 2) middle-sized ungulates with a weight between 100 and 300 kg (red deer); 3) large-sized ungulates with a weight between 300 and 1,000 kg (large bovids and megaceros) and very large-sized ungulates with a weight superior to 1,000 kg (elephants).

As the sediments were partly accumulated by hydraulic factors, we tested Voorhies groups to evaluate the role of water transportation on the faunal assemblage. Voorhies groups classify the anatomical elements of mammals (> 10 kg) depending on their susceptibility to hydraulic transport. This method relies on the size and density of the elements. The relative representation of each group indicates whether an assemblage underwent fluvial transport, as well as the flow of the watercourse. According to experiments conducted by Behrensmeyer (1975), in addition to those of Voorhies, high-energy fluvial deposition environments, such as channel fills and lag deposits, tend to have high ratios of teeth-to-vertebrae, whereas low-energy environments, such as deltaic and lacustrine settings tend to have low ratios. Abrasion and the fluvial transportability of bones are the main taphonomic alterations in fluvial depositional environments.

Voorhies groups	Skeletal elements	Interpretation
**I**	Rib, vertebra, sacrum, sternum	Immediate transportation, even if water flow is low. The elements are removed by saltation or flotation
**I/II**	**Atlas**, scapula, ulna, phalange	Intermediate between groups I and II
**II**	Femur, tibia, humerus, radius, pelvis, metapodials, **astragalus, calcaneum**	Gradual transportation, remain in contact with bottom
**II/III**	Mandible	Residual material, lag deposit
**III**	Cranium, maxilla	
**NT**	Indeterminate, maxillar, fibula, others	Not applicable

Table S6. Voorhies groups based on Voorhies (1969), for complete bones of sheep and coyotes and on Behrensmeyer (1975), for sheep and horse (**in bold**).

Ontogenic age-at-death of prey specimens was based on dental eruption/replacement patterns and wear. We established four age groups: juveniles (with deciduous teeth), sub or young adults (with erupted P4 and M3), prime adults (with moderately worn P4 and M3) and old (with heavily worn teeth) (Grant, 1982; Klein and Cruz-Uribe, 1984).

We observed bone surfaces with the naked eye and we examined and photographed some elements to distinguish the various surface alterations using two Dino-Lite Digital Microscopes (AD7013MZT and AM7915MZT, magnification 20-220x). We recorded types and locations of relevant modifications on the outer surface, including those made by rodents, carnivores or hominins, as well as climatic and edaphic modifications. The latter include cracking, desquamation, polish, concretion, root marking, chemical corrosion and oxide coloration. The identification of the main taphonomic modifications was based on the criteria defined by Behrensmeyer (1978), Binford (1981), Lyman (1994) and Fisher (1995) (among many others). The illegible remains were not included in the percentages.
We specifically distinguished trampling marks from butchering marks with reference to works by Binford (1981), Shipman & Rose (1983, 1984), Behrensmeyer et al. (1986), Olsen and Shipman (1988), Blasco et al. (2008) and Dominguez-Rodrigo et al. (2009). Based on Binford (1981), Haynes (1983) and Campmas and Beauval (2008), we classified carnivore marks as follows: pits, punctures, scores, notches or corrosion by gastric acids. We took measurements of pits, punctures, scores and notches (maximal length – L – and breadth – W –) into account, as well as tissue location (cancellous bone or articular portions; cortical or median diaphyses; thin cortical bone or diaphysis extremities) (see Dominguez and Pickeras, 2003; Pickering et al., 2004).

Faunal assemblages

Among the identified fauna, cervids largely dominate the spectrum in layers H and I1, while elephants prevail in layers F, G and I2 (**Table S7**). Large bovids (cf. *Bos*/*Bison*) are quite important in levels F and I2. Two new species were identified as a result of recent excavations; the hippopotamus in layers G and I1, and the macaque in layer G. No carnivores have yet been identified.
Red deer and megaceros are mostly represented by remains of juveniles and subadults in layers H and I1 (**Table S8**). In the latter, cervids are represented by all parts of the skeleton: cranial, axial, girdle and appendicular elements (**Table S9**). Isolated teeth, ribs and small articular bones (carpals, calcanei and phalanges) dominate. Long bones are scarce.
Large-sized bovids have been identified mainly on the presence of teeth and metapodials. Prime-aged and old adults predominate in layers F, G and I1. One subadult is present in layer I2.

Elephants consist mostly of fragments of tusks, teeth and indeterminate bone fragments.
Finally, the hippopotamus is represented by four tooth fragments, one of which belongs to a young individual in layer I1, and the macaque by the proximal end of an ulna.
Concerning the skeletal distribution for all species and layers combined, Voorhies groups indicate the predominance of many elements from the two first groups (mainly trunk and phalanges) (**Tables 10, 11**), which are usually the first elements to be sorted by fluvial transport. Thus, skeletal distribution does not *a priori* support a lag deposit, but rather a shore deposit or a low-energy deltaic or lacustrine environment, with some possible more violent hydraulic transport episodes.

Bone fragmentation

The fauna is heavily fragmented, mostly represented by isolated teeth and bone fragments. For levels F and I1, which have the most numerous faunal remains, the distribution of fragments by size class underlines a majority of small elements less than 50 mm. Only 7 and 9 remains respectively are long bone elements with a shaft length greater than or equal to the L3 class size and only 2 and 22 bone elements are complete or almost complete. The identification indexes confirm the high degree of bone fragmentation, especially in level F (**Tables S6, S12, S13 and S14**).
Post-depositional dry bone fractures are the most recurrent type of fragmentation in both levels (Table 9). Green bone fractures (curved, oblique and smooth edges) are also largely present, especially among long bone elements. Recent breakages are also very common and indeterminate fractures are present on almost a third of the bone remains. Some natural notches (internal or cortical) are sometimes associated with green bone fractures, pointing to violent post-depositional events, such as hydraulic transport or trampling.
Thus, all these criteria point to penecontemporaneous fragmentation (trampling, hydraulic transport and possibly anthropogenic bone breakage), as well as post-depositional fragmentation (sediment pressure and/or hydraulic transport).

Bone preservation and surface modifications

Bone destruction indexes seem to be similar in the three main assemblages F, G and I1, indicating a relatively good state of bone preservation (**Table S6; Fig. S1, S2, S3**). Nevertheless, post-depositional modifications, such as cracking, desquamation, concretions, chemical corrosion and abrasion (polish, gloss and random striations) appear to have been quite destructive for bone surfaces, and here again, particularly in level F (**Table S4**). Between 40 and 20% of the bone elements respectively in levels F and I1 present illegible surfaces. Abrasion striations, concretions and oxide colorations are all more abundant in level F. Most of the modifications may be related to the effects of hydraulic transport and trampling (abrasion) and exposure to water (corrosion, concretions). White and thin crusts, possibly calcite, covering part of the bone surfaces, were observed on more than half of the remains in both levels. Others, such as cracking, desquamation and root marking, also widely present, result from climatic and edaphic alterations. Very scarce carnivore marks were identified (n=3) in level I1 and none elsewhere. No cut marks or clear anthropic percussion marks have been identified.

Discussion / Conclusion

The fauna of Notarchirico (outside trench) is composed of various ungulates and primates, and with the exception of two new species, the hippopotamus and the macaque, the faunal list is quite comparable to that of level Alpha (Cassoli et al., in Piperno (dir.), 1999).

The assemblage is mainly composed of indeterminate bone fragments and no anatomical connections have been observed. Interestingly, anatomical representation is quite similar to that of level Alpha, i.e., an abundance of limb extremities for cervids and an abundance of isolated teeth and

long bone shaft fragments for bovids (Tagliacozzo et al., in Piperno (dir.), 1999). Again, like for level Alpha, elephants are mainly represented by head elements, including some tusk and tooth fragments.
Concerning the Voorhies groups, for all species and levels, the relative scarcity of long bones and cranial remains and the abundance of short elements rule out a simple lag deposit context. These series would rather represent successive episodes of deposition, including some violent water flows producing hydraulic sorting of the bone elements.

The fauna is heavily fragmented. Some green bone fractures associated with notches have been recorded. It is difficult to distinguish the anthropic or post-depositional origin of the latter, and they are possibly caused by natural violent impacts (trampling or strong water flow). Thus, contrary to level Alpha where a few notches were linked to anthropic percussion, here we could not confidently identify percussion marks. No or very scarce elements bear carnivore marks (level I1) and no cut marks could be identified. Moreover, many random striations and smoothed edges were observed on the bone surfaces, underlining the strong effects of abrasion on the bone assemblage. For level Alpha, more than half of the bone remains were abraded to various degrees. Thus, natural abrasion is the most common post-depositional modification for the whole sequence of Notarchirico.

The bone assemblage from the outside trench represents a mixture of multiple deposits of animal carcasses, the majority of which may have naturally died in the surroundings of this water channel / lacustrine context, and may then have been secondarily transported, sorted and modified. Hydraulic transport and trampling are both the main causes of post-depositional alterations.

Finally, while there is not yet any evidence to support human or carnivore contributions to the accumulation of the bone assemblage, we cannot exclude the possibility that some animals may have been accumulated and/or consumed *in situ* by both types of predators and scavengers.

References

Behrensmeyer, A.K. 1975. The taphonomy and paleoecology of Plio-Pleistocene vertebrate assemblages east of Lake Rudolf, Kenya. Bulletin of the Museum of Comparative Zoology 146, 473-578.

Behrensmeyer, A.K. 1978. Taphonomic and ecologic information from bone weathering. Palaeobiology 4, 150–162.

Behrensmeyer, A.K., Gordon, K.D., Yanagi, G.T. 1986. Trampling as a cause of bone surface damage and pseudo-cutmarks. Nature 319, 768-771.

Binford, L.R. 1981. Bones: Ancient Men and Modern Myths. Academic Press, New York.

Binford, L.R. 1984. Faunal Remains from Klasies River Mouth. Academic Press, New York.

Blasco, R., Rosell, J., Peris, J.F., Caceres, I., Vergès, J.M. 2008. A new element of trampling: an experimental application on the Level XII faunal record of Bolomor Cave (Valencia, Spain). Journal of Archaeological Science, 35, 1605-1618.

Blumenschine, R.J., Selvaggio, M.M. 1988. Percussion marks on bone surfaces as a new diagnostic of hominid behavior. Nature, 333, 763-765.

Blumenshine, R.J., Selvaggio, M.M. 1991. On the marks of marrow bone processing by hammerstones and hyenas: their anatomical patterning and archaeological implications. In: Clark, J.D. (Ed.), Cultural

Beginnings – Approaches to Understanding Early Hominid Life-ways in the African Savanna, UISPP, 1987, Mainz, pp. 17–32.

Bunn, H.T. 1983. Comparative analysis of modern bone assemblage from a san hunter-gatherer camp in the Kalahari desert, Botswana, and from a spotted hyena den near Nairobi, Kenya. In Animals and Archaeology: 1. Hunters and their Prey, J. Clutton-Brock & C. Grigson édit., BAR International Series, 163, pp. 143-148.

Campmas, E., Beauval, C. 2008. Consommation osseuse des carnivores: résultats de l'étude de l'exploitation de carcasses de boeufs (*Bos taurus*) par des loups captifs. L'Anthropologie, 94, 167-186.

Dominguez-Rodrigo, M., Piqueras, A. 2003. The use of tooth pits to identify carnivore taxa in tooth-marked archaeofaunas and their relevance to reconstruct hominid carcass processing behaviours. Journal of Archaeological Science 30, 1385-1391.

Domínguez-Rodrigo, M., de Juana, S., Galan, A.B., Rodriguez, M. 2009. A new protocol to differentiate trampling marks from butchery cut marks. Journal of Archaeological Science 36, 2643-2654.

Grant A. 1982. The use of tooth wear as a guide to the age of domestic ungulates. In Wilson, B., Grigson, C. & Payne, S., eds.), Ageing and sexing animal bones from archaeological sites, British Archaeological Reports International Series, S109, pp. 91-108.

Haynes, G. 1983. A guide for differentiating mammalian carnivore taxa responsible for gnaw damage to herbivore limb bones. Paleobiology, 9 (2), 164-172.

Klein, R.G., Cruz-Uribe, C. 1984. The Analysis of Animal Bones from Archaeological Sites. University of Chicago Press.

Fisher, J.W. 1995. Bone surface modifications in zooarchaeology. Journal of Archaeological Method and Theory 2(1), 7-68.

Lyman R.L. 1994. Vertebrate Taphonomy. Cambridge University Press, 524 p.

Olsen S. L., Shipman P. 1988) Surface modification on bone: trampling versus butchery. Journal of Archaeological Science, 15, 535-553.

Piperno, M. (Ed.) 1999. Notarchirico. Un sito del Pleistocene medio iniziale nel bacino di Venosa. Edizioni Osanna, Italia.

Shipman P., Rose J. J. 1983. Early hominid hunting, butchering, and carcass processing behaviors: approaches to the fossil record. Journal of Anthropological Archaeology, 2, 57-98.

Shipman P., Rose J. J. 1984. Cutmark Mimics on Modern and Fossil Bovid Bones. Current Anthropology, 25, 1, 116-117.

Pickering, T.R., Dominguez-Rodrigo, M., Egeland, C.P., Brain, C.K. 2004. Beyond leopards: tooth marks and the contribution of multiple carnivore taxa to the accumulation of the Swartkrans Member 3 fossil assemblage. Journal of Human Evolution, 46, 595-604.

Villa P., Mahieu E. 1991. Breakage patterns of human long bones. Journal of Human Evolution 21, 27-48.

Voorhies, M. 1969. Taphonomy and population dynamics of an early Pliocene vertebrate fauna, Knox County, Nebraska. University of Wyoming Contributions to Geology Special Paper No. l. Laramie.

Table S6. NR, NISP and various preservation indexes for the studied series (complete elements concern bones and teeth).

	E/F	F	G	H	I1	I2	J	Total
NRT	204	1218	569	236	1681	167	6	**4081**
NR isolated teeth		11	6	6	37	4		**64**
NISP (rank of family)		35	21	12	99	29		**196**
NISPa		49	25	13	170	32		**289**
Identification index	0,0%	4.0%	4.4%	5.5%	10.1%	19.2%	0.0%	**7.1%**
Bone destruction index		22.4%	24.0%	46.2%	21.8%	12.5%		**22.1%**
NR recorded		147	61	25	294	61	1	**589**
Complete elements		9	2	3	22	4		**40**
Completeness		18.4%	8.0%	23.1%	12.9%	12.5%		**13.8%**

Table S7. Faunal spectrum for the faunal assemblages and indeterminate remains (NISP and %NISP).

	E/F	F	F	G	G	H	H	I1	I1	I2	I2	J	Total NISP	%NISP
Palaeoloxodon antiquus		16	45.7%	17	81.0%	1	8.3%	8	8.1%	14	48.3%		**56**	**28.6%**
Hippopotamus antiquus			0.0%	2	9.5%		0.0%	2	2.0%		0.0%		**4**	**2.0%**
Cervus elaphus		1	2.9%		0.0%	4	33.3%	21	21.2%	2	6.9%		**28**	**14.3%**
Praemegaceros solihacus			0.0%		0.0%	3	25.0%	16	16.2%	1	3.4%		**20**	**10.2%**
Medium-sized cervids		2	5.7%		0.0%	2	16.7%	20	20.2%	1	3.4%		**25**	**12.8%**
Large-sized cervids		1	2.9%		0.0%	2	16.7%	23	23.2%		0.0%		**26**	**13.3%**
Total cervids		4	11.4%	0	0.0%	11	91.7%	80	80.8%	4	13.8%	0	**99**	**50.5%**
Bison schoetensacki		3	8.6%	1	4.8%		0.0%	1	1.0%	3	10.3%		**8**	**4.1%**
Bos/Bison		12	34.3%		0.0%		0.0%	8	8.1%	8	27.6%		**28**	**14.3%**
Total bovines		15	42.9%	1	4.8%	0	0.0%	9	9.1%	11	37.9%		**36**	**18.4%**
Macaca sylvanus			0.0%	1	4.8%		0.0%		0.0%		0.0%		**1**	**0.5%**
**Total NISP**	**0**	**35**	**100.0%**	**21**	**100.0%**	**12**	**100.0%**	**99**	**100.0%**	**29**	**100.0%**	**0**	**196**	**100,0%**
Small-sized ungulates			0.0%		0.0%	2	0.8%	12	0.7%		0..0%		**14**	**0.3%**
Middle-sized ungulates		43	3.5%	15	2.6%	11	4.7%	135	8.0%	8	4.8%	1	**213**	**5.2%**
(Very) large-sized ungulates		66	5.4%	19	3.3%	2	0.8%	55	3.3%	19	11.4%		**161**	**3.9%**
Indeterminate	204	1074	88.2%	514	90.3%	209	88.6%	1380	82.1%	111	66.5%	5	**3497**	**85.7%**

**Total Indeterminate**	**204**	**1183**	**97.1%**	**548**	**96.3%**	**224**	**94.9%**	**1582**	**94.1%**	**138**	**82.6%**	**6**	**3885**	**95.2%**
**NRT**	**204**	**1218**	**100.0%**	**569**	**100.0%**	**236**	**100.0%**	**1681**	**100.0%**	**167**	**100.0%**	**6**	**4081**	**100.0%**

Table S8. Age categories and Minimum Number of Individuals (MNI) (Ad IND: individuals of undetermined age; YA: Young or Sub-Adults; JUV: Juveniles; MA: Prime-aged adults; OA: Old Adults).

	**F**	**G**	**H**	**I1**	**I2**	**Total MNI**
Palaeoloxodon antiquus	1 IND	1 IND	1 IND	1 IND	1 IND	**5**
Hippopotamus antiquus		1 IND		1 YA		**2**
Cervus elaphus	1 MA		1 IND	1 JUV, 1 YA	1 IND	**5**
Praemegaceros solihacus			1 YA	1 JUV	1 IND	**3**
Large-sized cervids	1 IND		1 JUV	1 OA		**3**
Bos/Bison	1 PA, 1 OA	1 PA		1 OA	1 YA	**5**
Macaca sylvanus		1 IND				**1**
**Total MNI**	**5**	**4**	**4**	**7**	**4**	**24**

Table S9. Anatomical element identification for each species (NISP).

Anatomical elements	*Hippopotamus*			*Palaeoloxodon*					cervids (small/middle-sized)				cervids (large/very large-sized)				*Bos/Bison*			
Stratigraphical units	F	G	I1	F	G	H	I1	I2	F	H	I1	I2	F	H	I1	I2	F	G	I1	I2
Horn/Antler													1				1			
Tusk				9	4			4												
Skull								1			1									
Mandible											2				1		1			
Isolated teeth (lower)									1		6				2		2	1	1	1
Isolated teeth (upper)											2				1		3			
Isolated teeth (indet.)		2	2	6	1		4	1	1	3	5			1	1		3		2	2
Cervical vertebrae											2				2				1	1
Thoracic vertebrae					1						1				2					1
Lumbar vertebrae											1				1					
Indeterminate vertebrae					1						1				1					

Sacrum																				
Pelvis											2				1					
Rib				1	1						9				1					
Scapula								2							1					1
Humerus						1					1				1					
Radio-ulna										1	1				4		1		2	1
Carpal										1	1				2					
Metacarpal									1		1				1					2
Femur															2					
Tibia															2	1	2		1	
Talus												2		1	1					
Calcaneum											1				1				2	
Cubonavicular																	1			
Metatarsal											2				1					1
Phalanx 1										1				1	3					
Phalanx 2														2	5		1			
Phalanx 3																				
Metapodials											2	1			2					1
Sesamoid																				
Indeterminate					9		4	6												
Total NISP	0	2	2	16	17	1	8	14	3	6	41	3	1	5	39	1	15	1	9	11

Table S10. Distribution of all skeletal elements according to the Voorhies groups (adapted from Voorhies, 1969 and Behrensmeyer, 1975).

**Anatomical elements**	**Voorhies groups**				
	**I**	**I/II**	**II**	**II/III**	**III**
Horn/Antler					2
Tusk					17
Skull					9
Mandible				7	
Isolated teeth (lower)				15	
Isolated teeth (upper)					6
Isolated teeth (indet.)				39	
Cervical vertebrae	7	1			
Thoracic vertebrae	9				
Lumbar vertebrae	6				
Indeterminate vertebrae	16				
Sacrum	0				
Pelvis			4		
Rib	40				
Scapula		10			
Humerus			4		
Radio-ulna		13			
Carpal		6			
Metacarpal			7		
Femur			3		
Tibia			13		
Talus			5		
Calcaneum			4		
Cubonavicular					
Metatarsal			4		

Phalanx 1		5			
Phalanx 2		9			
Phalanx 3					
Metapodials			8		
Sesamoid					
Indeterminate					
**Total NISP**	**78**	**44**	**52**	**61**	**34**

Table S11. Voorhies groups based on Voorhies (1969) for complete bones of sheep and coyotes and on Behrensmeyer (1975) for sheep and horse (**in bold**).

**Voorhies groups**	**Skeletal elements**	**Interpretation**
**I**	Rib, vertebra, sacrum, sternum	Immediate transportation, even if the water flow is low. The elements are removed by saltation or flotation
**I/II**	**Atlas**, scapula, ulna, phalange	Intermediate between the groups I and II
**II**	Femur, tibia, humerus, radius, pelvis, metapodials, **astragalus, calcaneum**	Gradual transportation, stay in contact with bottom
**II/III**	Mandible	Residual material, lag deposit
**III**	Cranium, maxilla	
**NT**	Indeterminate, maxillary, fibula, others	Not applicable

Table S12. Size classes (mm) for bone elements in layers F and I1 (NR: Number of Remains).

**Size classes**	**0-25**	**26-50**	**51-75**	**76-100**	**101-125**	**126-150**	**151-175**	**176-200**	**201-225**	**226-250**	**251-275**	**276-300**	**Total**
NR (unit F)	424	678	38	30	5	6	5	3	3	1	1	2	1196
% (unit F)	35.5%	56.7%	3.2%	2.5%	0.4%	0.5%	0.4%	0.3%	0.3%	0.1%	0.1%	0.2%	100.0%
NR (unit I1)	868	652	61	30	11	6	4	3	1	1	2	0	1639
% (unit I1)	53.0%	39.8%	3.7%	1.8%	0.7%	0.4%	0.2%	0.2%	0.1%	0.1%	0.1%	0%	100.0%

Table S13. Length and circumference classes of long bone elements in layers F and I1 (cf. Villa and Mahieu, 1991).

**Layer F**	L1	L2	L3	L4	**TOTAL C**	**Layer I1**	L1	L2	L3	L4	**TOTAL C**
C1	7	35	4	2	48	C1	16	32	5	1	54
C2		1	1		2	C2		2	1		3
C3					0	C3	2	1	2		5
**TOTAL L**	7	36	5	2	50	**TOTAL L**	18	35	8	1	62

Table S14. Climatic, edaphic and biotic surface alteration (stages 1 to 3) and bone fragmentation indexes (Number of total recorded elements: NR=154 for level F and NR=311 for layer I1; Number of total bone elements: NR=1196 and NR=1643; Number of long bone elements: NR=67 and NR=94; Number of recorded bone elements: NR=131 and NR=28; and Number of legible recorded bone elements: NR=84 and NR=216).

	**unit F**						**unit I1**					
stages	**1**	**2**	**3**	**n Total**	**NR total**	**%**	**1**	**2**	**3**	**n Total**	**NR total**	**%**
cracking	46	25	18	89	154	57.8%	106	47	32	185	311	59.5%
desquamation	15	15	11	41	154	26.6%	70	64	14	148	311	47.6%
smooth edges	18	18	3	39	154	25.3%	40	40	11	91	311	29.3%
glossy				2	154	1.3%				20	311	6.4%
abrasion (striations)				70	154	45.5%				85	311	27.3%
concretion/calcite	68	41	16	125	154	81.2%	106	68	23	197	311	63.3%
white crusts				125	154	81.2%				179	311	57.6%
chemical corrosion	61	28	2	91	154	59.1%	121	75	8	204	311	65.6%
root marking	45	34	9	88	154	57.1%	71	67	21	159	311	51.1%

oxides (black coloration)	77	12	1	90	154	58.4%	96	11		107	311	34.4%
illegible remains				59	154	38.3%				71	311	22.8%
bone completeness				2	1196	0.2%				22	1643	1.3%
green bone fracture (long bones)				48	67	71.6%				62	94	66.0%
green bone fracture (all bones)				59	131	45.0%				90	281	32.0%
notches (cortical and internal)				16	131	12.2%				17	281	6.0%
dry bone fracture				76	131	58.0%				123	281	43.8%
recent fracture				118	131	90.1%				185	281	65.8%
Indeterminate fracture				41	131	31.3%				85	281	30.2%
carnivore marks				0	84	0.0%				3	216	1.4%

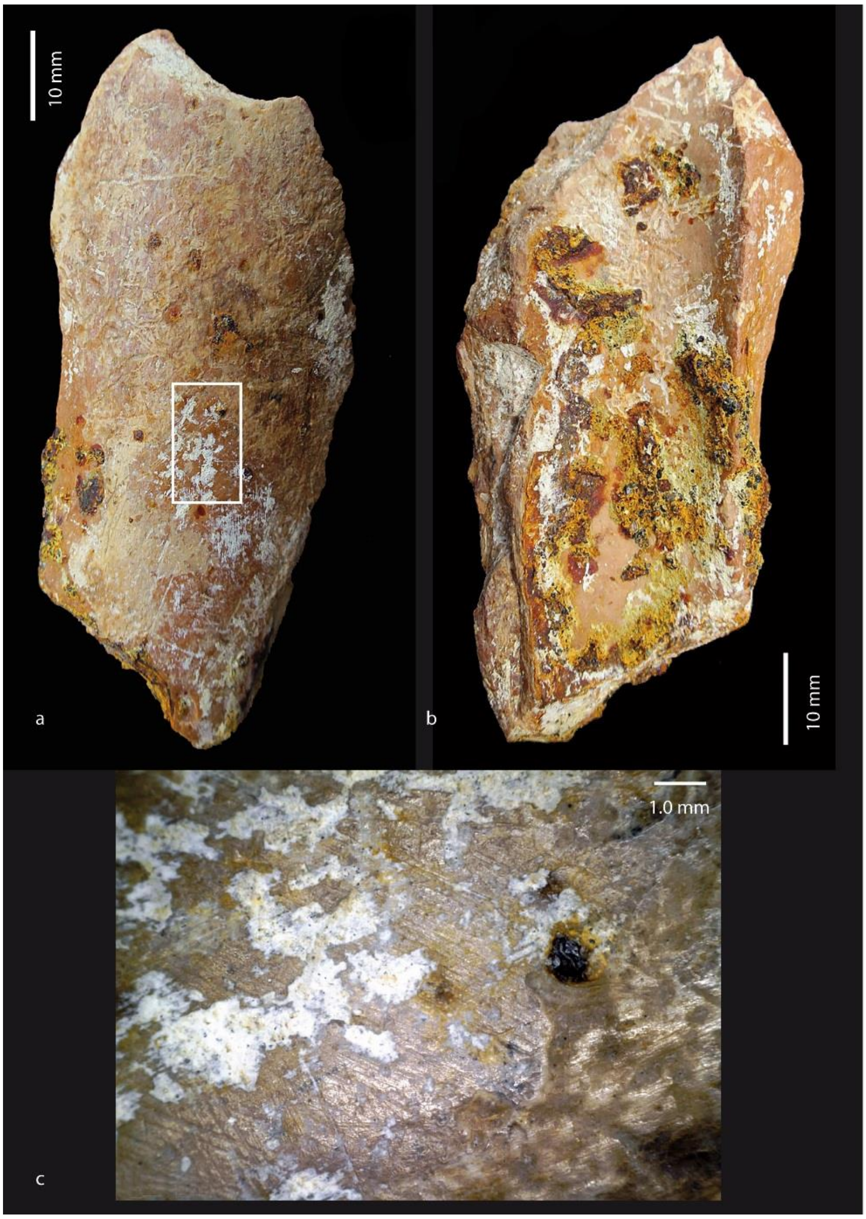

Figure S10.. a-b, Views of the cortical and internal sides of an unspecified bone shaft fragment (NOT16-A33-30-I1a). Note the white crusts, concretions, random striations, root etchings dissolution, green bone fractures and associated internal notch. c, Magnification of the encrusting and micro-abrasion striations (Digital microscope image by C. Daujeard).

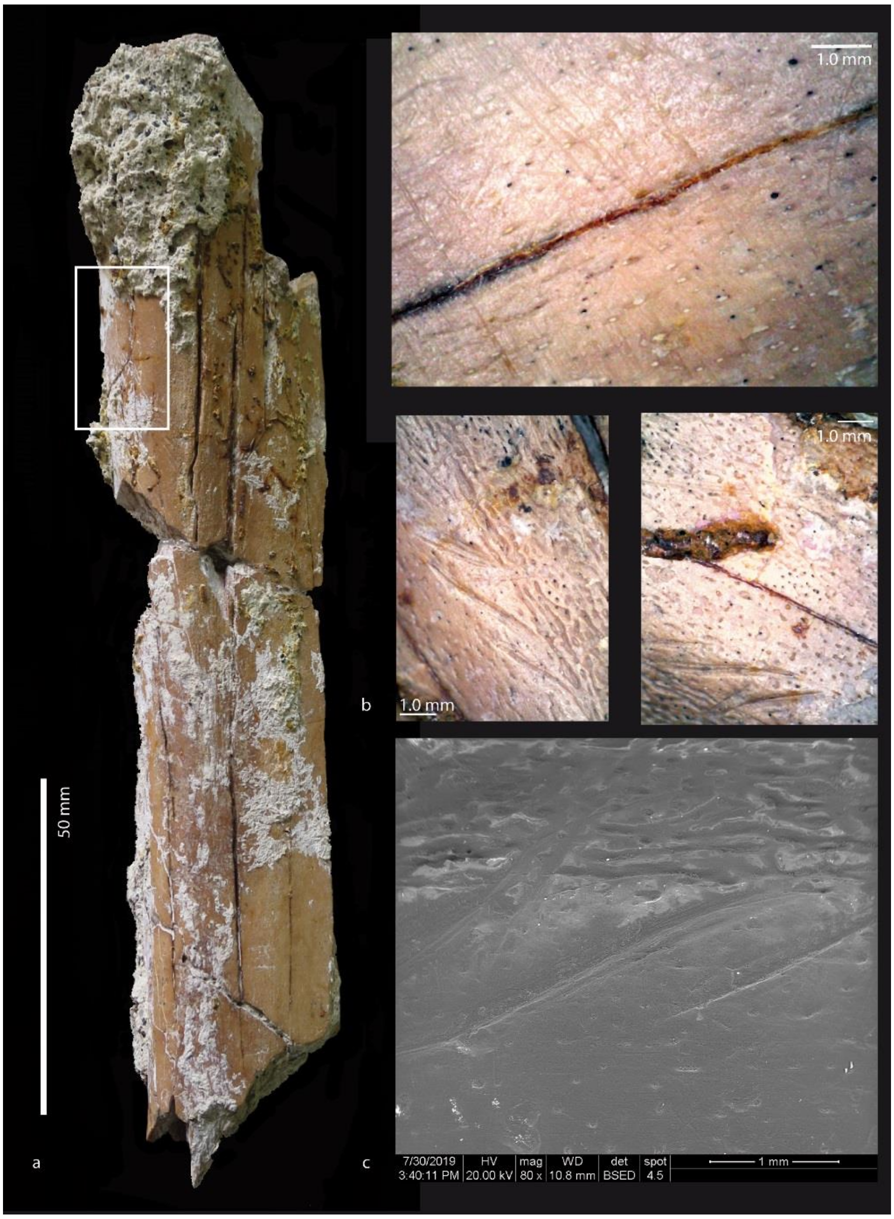

Figure S11. a, View of the cortical side of a tibia shaft fragment of an unspecified large ungulate (NOT18-Z17-8-F). Note the encrusting, cracking and random striations. b-c, Magnification of the pseudo cut marks and micro-abrasion striations (Digital microscope image by C. Daujeard and SEM by A. Curci).

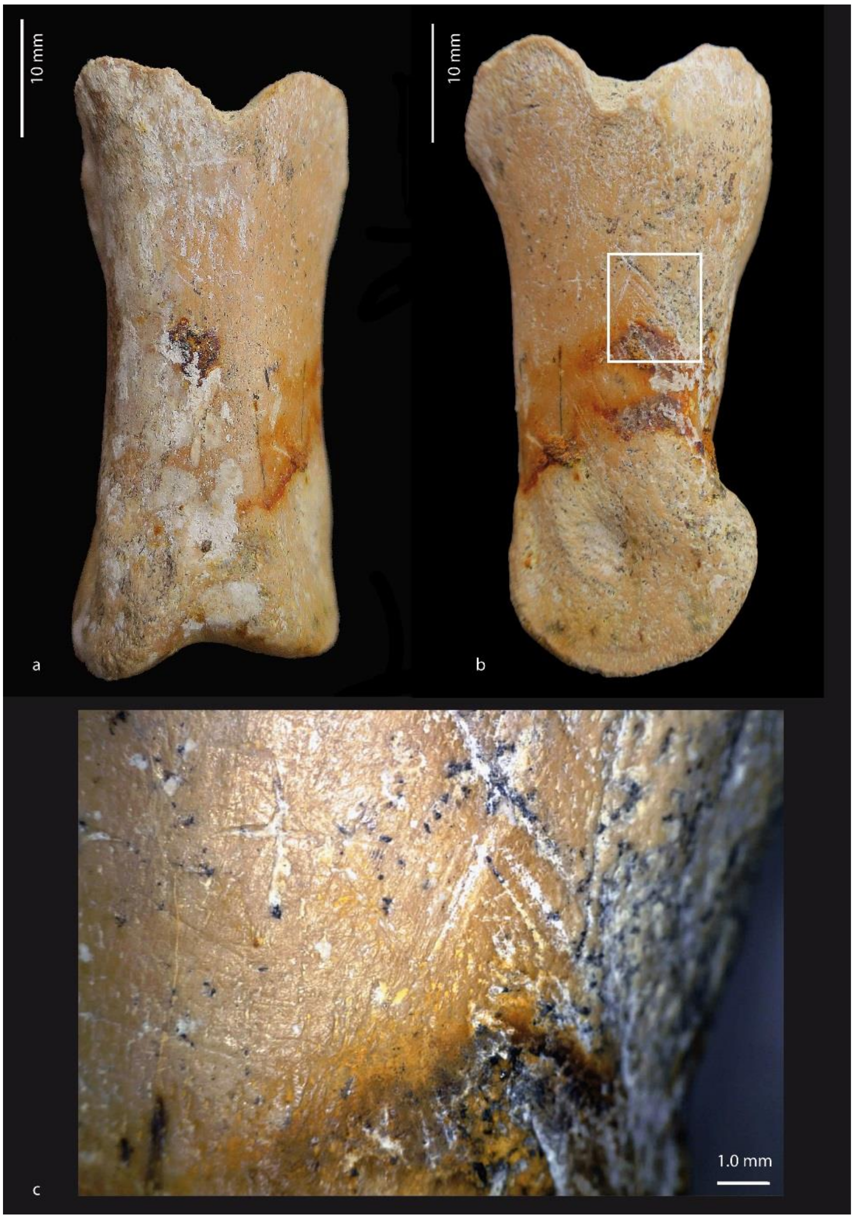

Figure S12. a-b, Phalanx 1 of a large-sized cervid (*Cervus* or *Praemegaceros*) belonging to a juvenile (unfused proximal epiphysis) (NOT16-B29-1-H). We observe oxide colorations and random striations caused by natural abrasion. c, Magnification of the lateral side of the phalanx (Digital microscope image by C. Daujeard).

General view of the summet of the layer I2 under excavation. Bed of pebbles-cobbles

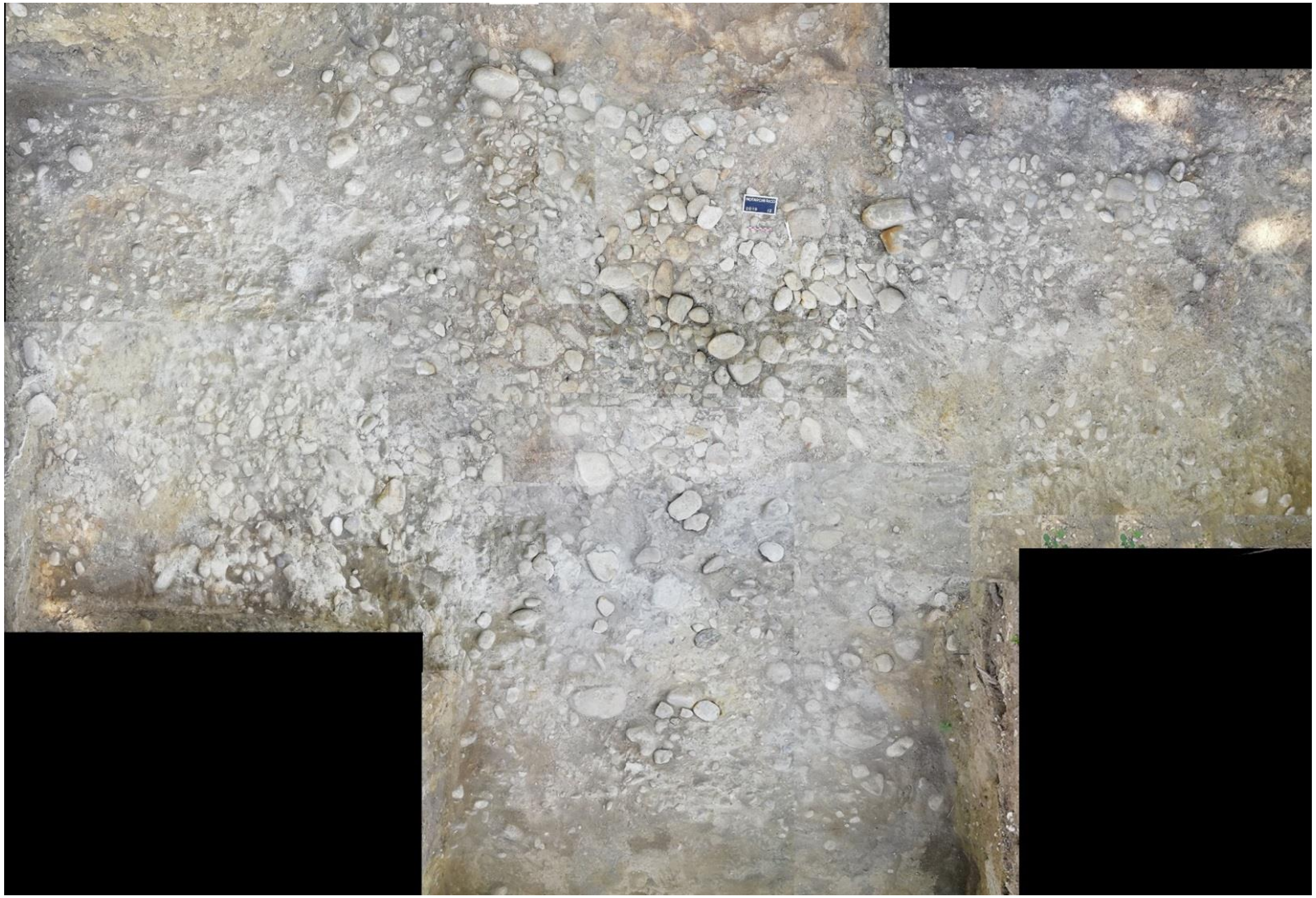

Figure S13. General view of the summet of the layer I2 under excavation. Bed of pebbles-cobbles (photo M-H. Monce, C. Santagata).

First Petrographic and Chemical Analyses of Chert Artefacts

Giacomo Eramo (1), Ignazio Allegretta (2), Emanuela Delluniversità (1), Roberto Terzano (2)

1) Dipartimento di Scienze della Terra e Geoambientali, Università degli Studi di Bari "Aldo Moro", Bari, 70125, Italy
2) Dipartimento di Scienze del Suolo, della Pianta e degli Alimenti, Università degli Studi di Bari "Aldo Moro", Bari, 70126, Italy

Aims

A selection of archaeological and geological chert samples were studied to assess the available lithological variability and to verify the archaeological hypothesis of the exploitation of local secondary chert to produce artefacts, as well as the hypothesis of lithological selection.

Sampling Strategy

The archaeological chert sample consists of 76 pieces, selected from the findings of the excavations carried out in 2016 (n = 33) and 2017 (n = 43). Sampling privileged the larger artefacts and attempted to include the full range of chert variability.

Analytical Methods

The petrographic description was carried out using a non-destructive multi-parametric protocol for chert investigations (NM-PCI), using a formalized mixed-data matrix (**Fig. S14**), according to Tarantini et al. (2016) and Delluniversità et al. (2019).

The NM-PCI uses a formalized description of macroscopic and mesoscopic characteristics (e.g., structure, texture, fracture), expressed in binary or ordinal variables, as well as colorimetric (i.e., CIEL*a*b*) and geochemical (i.e., K, Ca, Ti, Mn, Fe, Ni, As, Sr, Ba) continuous variables of the chert matrix, acquired with portable devices (Konica-Minolta CM-2600d spectrophotocolorimeter and a Thermo-NITON XL3t ED-XRF spectrometer) (Delluniversità et al., 2019). A selection of the transformed mixed data variables (Gower, 1971) was statistically processed and visualized (i.e., PAM, t-SNE) in R software environment (R core Team, 2018), with the following packages: *cluster* (Maechler et al., 2018) for data transformation, PAM algorithm and silhouette width; *ggplot2* (Wickham, 2016) and *Rtsne* (Krijthe, 2015) for output visualization; and *fpc* (Hennig, 2014), for internal and external clustering validation.

Petrography

The size of the chert artefacts ranges from 17 mm to 95 mm, with a median of 30 mm (**Fig. S15**). Four main lithotypes were identified among the samples: i) silicified litharenites (flysch chert); ii) nodular chert; iii) vitreous chert; iv) radiolarite.

Almost all of the samples show the partial presence of a neocortex, demonstrating the secondary origin of the raw materials.

The texture of silicified litharenites varies between wakestone and packstone (Dunham, 1962) and the sorting of grains is medium to high (**Fig. S16 a, b**). Part of these samples show some porosity, mainly in level F (post-depositional conditions?). Among the recrystallized allochems, some benthic foraminifera and sponge spicules were identified (1c). Despite their sandy texture, these lithotypes show a sub-conchoidal to conchoidal fracture. The nodular chert displays a spotted structure, with sponge spicules, and suggests shallower marine depositional environments. The radiolarite samples (1e, f) show colors ranging between orange and dark brown, with a spotted and sometimes laminated structure. Four samples (NOT28, NOT106, NOT126, NOT127) show a vitreous aspect and a homogeneous structure.

Such lithotypes may be associated with the Flysch Rosso Fm primary source (Gallicchio et al., 2008), which supplied secondary deposits to the area of Notarchirico (Synthem of Palazzo San Gervasio) (ISPRA, in press).
Silicified litharenites constitute 80% of the samples from layer F, whereas the rest are in nodular chert. A different distribution was observed in layer G, where 50% of the samples are silicified litharenites, 25% nodular chert and 25% radiolarite. In layers H_2*, silicified litharenites and nodular chert occur in similar proportions. The same trend was observed in layers I_1* and I_2*, with the presence of some radiolarite in I_2 and I_{2a} and vitreous chert in layers I_{1bc} and I_{2a}.

Surface Features
The color of the artefacts is sometimes affected by alteration due to different types and degrees of patination. In most samples, the presence of faint glossy patina (Howard, 2002) does not affect the color of the samples. White (desilication) patina is sporadic. Such features are compatible with the pH values of the excavated layers (i.e., F – I), ranging between 5 and 6, which are quite low for silica dissolution. A black patina was observed on six samples. In NOT104 and NOT122, the patina formed before the working of pebbles (plate 1h), whereas in NOT32, NOT100, NOT131 and NOT136, it formed in post-depositional conditions (plate 1g). Due to non-significant increases of Mn and Fe concentrations on the surface, it cannot be classified as black varnish (Pawlinowski & Wasilewski, 2002).
Although the color was measured on all the samples, it was not always possible to measure the real color(s) of chert due to the different degrees of patination among samples. Such heterogeneity in the accuracy of color excludes the CIE L*a*b* coordinates from the multivariate statistics.

Chemistry
The chemical composition of the samples shows relevant differences in concentration. Calcium, iron and potassium show far higher median values than those for the other elements measured here (Tab. 1). A strong positive correlation occurs between Fe and As (r = 0.87), Sr and Ba (r = 0.83) and Ti and K (r = 0.72).
Due to the high variability of the Ca content and the constant presence of carbonate concretions on the samples, Ca was excluded as a variable for multivariate statistics.

Discussion
The best classification was obtained using a selection of chemical (8), structural (7) and textural (2) variables (**Fig. S14**). The PAM algorithm run on ten clusters (max. silhouette width = 0.34) shows that vitreous chert (cluster 7) is quite distinct from the rest of the samples, whereas silicified litharenites are less differentiated, except for clusters 2 and 10 (flysch ps), from nodular chert and more similar to flysch ws (mainly clusters 5 and 8) and radiolarite (clusters 3 and 4).
Due to the general presence of the neocortex and the small size of artefacts, secondary chert was used as a raw material.
The relative abundance of flysch chert among artefacts, compared to the other chert varieties, is interpreted as a consequence of its probable abundance in the secondary deposits. A possible selection of chert lithotypes should be proven by a comparison of the artefacts with the polygenic conglomerates available in the area.

References

Delluniversità, E., Muntoni, I.M, Allegretta, I., Tarantini M., Monno A., Maiorano P., Girone A., Morsilli M., Terzano R., Eramo G. 2019. Development of a multi-parametric characterisation protocol for chert investigation and application on the Gargano Promontory mines. Archaeological and Anthropological Sciences. doi: 10.1007/s12520-019-00875-8.

ISPRA. (in press). Carta Geologica d'Italia, Foglio 452 "Rionero in Vulture". L.A.C. srl.

Gower, J.C. 1071. A general coefficient of similarity and some of its properties. Biometrics, 857-871.

Hennig, C. 2014. Flexible procedures for clustering [Computer software manual].

Howard, C. D. 2002. The gloss patination of flint artifacts. Plains anthropologist, *47*(182), 283-287.

Krijthe J.H. 2015. T-Distributed Stochastic Neighbor Embedding using a Barnes-Hut Implementation. URL: https://github.com/jkrijthe/Rtsne.

Maechler, M., Rousseeuw, P., Struyf, A., Hubert, M., Hornik, K. 2018. Cluster Analysis Basics and Extensions. R package version 2.0.7-1.

Pawlinowski, M., Wasilewski, M. 2002. Mineralogical investigation of desert patina on flint artifacts: A case study. Mediterranean Archaeology and Archaeometry, *2*(2), 23-34.

R Core Team 2018. A language and environment for statistical computing. R Foundation for Statistical Computing, Vienna, Austria. URL http://www.R-project.org/.

Tarantini, M., Eramo, G., Monno, A., Muntoni, I.M. 2016. Gargano Promontory Chert: mining practices and archaeometric characterisation, Séances de la Société préhistorique française, 5, 249-267.

Wickham, H. 2016. Elegant Graphics for Data Analysis, Springer-Verlag New York.

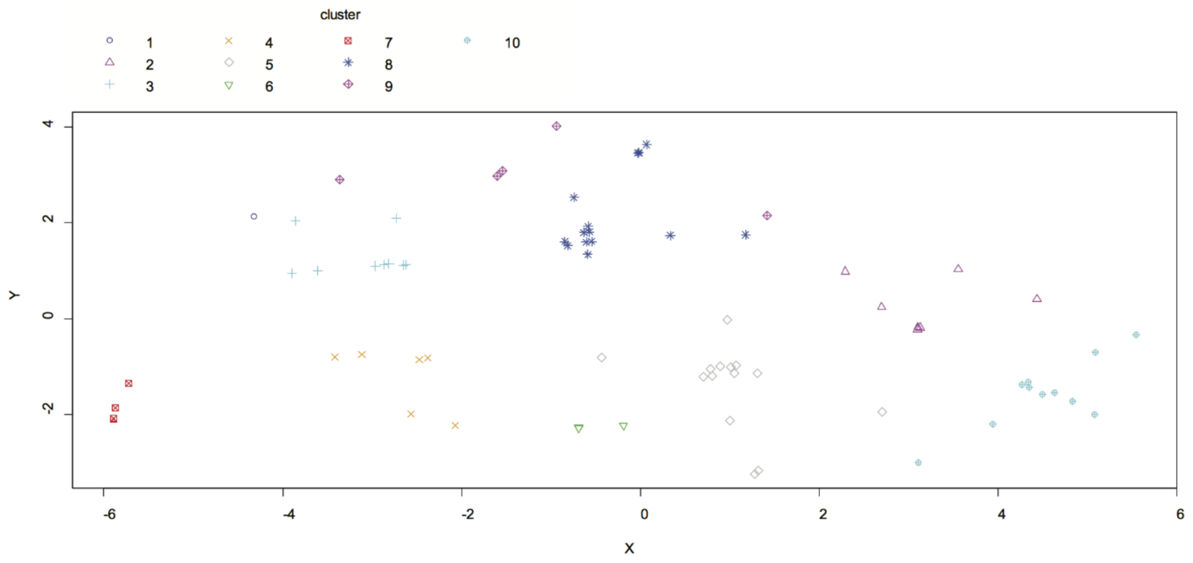

Figure S14. Output of PAM classification. The investigated samples are distributed in 10 clusters. Graph obtained with R software (version 3.4.3, URL http://www.R-project.org/).

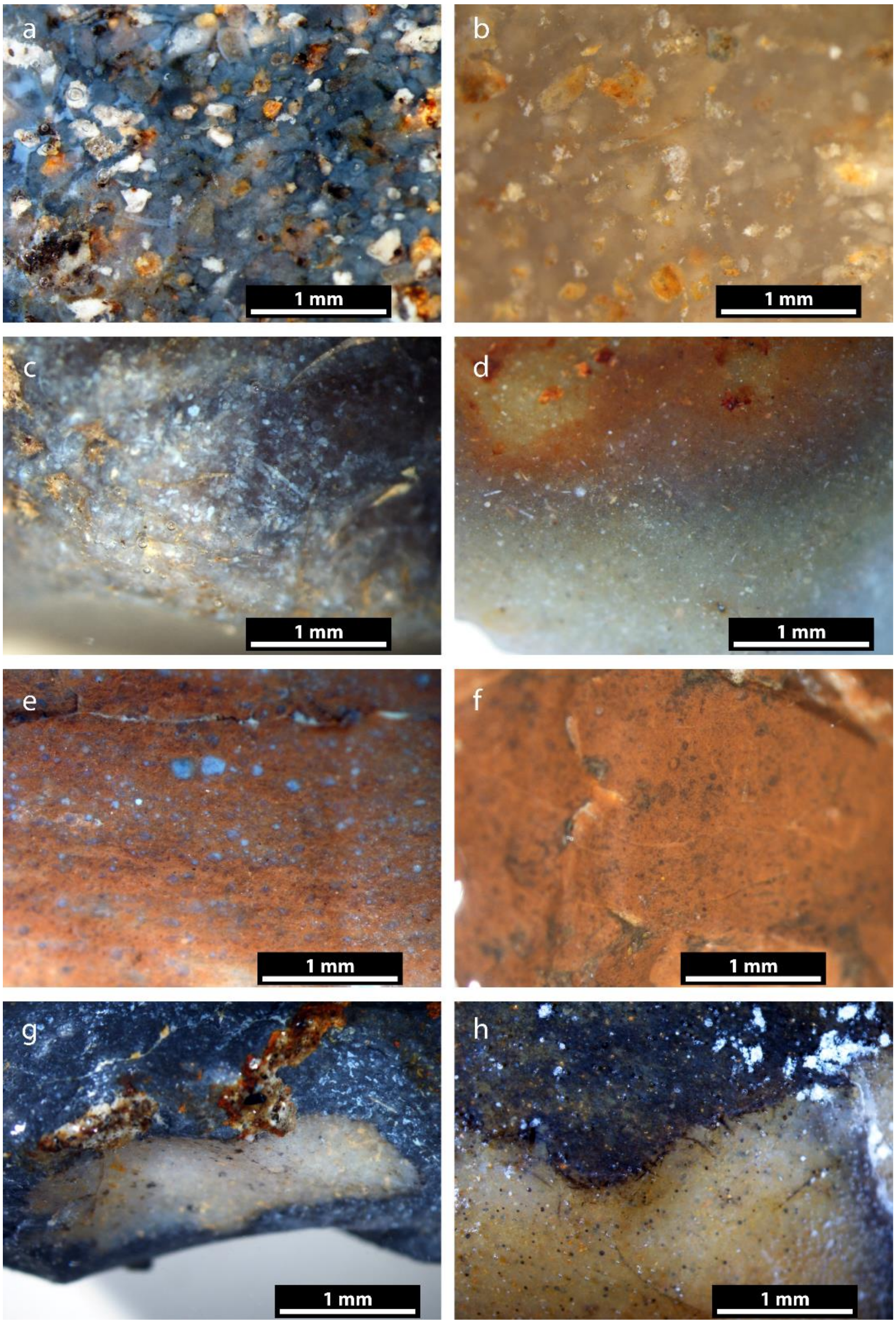

Figure S15. Main structures and textures of the analyzed chert artefacts (magnification = 20x): a) flysch ps (NOT140); b) flysch ps (NOT102); c) flysch ps (NOT12); d) flysch ws (NOT110); e) radiolarite (NOT121); f) radiolarite (NOT16); g) nodular with post-depositional black patina (NOT136); h) nodular with pre-depositional black patina (NOT131).
(photos G.Eramo, I.Allegretta, E.Delluniversità, R.Terzano)

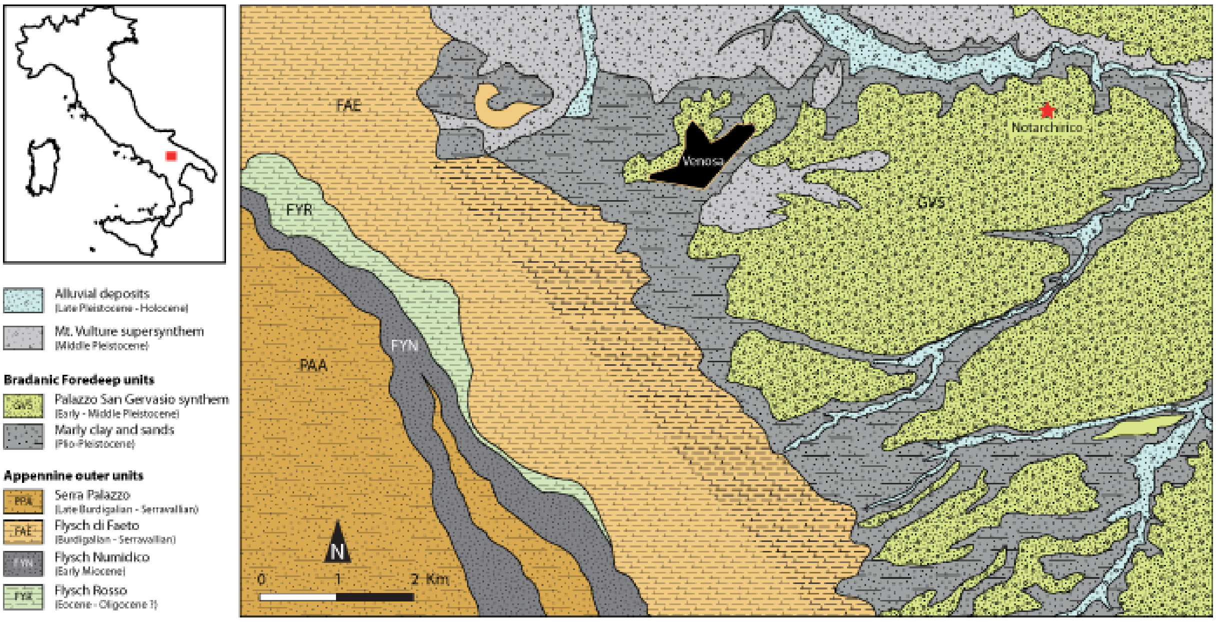

Figure S16. Geological map of the Notarchirico area and potential raw material sources. (map drawn by G. Eramo)

Lithic assemblages

Marie-Hélène Moncel (1), Carmen Santagata (1, 2)

1) Département Homme et Environnement, UMR 7194, HNHP, Muséum National d'Histoire Naturelle, Paris, France.

2) PACEA, UMR CNRS 5199, Université de Bordeaux, Bât B2 Allée Geoffroy St Hilaire, 33615 Pessac cedex (France)

Table S15. Heavy-duty component on limestone pebbles per archaeological layer (number) and flakes in other stones

	J	I2	I1	H	G	F
Unifacial convergent LCT tools		2			6	5
Bifaces					2	4
Unifacial pebble tools	2	5	9	1	15	34
Bifacial pebble tools		1	2		2	6
Pointed unifacial pebble tools			2		6	10
Pointed bifacial pebble tools/LCTs			1			4
Trifacial pebble tools			1		1	
Rabots on pebbles		1			2	5
Quadrangular unifacial tools						2
Broken pebbles with impacts + isolated removals			1		31	52
Flakes		2	2		7	46
Other stone products		1	5	1		4

Table S16. The tool kit on chert: number of flakes, fragments of flakes, retouched nodules and heavy-duty tools per archaeological layer.

	J	I2	I1	H	G	F
Unretouched flakes	5 (4 retouched)	40 (18 retouched)	98 (9 retouched)	21 (7 retouched)	78 (33 retouched)	177 (29 retouched)
Broken flakes-debris		9 (3 retouched)	95 (10 retouched)	19 (6 retouched)	78 (23 retouched)	66 (7 retouched)
Retouched nodules		4	21	1	50	12
Cores	1	6	10		25	10
Bifacial tools					1	1
Bifaces						1

Table S17. Types of cores on chert and limestone per archaeological layer (number).

	J	I2	I1	H	G	F
**Chert**						
Fragments of cores	1		2		12	2
Unifacial cores		5	3		8	5
Bifacial cores			1			1
Orthogonal cores - SSDA type cores			3		6	1
Semi-tournant cores			2		1	
Multifacial cores		1	3		1	2
**Limestone**						
Unifacial cores		1				15
Bifacial cores						4
Unifacial on a nodule edge						1
Orthogonal cores						2
Multifacial cores						

Table S18. Types of flake-tools on chert per archaeological layer (number).

	I2	I1	H	G	F
Points		1	2		
Points « déjetées »					1
Becs			1	2	
Notches				2	
Lateral Retouch	1	6	7	10	7
Bilateral Retouch			1	2	1
Peripheral Retouch	1	4		3	2
Proximal Retouch					1
Distal Retouch	2	2	2	5	
Distal and bilateral Retouch				3	
Distal and lateral Retouch				1	
Distal and proximal Retouch				3	
Convergent Retouch				2	

Table S19. Retouched nodules on chert per archaeological layer (number).

	I2	I1	H	G	F
Points				1	1
Notches			1	1	
Distal Retouches	1	2		9	
Lateral Retouches	1	8		14	4
Bilateral Retouches		2		1	2
Distal and bilateral Retouches				1	1
Convergent Retouches		2		10	3
Peripheral Retouches		4		2	
Semi-Peripheral Retouches		1		2	1

Table S20. Minimum, maximum and average sizes by category of artefacts and by layer

		I2		I1		H2-H1	G		F	
		Flake	Nodule	Flake	Nodule	Flake	Flake	Nodule	Flake	Nodule
Flint products	Min	8	17	7	20	10	11	15	11	19
	Max	59	30	65	49	50	120	47	120	29
	Average	22.2	20.9	23.1	20.5	18.1	24.02	24.1	24.02	18.4
Flint cores	Min	24		7			17		27	
	Max	80		52			81		79	
	Average	42.5		34.1			36.6		42	
Limestone flakes	Min	16		17			13		23	
	Max	103		44			75		114	
	Average	37.8		24.8			49.3		65.08	
Limestone cores	Min								52	
	Max								190	
	Average								99.9	
Pebble tools	Min	33		27			27		38	
	Max	179		179			160		200	
	Average	67.9		58.3			67.04		96.3	

Table S21 Bifaces, unifaces and bifacial tools from layers F and G

Square	Layer	N°	Raw material	Type	L (mm)	W (mm)	T (mm)	Support	Angles Cutting edges (Left to right edge)
A18	F	14	Limestone	Uniface	112	76	41	Pebble	65-92-68
A18	F	13	Limestone	Biface	107	70	45	Ind	44-70-80
B19	F	7	Limestone	Bifacial tool	88	65	43	Pebble	
B17	F	18	Limestone	Uniface	112	71	34	Cortical flake	55-45
A18	F	7	Chert	Bifacial tool	103	75	37	Cortical flake	77-90-73-58
Z16	F	22	Limestone	Bifacial tool	86	58	27	Indet	80-84
A19	F	-	Limestone	Uniface	106	75	56	Pebble	60-67
Z16	F	26	Limestone	Twisted biface	142	76	45	Pebble	
V17	F	10	Limestone	Biface	135	88	57	Pebble	84-94
V16	F	103	Chert	Biface	69	41	33	Nodule	57-69
A23	G	8	Limestone	Partial triface	123	87	62	Pebble	
B23	G	3	Chert	Bifacial tool with a back	99	64	37	Nodule	64-74-90
A24	G	32	Limestone	Uniface convergent	79	61	32	Pebble	88-95-90
B23	G	57	Limestone	Biface/Triface recycling	98	54	42	Pebble	65-72-87
B25	G	18	Limestone	Biface	83	47	32	Pebble	80

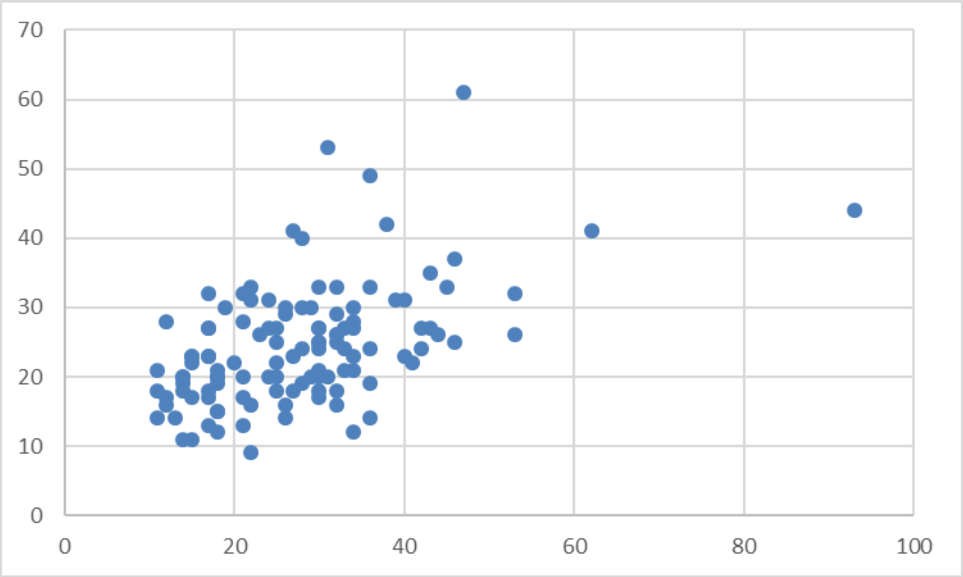

Figure S17. Length/width (in mm) of chert flakes from layer F

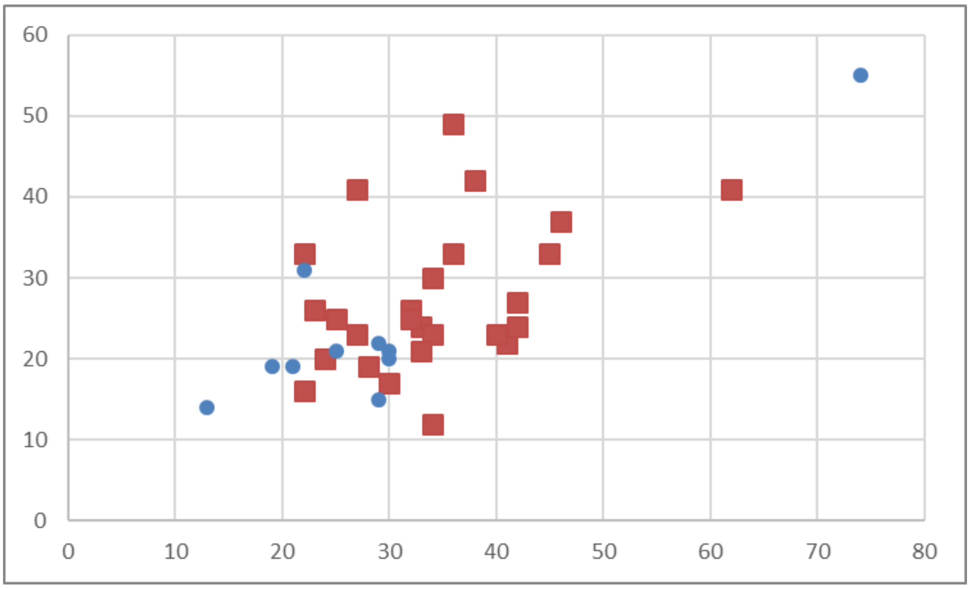

Figure S18. Length/width (in mm) of chert retouched nodules (red squares) and retouched flakes (blue circles) from layer F

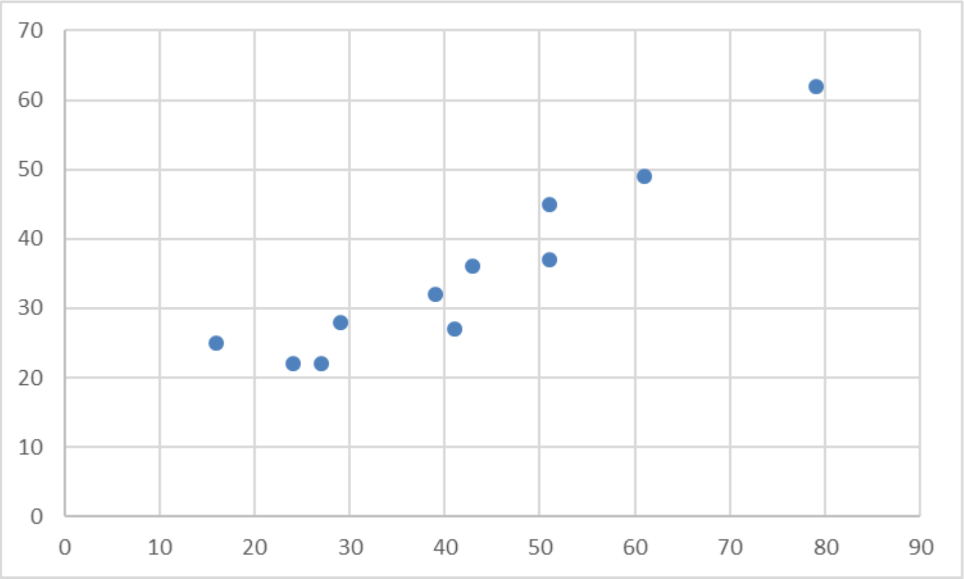

Figure S19. Length/width (in mm) of chert cores from layer F

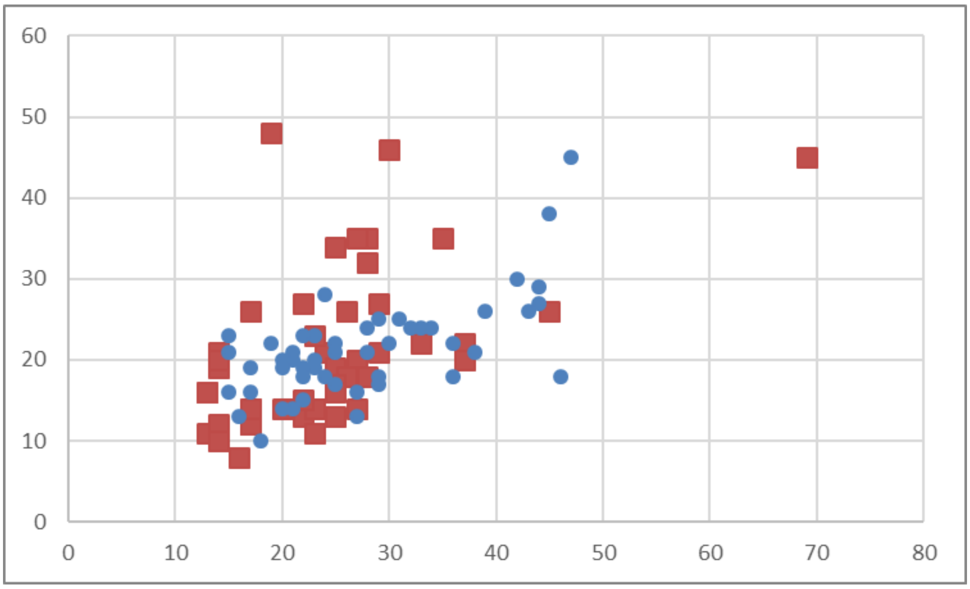

Figure S20. Length/width (in mm) of retouched chert nodules (blue circles) and retouched chert flakes (red squares) for layer G

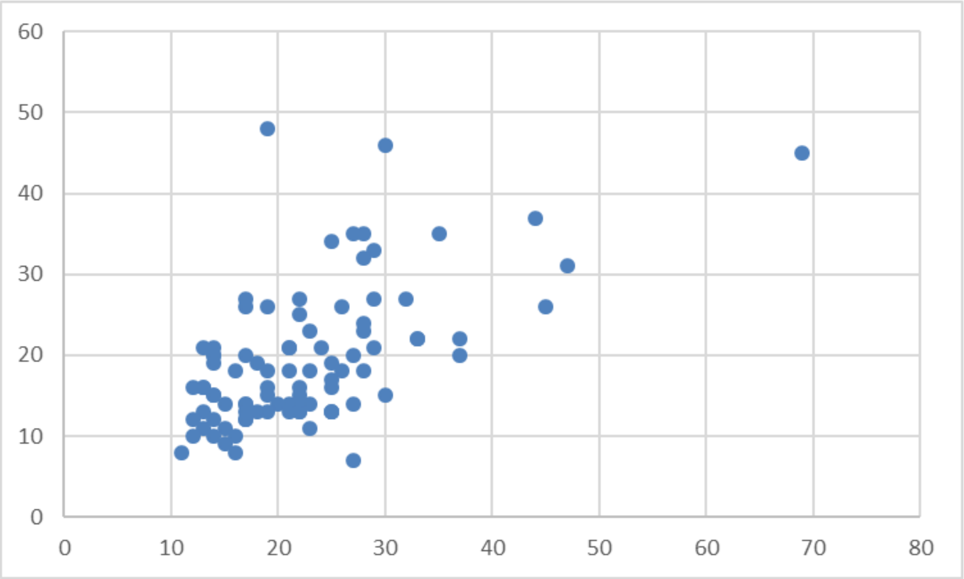

Figure S21. Length/width (in mm) of chert flakes for layer G

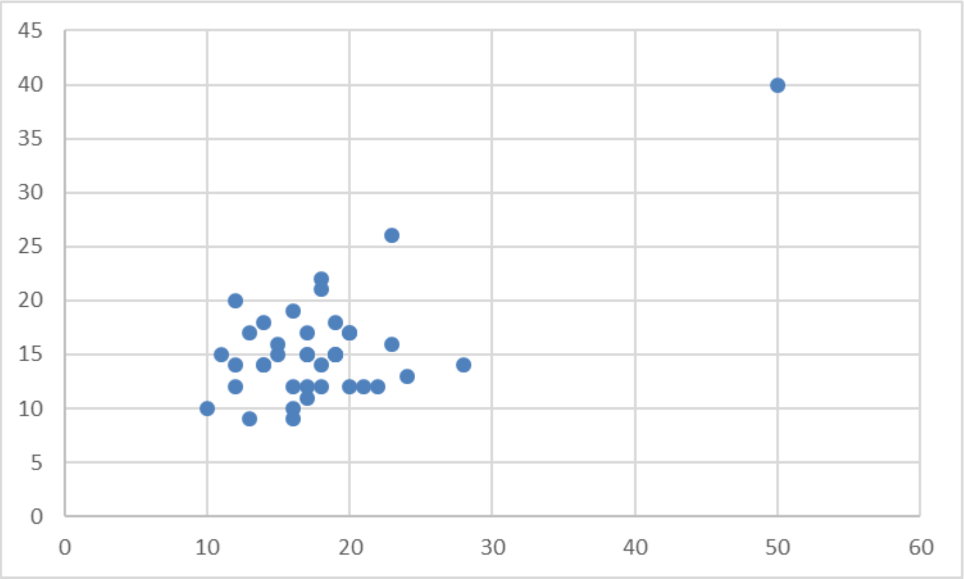

Figure S22. Length/width (in mm) of chert flakes for layer H

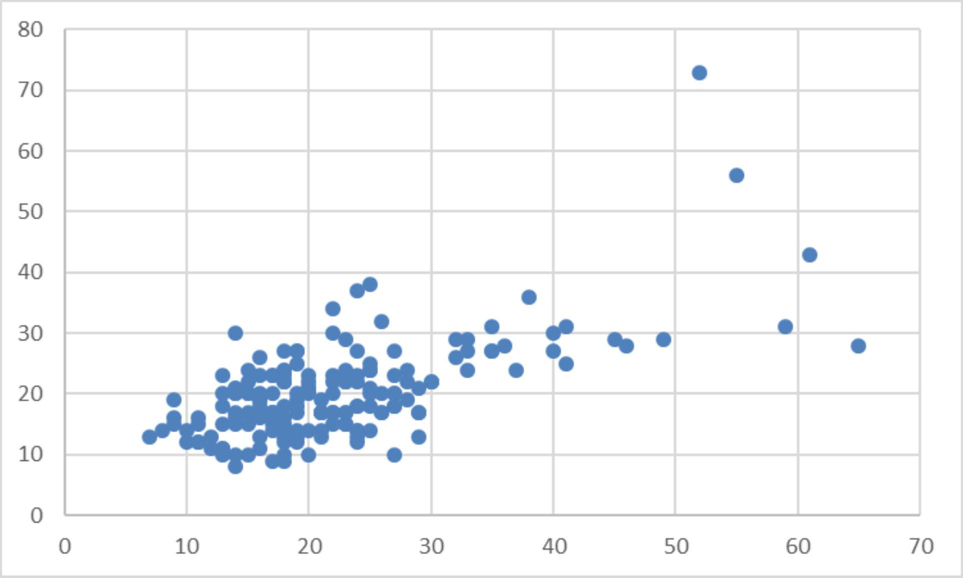

Figure S23. Length/width (in mm) of chert flakes for layers I2 and I1

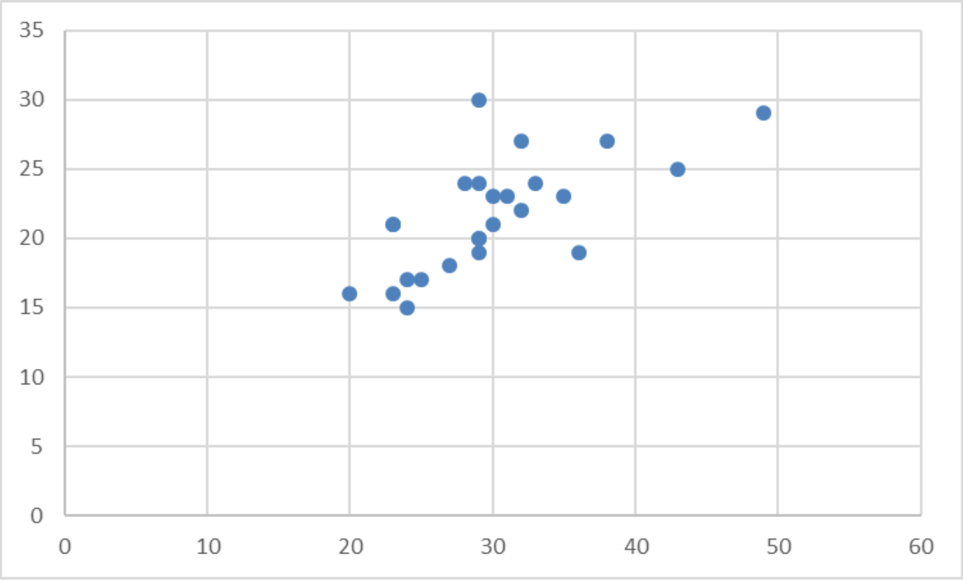

Figure S24. Length/width (in mm) of chert retouched nodules for layers I2 and I1

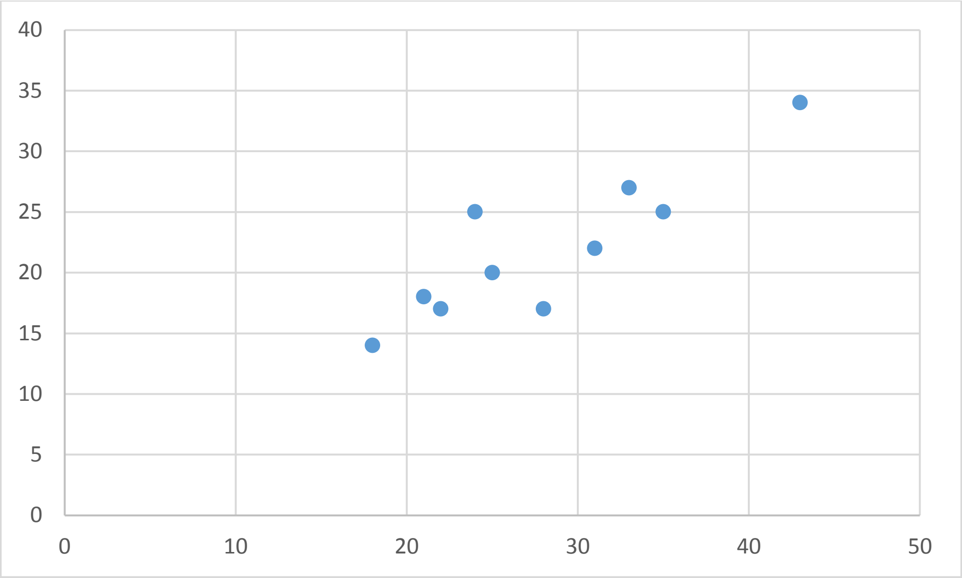

Figure S25. Length/width (in mm) of chert cores for layer I2

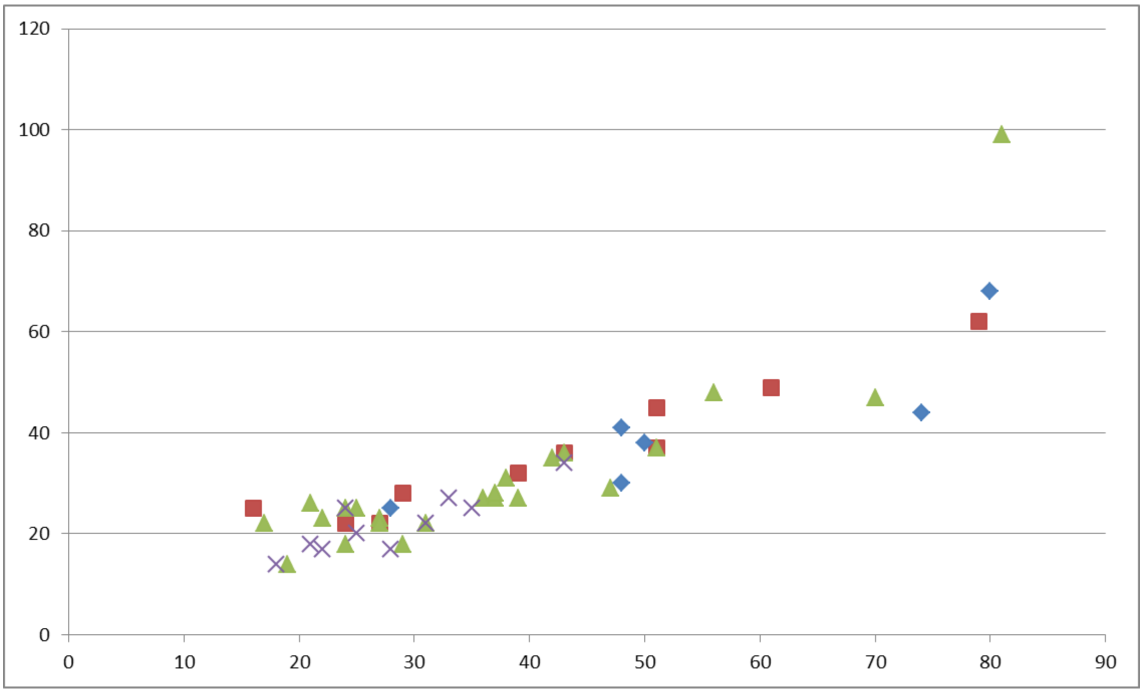

Figure S26. Length/width of chert cores and comparison between layers F, G, I1 and I2
Layer F: red squares, layer G green triangles, layer I1 crosses, layer I1 bleu diamonds

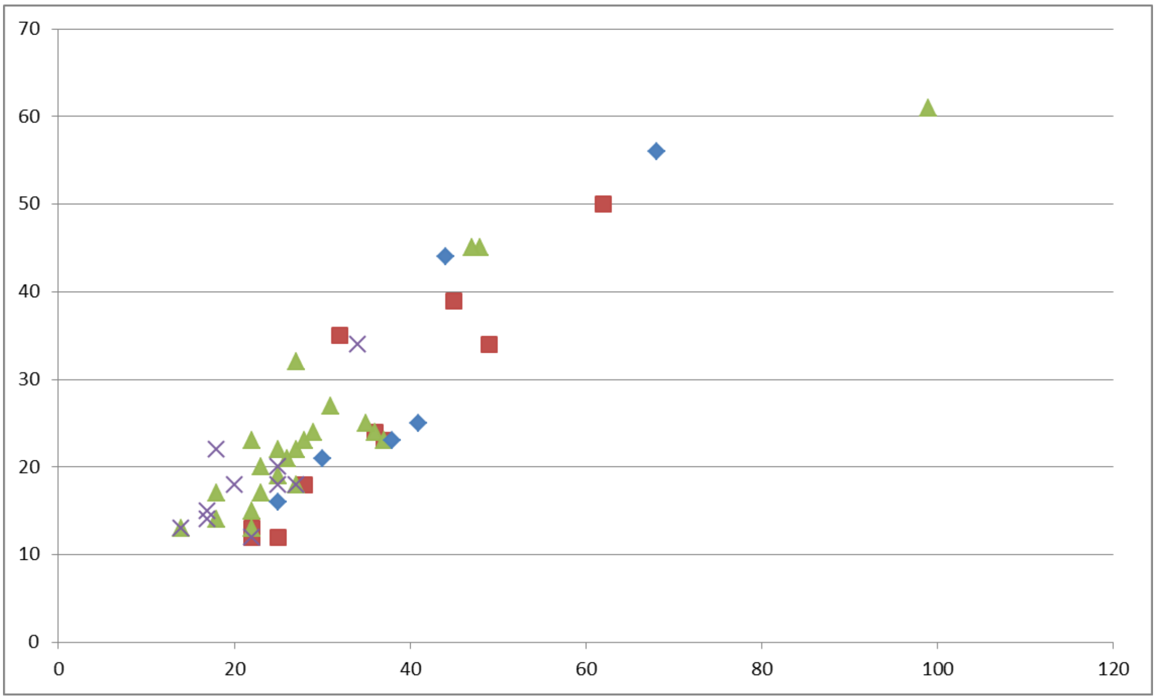

Figure S27. Width/thickness of chert cores and comparison between layers F, G, I1 and I2
Layer F bleu diamonds, layer G green triangles, layer I1 crosses, layer I2 red squares

Detailed description of Large Cutting Tools including the bifaces from layer F

-Partial bifacial tool on a limestone pebble (86 58 27 mm). Z16 n°22 **(*cf.* Fig. 5, n°1)**
Shaping concerns the whole convergent edges by one or two short series of removals. The base is cortical. Shaping by short convergent removals on three-quarters of both edges, with a pointed tip. The edges are sinuous and the tip is pointed. The angles of the cutting edges are 85-87°. There is bilateral and bifacial asymmetry. A few secondary removals suggest recycling. It is a bilateral and bifacial asymmetrical tool.

- Biface on a limestone flat pebble (142-76-45 mm). Z16 n°26 **(*cf.* Fig. 5, n°2)**
Shaping by invasive removals on all edges, except for one back. Short removals worked the tip and one edge, some of which are hinged. The base and the tool center on both faces are cortical. Final retouch is visible on one edge. Cutting edge angles are 64-77°. The tip may be broken. There is a transverse and large removal on the tip.

-Unifacial tool on a limestone cortical flake or flat-quadrangular pebble (112-76-41 mm). A18 n°14 **(*cf.* Fig. 5, n°4)**
Two series of centripetal removals cover almost all of one face (large and small flat removals). The bottom is cortical. The edges are sinusoidal with angles of 85-90°. The tip is round/transversal

-Biface on a limestone (?) flake or pebble (107-70-45 mm). A18 n°13 **(*cf.* Fig. 5, n°5)**
Bifacial asymmetry (curved section). One face is completely shaped by two series of semi-abrupt removals (large then small) while the ventral face is made by removals on one edge and the tip. The volume is managed by alternate removals. There is some localized retouch and crushing marks all around the tool. Cutting edge angles are varied, from 75 -85° and edges are sinusoidal

-Bifacial tool on a limestone pebble (135-88-57 mm). V17 n°10 **(*cf.* Fig. 5, n°6)**
Both faces are worked by a series of invasive removals. Shaping is alternate. The base is cortical. There is an abrupt removal on the edge of the tip (notch-like). The cross-section is asymmetrical

-Bifacial tool on a nodule of chert (69-41-33 mm). V16 n°103 **(*cf.* Fig. 5, n°7)**
Shaping concerns the volume and the two faces by two series of large and short removals. The upper face is more covered by removals than the lower face. The final removals give an asymmetrical tool in cross-section. The edges are sinusoidal. The quality of the chert is poor.

-Unifacial tool on a limestone cortical flake (112-71-34 mm). B17 n°18
One face is covered by convergent short abrupt and semi-abrupt removals located on the upper part of the flake and a transversal edge. There is also a notch. The angles of the cutting edges are 62-86°.

- Bifacial tool on a flint cortical flake (103-75-37). A18 n°7
The upper face is worked by short convergent semi-abrupt removals on three-quarters of the edges. The lower face is worked by large and flat removals covering the face. The base is cortical. The cross-section of the tool is asymmetric. The angles of the cutting edges are 73-75°. Preservation status is poor.

- Unifacial tool on a limestone pebble or flake (106-75-56 mm). A19 NC

Removals are convergent and totally invasive and the tip is large and round. Cutting edge angles are 80-85°. The base is cortical with a back. Some final retouch is located on the tip. The opposite face is flat and cortical.

Detailed description of Large Cutting Tools including bifaces from layer G

- Triface/biface on a limestone pebble (98-54-42 mm). B23 n°57 **(*cf.* Fig. 6, n°1)**
It is a recycled tool or core with centripetal smooth removals on one flat face (lower face). The latter shaping phase used the piece as a preform to shape the opposite face by abrupt convergent and more or less invasive removals on both lateral edges and the proximal edge. There are crushing marks on the round tip, which is slightly shaped. Cutting edge angles are 65-72-87°.

-Bifacial tool on nodule of chert (99-64-37 mm). B23 n°3 **(*cf.* Fig. 6, n°2)**
Shaping by bifacial invasive convergent removals on one side and the proximal part. A back is preserved on the opposite side. The angles of the cutting edges are 69-85°. There is notch related to a bec at the tool extremity. Shaping uses the natural shape of the nodule. The tool is asymmetrical in shape and cross-section.

-Biface on a limestone pebble (83-47-32 mm). B25 n°18 **(*cf.* Fig. 6, n°3)**
Shaping is alternate, managing the volume by invasive and small removals on the upper face and part of the lower face. The tip is round/pointed. Edges are sinusoidal (80°). State of preservation is poor (smooth surface). The shape is symmetrical.

-Unifacial convergent tool on a thick limestone pebble (79-61-32 mm). A24 n°32
Shaping by convergent and semi-abrupt short removals on more than half of the length for one edge and half of the opposite edge. The tip is pointed.

Micro-wear study

Cristina Lemorini

LTFAPA Laboratorio, Dipartimento di Scienze dell Antichità, Sapienza Università di Roma

Use-wear analysis was carried out combining the Low-Power (LPA) and the High-Power Approaches (HPA) in order to obtain a detailed interpretation of the function of the chipped stone tools. The LPA analyzes traces of use observable at low magnification (generally up to 70X-80X), defined in the literature as macro-traces and, for the lithic industry made of flint, consist of: edge removals (use scars) and edge rounding. The HPA analyzes traces of use observable at high magnification (starting from 100X), defined in the literature as micro-traces and, for the lithic industry made of flint, consist of micro edge rounding, polishes and striations. The LPA was applied to the chipped stone tools of Notarchirico using a Nikon SMZ stereomicroscope with a magnification range of 0.75X to 7.5X, equipped with a 10X ocular, a 1X objective, a reflected light system and a ToupView CMOS. The LPA enabled us to evaluate the state of conservation of the lithic industry, to sample the tools with macro-wear and to infer the activities carried out and the hardness of the materials processed with them. The HPA was applied using a Nikon Eclipse metallographic microscope, oculars 10X, 20X, 50X equipped with a ToupView CMOS Camera. Helicon Focus software was used for picture focus stacking. The HPA was also applied with a digital microscope Hirox RH-2000 to observe highly reflective surfaces and to obtain pictures with ultra-fine detail. The observation of micro-wear was carried out at the LTFAPA laboratory on molds of the lithic surface made with an ultra-fine two-component silicone, Provil Novo Light Fast, Heraeus.
Before the microscopic observation and before the molding process, the lithic tools were gently washed with warm tap water.
The interpretation of use-wear was made using the comparative reference collection of flint replicas from the LTFAPA laboratory of the Sapienza University.

The chipped stone tools of Notarchirico are affected by post-depositional processes that have strongly hampered the possibility to observe micro-wear and to infer in detail the activities carried out with these artefacts. Their surface is very bright and, under the microscope, it reveals a glossy topography sometime furrowed by thin striations. The gloss is a consequence of the mechanical action of the water flow, which was a major factor in the formation of the deposit. The "cratered" glossy topography observable on some of the artifacts also suggests the presence of chemical agents that started a process of raw material desilicification (**Fig. S26**). The thin striations observed on various glossy surfaces show that abrasive process affected the lithic surface, in the form of sandy particles in suspension in the water flow (see also the taphonomic process observed on the fauna). Some mechanical alterations producing scars and breakage were observed on the chipped stone tools of Notarchirico, especially in level F. Therefore, edge removals and edge rounding were well preserved and well visible, rendering possible macro-wear analysis and the interpretation of the activities carried out and the hardness of the worked materials **(Fig. S27**).

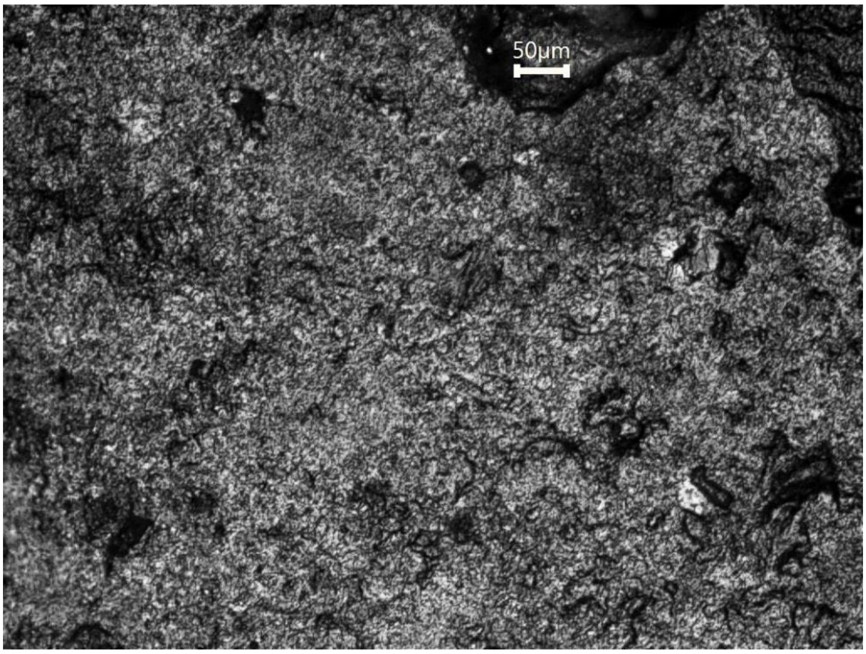

Figure S28. Exemple of alterated lithic surface, item 2016 B42 J2 (photo C. Lemorini)

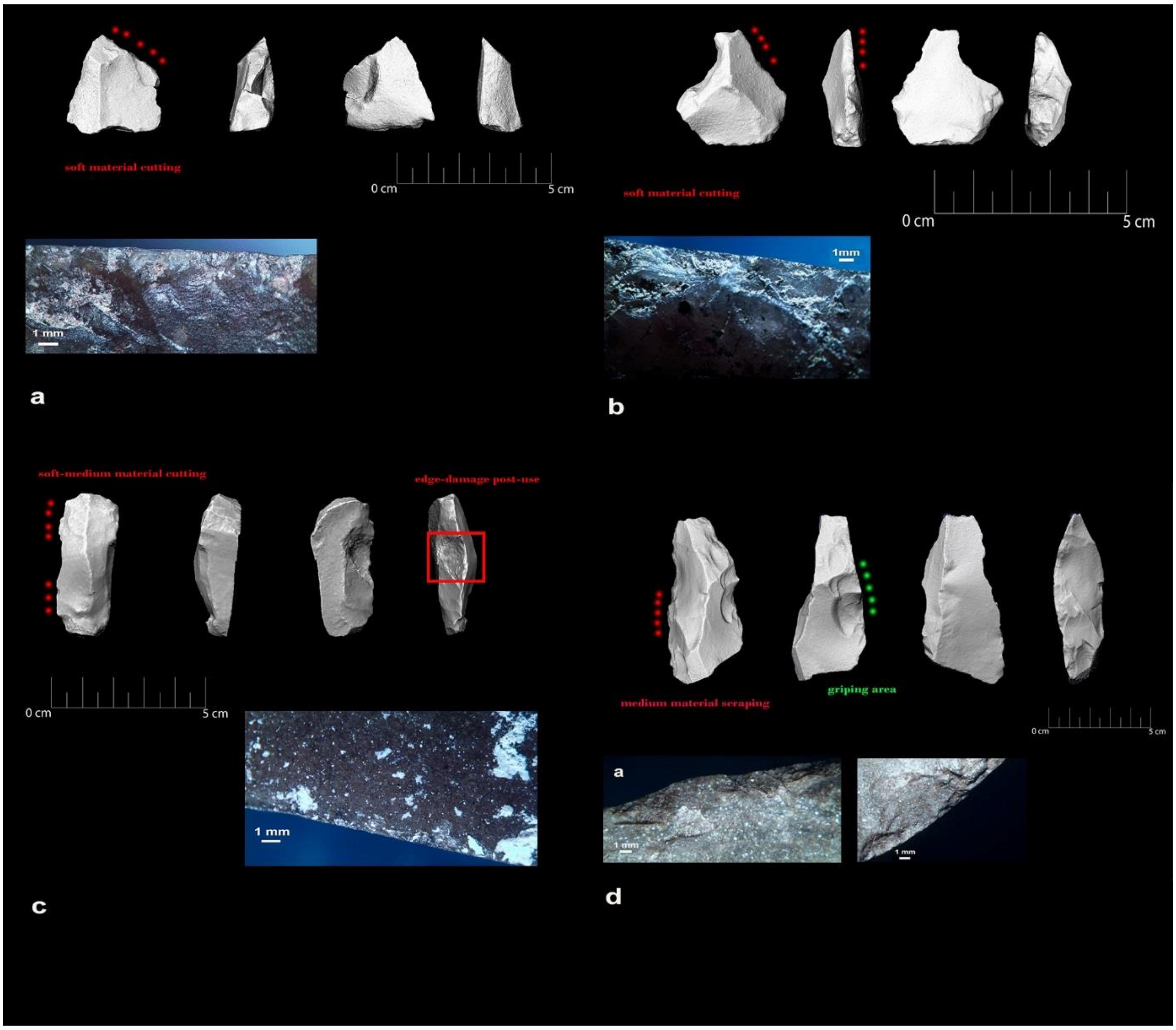

Figure S29. a) B38 I2 retouched flake; edge removals with close-regular distribution, longitudinal unidirectional orientation, feather terminations interpreted as cutting soft material; b) B36 I1c 19 flake; edge removals with close-regular distribution, longitudinal unidirectional orientation, feather terminations interpreted as cutting soft material; c) A33 H2a 22 flake; edge removals with close-regular distribution, longitudinal unidirectional orientation, feather/step terminations interpreted as cutting soft-medium material; d) B42 J2 flake; edge removals with close-regular distribution, perpendicular orientation, feather/hinge/step terminations interpreted as scraping medium material.

(photos C. Lemorini)

Preliminary residue analysis

Bruce Hardy

Kenyon College, Ohio, USA

A preliminary sample of 50 stone artefacts were analyzed microscopically for use-related residues.

Methods

The methods followed are the same as those used in a recent article by Hardy et al. 2018. For residue observation, artefacts were examined with Dino-Lite digital microscopes with magnifications ranging from 20-485x and residues were photographed using Dinocapture 2.0 software. All the observed residues were photographed and their location was noted on a line drawing of each artefact. Residue identification was based on comparisons with a large modern reference collection (over 1,000 experimental artefacts) and with published sources [28]. Identifiable residue categories include wood, bark, plant fibers, starch grains, calcium oxalate crystals, plant tissue, resin, hair, feathers, fish scales, skin and bone. Starch grains can potentially be mistaken for fungal spores or other materials and identification under reflected light is therefore considered preliminary. For all identifications, a suite of related residues (e.g., hair fragments, collagen, bone or plant cells, starch grains, plant fibers) corroborated identification. Calcium oxalate crystals (raphides) can be mistaken for rod-shaped calcite crystals. Putative raphides were treated with acetic acid, which dissolves calcite, to confirm identification. As the main goal of the study was residue analysis, use-wear observations only a played secondary role. Markers of the relative hardness of worked materials and use action included the identification of striations, edge rounding and microflake scars. All artefacts were unwashed prior to analysis. In some cases, a thick coating of sediment covered the artefact, entirely concealing the surface. Such artefacts were immersed in still water to remove excess sediments. The artefacts were then left to air dry prior to analysis.

All residues observed were photographed and their location was noted on a line drawing of each artefact. Identification of residues was based on comparisons with a large modern reference collection (over 1,000 experimental artefacts) and with published sources (Brunner and Coman, 1974; Catling and Grayson, 1982; Brom, 1986; Beyries, 1988; Anderson- Gerfaud, 1990; Hoadley, 1990; Teerink, 1991; Fullagar, 1991, 2006; Hather, 1993; Hardy, 1994; Kardulias and Yerkes, 1996; Williamson, 1996; Hardy and Garufi, 1998; Pearsall, 2000; Haslam, 2004; Dove et al., 2005; Huffman et al., 2008; Crowther, 2009; Genten et al., 2009;Warren, 2009). Identifiable residue categories include wood, bark, plant fibers, starch grains, calcium oxalate crystals, plant tissue, resin, hair, feathers, fish scales, skin, and bone (Hardy and Moncel, 2011). Starch grains can potentially be mistaken for fungal spores or other materials and identification under reflected light is therefore considered preliminary (Haslam, 2006). For all identifications, a suite of related residues (e.g., hair fragments, collagen, bone or plant cells, starch grains, plant fibers) strengthened the confidence of identification (Lombard and Wadley, 2007). Calcium oxalate crystals (raphides) can be mistaken for rod-shaped calcite crystals (Crowther, 2009). Putative raphides were treated with acetic acid, which dissolves calcite, to confirm identification. As the main goal of the study was residue analysis, observations of use-wear only played a secondary role. Markers of the relative hardness of the worked material and the use action included the identification of striations, edge rounding and microflake scars (Odell and Odell-Vereecken, 1980; Mansur-Franchomme, 1986).
All artefacts were unwashed prior to analysis. In some cases, a thick coating of sediment covered artefacts, entirely obscuring the surface. Such artefacts were immersed in still water to remove excess sediment (Hardy et al., 2008). The artefact was then allowed to air dry prior to analysis.

Preliminary results

Among the preliminary sample of 50 stone artefacts, a total of 18 flakes and flake fragments presented residues of fragments of plants, wood or plant fibers. A small number of tools presented other possible, but not confirmed residues (hair, feathers or fish muscle). As stone tools typically cross-cut different anatomical planes in plants, it is not always possible to distinguish between woody and non-woody plants. When diagnostic anatomy was not available, identification is limited to the broad plant category (**Fig. S28 to S31**).

References

Anderson-Gerfaud, P., 1990. Aspects of behavior in the Middle Paleolithic: functional analysis of stone tools from southwest France. In: Mellars, P. (Ed.), The Emergence of Modern Humans. Cornell University Press, Ithaca, pp. 389-418.

Braun, D.R., Harris, J.W., Levin, N.E., McCoy, J.T., Herries, A.I., Bamford, M.K., Bishop, L.C., Richmond, B.G., Kibunjia, M., 2010. Early hominin diet included diverse terrestrial and aquatic animals 1.95 Ma in East Turkana, Kenya. Proc.Natl. Acad. Sci. U. S. A. 107 (22), 10002-10007.

Brunner, H., Coman, B.J., 1974. The Identification of Mammalian Hair. Inkata, Melbourne.

Catling, D., Grayson, J., 1982. Identification of Vegetable Fibres. Chapman and Hall, New York.

Crowther, A., 2009. Morphometric analysis of calcium oxalate raphides and assessment of their taxonomic value for archaeological microfossil studies. In: Haslam, M., Robertson, G., Crowther, A., Kirkwood, L., Nugent, S. (Eds.), Archaeological Science under a Microscope [Electronic Resource]: Studies in Residue and Ancient DNA Analysis in Honour of Thomas H. Loy, Terra Australis, vol. 30, pp. 102-128.

Brom, T., 1986. Microscopic identification of feathers and feather fragments of Palearctic birds. Bijdr. Dierkd. 56, 181-204.

Beyries, S., 1988. Functional variability of lithic sets in the Middle Paleolithic. In: Dibble, H., Montet-White, A. (Eds.), Upper Pleistocene Prehistory of Western Eurasia. University Museum Press, Philadelphia, pp. 213-224.

Dove, C.J., Hare, P.G., Heacker, M., 2005. Identification of ancient feather fragments found in melting alpine ice patches in southern Yukon. Arctic 58, 38-43.

Fullagar, R., 1991. The role of silica in polish formation. J. Archaeol. Sci. 18, 1-24.

Fullagar, R., 2006. Starch on artifacts. In: Torrence, R., Barton, H. (Eds.), Ancient Starch Research. Left Coast Press, Walnut Creek, pp. 177-204.

Genten, F., Terwinghe, E., Danguy, A., 2009. Atlas of Fish Histology. Science Publishers, Enfield, New Hampshire.

Hardy, B.L., 1994. Investigations of Stone Tool Function through Use-wear, Residue and DNA Analyses at the Middle Paleolithic Site of La Quina, France (Ph.D. dissertation). Indiana University.

Hardy, B.L., Moncel, M.-H., 2011. Neanderthal use of fish, mammals, birds, starchy plants and wood 125-250,000 Years Ago. PloS One 6 (8), e23768.

Hardy, B. L., Bolus, M. and Conard, N.J. 2008. Hammer or crescent wrench? Stone tool form and function in the Aurignacian of Southwest Germany. Journal of Human Evolution 54:648-662.

Hardy, B.L., Moncel, M-H., Daujeard, C., Fernandes, P., Béarez, P., Desclaux, E., Chacon Navarro, M.G., Puaud, S., and Galotti, R. 2013. Impossible Neanderthals? Making string, throwing projectiles and catching small game during Marine Isotope Stage 4 (Abri du Maras, France). Quaternary Science Reviews 82, 23-40.

Haslam, M., 2004. The decomposition of starch grains in soils: implications for archaeological residue analyses. J. Archaeol. Sci. 31, 1715-1734.

Haslam, M., 2006. Potential misidentification of in situ archaeological tool-residues: starch and conidia. J. Archaeol. Sci. 33, 114-121.

Hather, J., 1993. An Archaeobotanical Guide to Root and Tuber Identification. In: Europe and Southwest Asia, vol. 1. Oxbow Books, Oxford.

Hoadley, B., 1990. Identifying Wood. Taunton Press, Newton, Connecticut.

Huffman, D.M., Tiffany, L.H., Knaphus, G., Healy, R.A., 2008. Mushrooms and Other Fungi of Midcontinental United States. Univ. of Iowa, Iowa City.

Kardulias, N., Yerkes, R., 1996. Microwear and metric analysis of threshing sledge flints from Greece and Cyprus. J. Archaeol. Sci. 23, 657-666.

Lombard, M., Wadley, L., 2007. The morphological identification of micro-residues on stone tools using light microscopy: progress and difficulties based on blind tests. J. Archaeol. Sci. 34, 155-165.

Mansur-Franchomme, M.E., 1986. Microscopie du Matériel Lithique Préhistorique: Traces d'Utilisation, Altération Naturelles, Accidentelles, et Technologiques. CNRS, Paris.

Odell, G., Odell-Vereecken, F., 1980. Verifying the reliability of lithic use-wear assessments by blind tests: the low-power approach. J. Field Archaeol. 7, 87-120.

Pearsall, D., 2000. Paleoethnobotany: a Handbook of Procedures, second ed. Academic Press, New York.

Teerink, B.J., 1991. Hair of West European Mammals: Atlas and Identification Key. Cambridge University Press, Cambridge.

Warren, R., 2009. The Potential Role of Fish in the Diet of Neanderthals. SR. Honors Thesis. Kenyon College, Gambier, Ohio.

Williamson, B., 1996. Preliminary stone tool residue analysis from Rose Cottage Cave. S. Afr. J. Field Archaeol. 5, 36-44.

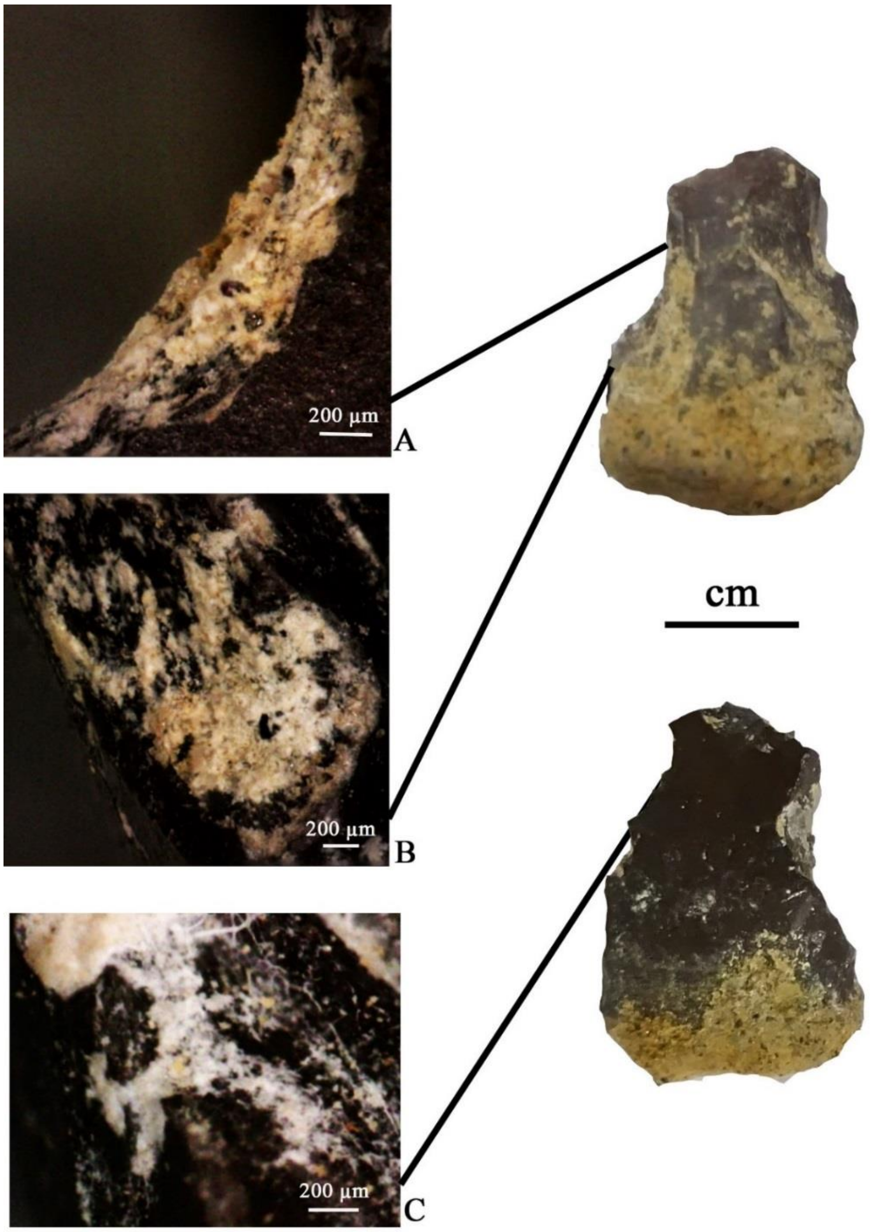

Figure S30. B35 I1 NC6, wood tissue, possibly gymnosperm in cross-section; larger circular areas may be resin canals. (photos B. Hardy)

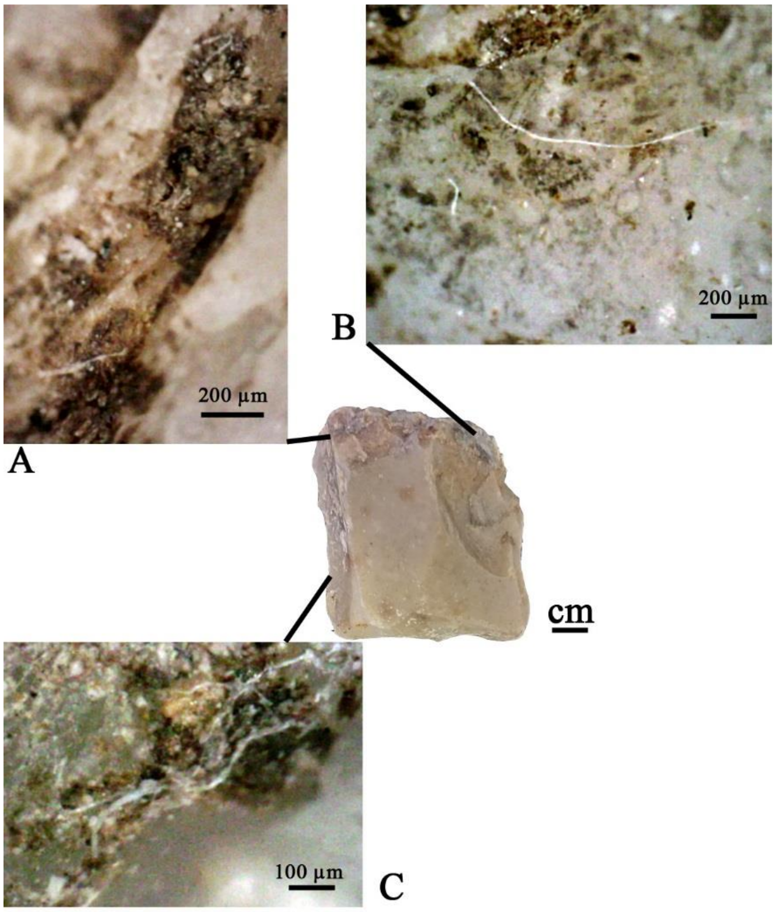

Figure S31. J2 B42, plant fibers on artefact surface. (photos B. Hardy)

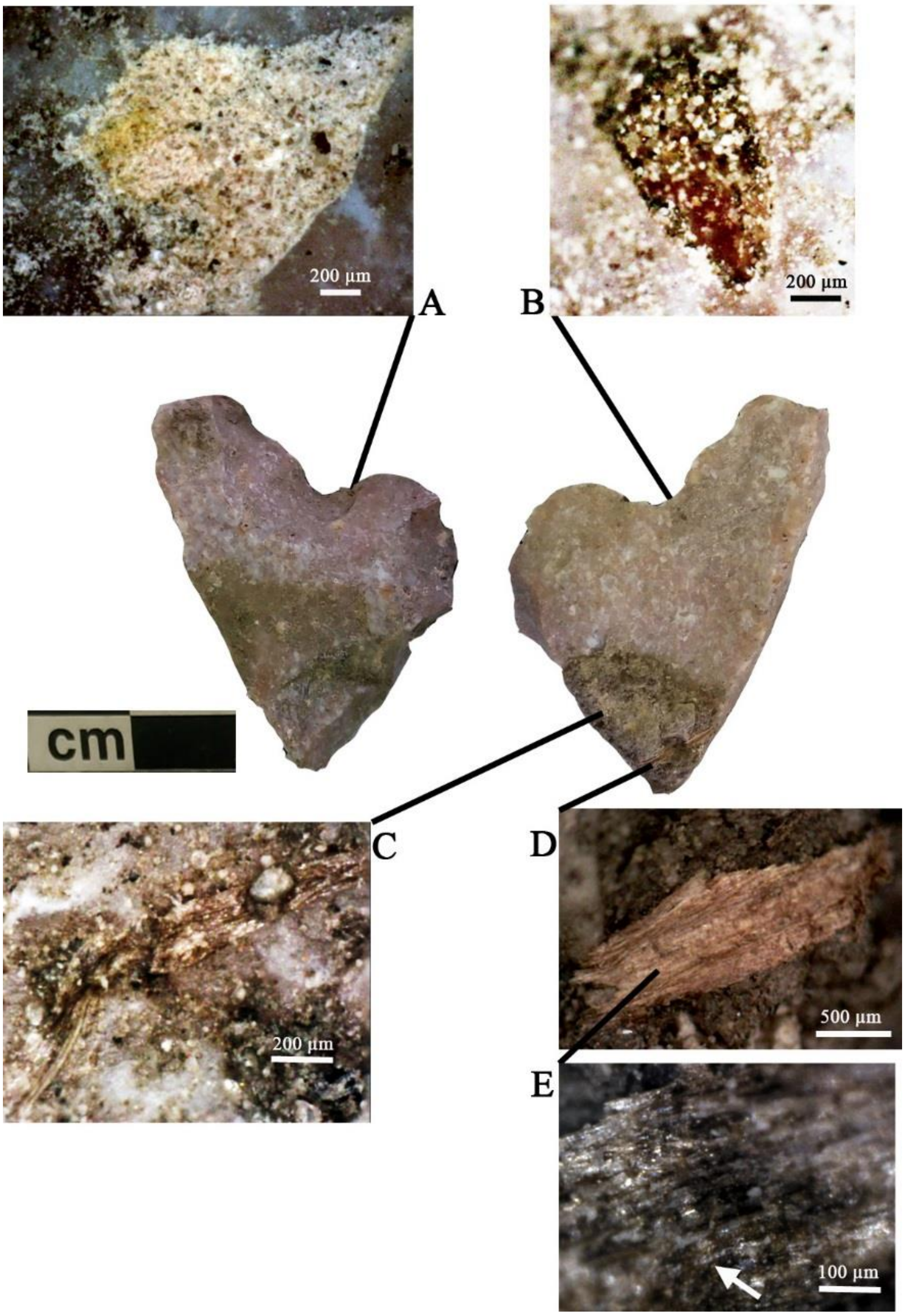

Figure S32. A39 J no.1, Wood fragments, radial or tangential cut, arrow indicates a uniseriate ray. (photos B. Hardy)

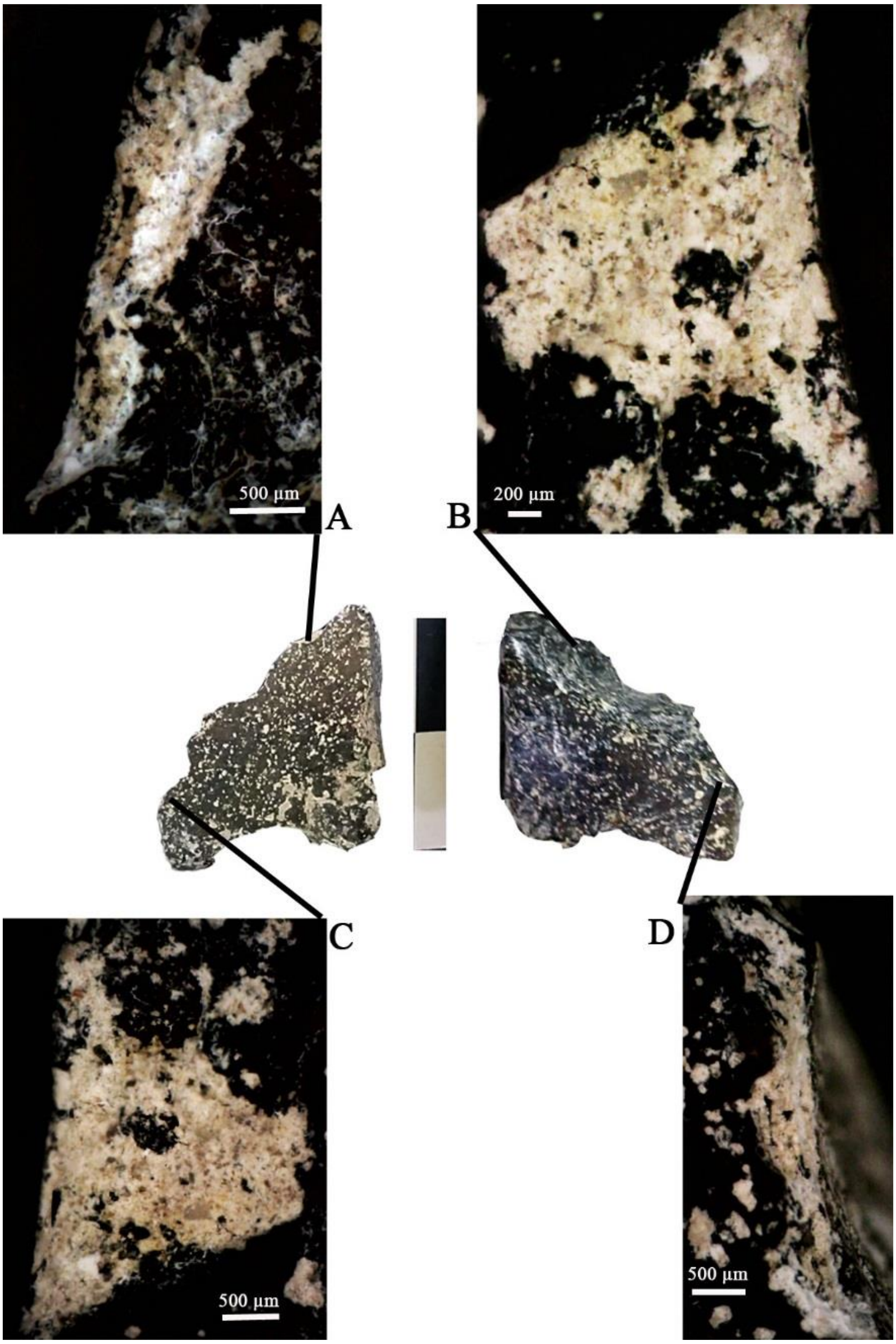

Figure S33. A33 I1B no.35, wood fragments, primarily in cross-section. (photos B. Hardy)
